# Benchmarking the Fluxional Processes of Organometallic Piano-Stool Complexes

**DOI:** 10.3390/molecules26082310

**Published:** 2021-04-16

**Authors:** Nathan C. Frey, Eric Van Dornshuld, Charles Edwin Webster

**Affiliations:** Department of Chemistry, Mississippi State University, 310 President’s Circle, Starkville, MS 39762-9573, USA; ncf74@msstate.edu (N.C.F.); edornshuld@chemistry.msstate.edu (E.V.D.)

**Keywords:** fluxionality, piano-stool complexes, variable-temperature NMR, DFT, ccCA-TM, computational benchmarking and calibration

## Abstract

The correlation consistent Composite Approach for transition metals (ccCA-TM) and density functional theory (DFT) computations have been applied to investigate the fluxional mechanisms of cyclooctatetraene tricarbonyl chromium ((COT)Cr(CO)_3_) and 1,3,5,7-tetramethylcyclooctatetraene tricarbonyl chromium, molybdenum, and tungsten ((TMCOT)*M*(CO)_3_ (*M* = Cr, Mo, and W)) complexes. The geometries of (COT)Cr(CO)_3_ were fully characterized with the PBEPBE, PBE0, B3LYP, and B97-1 functionals with various basis set/ECP combinations, while all investigated (TMCOT)*M*(CO)_3_ complexes were fully characterized with the PBEPBE, PBE0, and B3LYP methods. The energetics of the fluxional dynamics of (COT)Cr(CO)_3_ were examined using the correlation consistent Composite Approach for transition metals (ccCA-TM) to provide reliable energy benchmarks for corresponding DFT results. The PBE0/BS1 results are in semiquantitative agreement with the ccCA-TM results. Various transition states were identified for the fluxional processes of (COT)Cr(CO)_3_. The PBEPBE/BS1 energetics indicate that the 1,2-shift is the lowest energy fluxional process, while the B3LYP/BS1 energetics (where BS1 = H, C, O: 6-31G(d′); *M*: mod-LANL2DZ(f)-ECP) indicate the 1,3-shift having a lower electronic energy of activation than the 1,2-shift by 2.9 kcal mol^−1^. Notably, PBE0/BS1 describes the (CO)_3_ rotation to be the lowest energy process, followed by the 1,3-shift. Six transition states have been identified in the fluxional processes of each of the (TMCOT)*M*(CO)_3_ complexes (except for (TMCOT)W(CO)_3_), two of which are 1,2-shift transition states. The lowest-energy fluxional process of each (TMCOT)*M*(CO)_3_ complex (computed with the PBE0 functional) has a Δ*G*^‡^ of 12.6, 12.8, and 13.2 kcal mol^−1^ for Cr, Mo, and W complexes, respectively. Good agreement was observed between the experimental and computed ^1^H-NMR and ^13^C-NMR chemical shifts for (TMCOT)Cr(CO)_3_ and (TMCOT)Mo(CO)_3_ at three different temperature regimes, with coalescence of chemically equivalent groups at higher temperatures.

## 1. Introduction

Fluxional molecules are dynamic compounds in which magnetically or chemically distinct groups can readily interchange positions. The stereochemically fluid nature of such molecules at room temperature is illustrated by the fluxional shifts that they undergo [1,2,3]. These systems perform a significant role in increasing enantioselectivity in asymmetric synthesis [4,5,6,7,8,9,10]. CpRu((R)-BINOP-F)(H_2_O)][SbF_6_] has been used as a catalyst in the Diels–Alder reaction of methacrolein and cyclopentadiene to produce a [4+2] cycloadduct with enantioselectivity of 92% ee (exo) [9]. During this reaction, the catalyst was shown to exhibit a fluxional pendular motion of the BINOP-F ligand, thereby creating chemically equivalent environments about the two phosphorus substituents.

Density functional theory (DFT) is a useful tool used to characterize the details in the fluxional behavior of various complexes [11]. Previous studies incorporating DFT on fluxional systems range from biochemical applications, such as Cu (II)⋯GlyHisLys peptide binding [12] to understanding fluxionally chiral dimethylaminopyridine catalysts [4,10]. Haptotropic rearrangement processes in sandwich-type complexes have also been investigated by DFT approaches [13,14,15]. Similarly, DFT has been used to gain insight into the fluxionality of various ligands, such as phosphines, in transition metal complexes by way of simulated NMR spectroscopy [16,17].

Although DFT has been used to characterize the energetics of fluxional processes, composite approaches have yet to be utilized to calibrate DFT results on these systems. The correlation consistent Composite Approach for transition metals (ccCA-TM) has been previously utilized to benchmark energetics of transition metal complexes [18,19,20,21,22,23,24,25], and it has been shown to have a mean absolute deviation (MAD) from experiment of 3.0 kcal mol^−1^ (“transition-metal chemical accuracy”). The ccCA-TM methodology was utilized in this study of cyclooctatetraene chromium tricarbonyl ((COT)Cr(CO)_3_) to provide reliable energies for which to compare DFT results.

**Figure 1:**
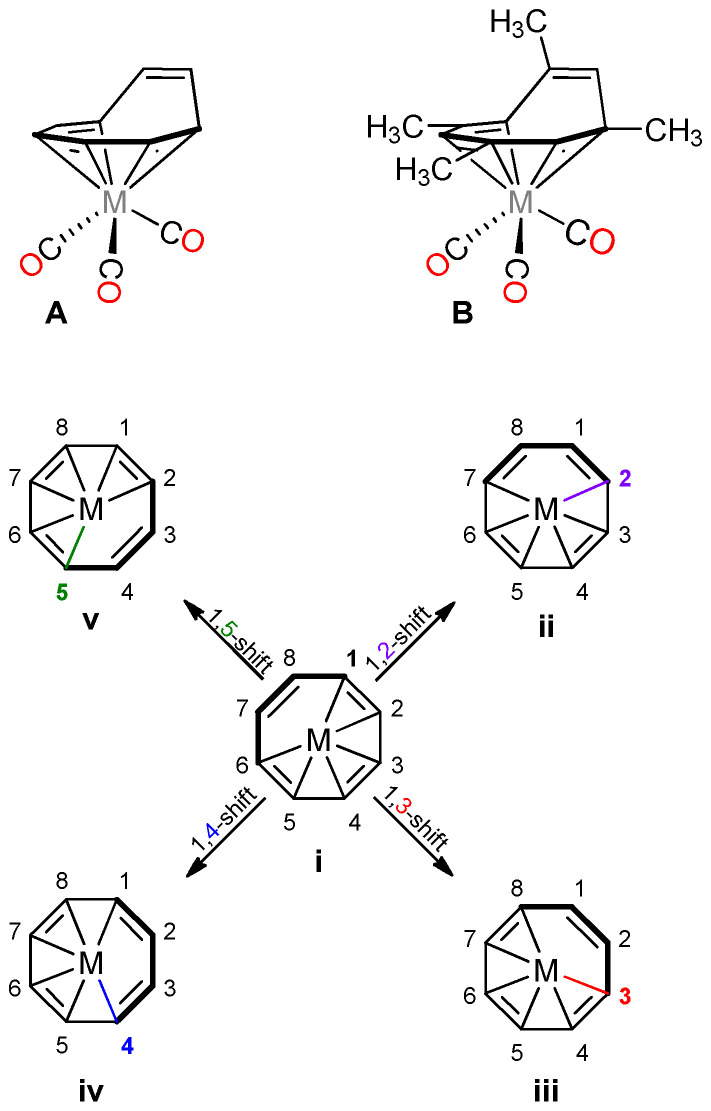
Two-dimensional representations of generalized COT (**A**) and TMCOT (**B**) piano-stool complexes. 1,*n*-shift (*n* = 2, 3, 4, 5) in (COT)*M*(CO)_3_/(TMCOT)*M*(CO)_3_. CO, H, and CH_3_ groups omitted for clarity (**i**–**v**).

Cyclooctatetraene tricarbonyl *d^4^* complexes ((COT)*M*(CO)_3_) are fluxional molecules that contain an η^6^-bound COT ligand that results in a “piano-stool” conformation (Figure 1A) [26]. In order to be comprehensive and specific, we provide, in Figure 1, an explicit accounting of the possible shifts in COT-type complexes. The lowest energy geometry for the (COT)*M*(CO)_3_ molecule is a piano-stool structure (**A** in Figure 1). These complexes can undergo a variety of fluxional shifts in which metal–COT carbon interactions are disrupted and then bound on a new carbon of the COT ligand. These processes can be denoted as a 1,*n*-shift (*n* = 2, 3, 4, 5), where *n* represents the carbon on the ring to which the reference bond on the ring has moved. For example, a 1,2-shift indicates a bonding rearrangement from the parent configuration ([1–6]-η^6^ geometry, **i** in Figure 1) to another η^6^ configuration ([2–7]-η^6^ geometry, **ii** in Figure 1). Therefore, a 1,3-shift would result in the parent [1–6]-η^6^ geometry rearranging to the [3–8]-η^6^ geometry (**iii** in Figure 1).

Historical studies demonstrate that variable temperature NMR (VT-NMR) is a pivotal tool in understanding fluxional behavior [27]. For example, VT ^1^H-NMR spectra provide insight into the energetics of the valency tautomerism of COT ligands about the metal center of (COT)*M*(CO)_3_ (*M* = Cr, Mo) (Δ*G*^‡^ = 15.4 kcal mol^−1^ (*k* ≈ 25 s^−1^) at 20 °C for (COT)Cr(CO)_3_ and Δ*G*^‡^ = 14.8 kcal mol^−1^ (*k* ≈ 25 s^−1^) at 10 °C for (COT)Mo(CO)_3_ [28]. The pioneering work of Cotton and coworkers proposed mechanisms for these low energy rearrangement processes [1,26,27,28,29,30]. Whitesides and Budnik successfully studied the Mo derivative ten years later to arrive at similar conclusions [31]. Spin-saturation [32] and 2D-EXSY [33] have been used to understand fluxional processes. In addition, Lawless and Marynick’s study provided insights from semiempirical computations into the ring rearrangement processes for (COT)Cr(CO)_3_ [34]. In this study, we apply the robust ccCA-TM approach alongside DFT to provide insight into the ring-rearrangement processes of (COT)Cr(CO)_3_. Various ring-rearrangement pathways, including 1,2-, 1,3-, 1,4-, and 1,5-shifts, and (CO)_3_ rotation about the metal center, will be considered in the fluxional processes of these complexes. Herein, we report a study of the energetics of (COT)Cr(CO)_3_ and (TMCOT)*M*(CO)_3_ (*M* = Cr, Mo, W), and an analysis of VT-NMR spectra for (TMCOT)Cr(CO)_3_ and (TMCOT)Mo(CO)_3_.

## 2. Results

**Figure 2:**
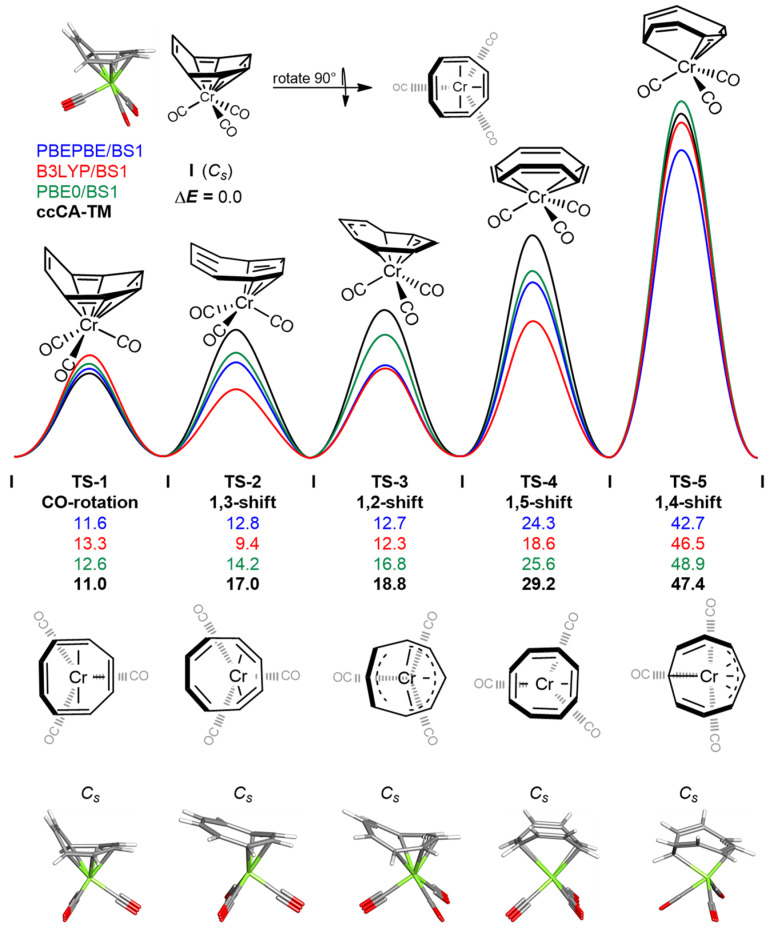
Potential energy surface for the ring rearrangement processes of (COT)Cr(CO)_3_ at various levels of theory. Relative electronic energies (Δ*E_e_*^‡^) are given in kcal mol^−1^. Transition states are ordered in reference to Δ*E_e_*^‡^ values derived from ccCA-TM.

Figure 2 is the potential energy surface for the ring rearrangement processes of (COT)Cr(CO)_3_ at various levels of theory, in which one minimum energy structure (**I**) and five transition states (**TS-1** to **TS-5**) were identified. Δ*E_e_*^‡^ values for the following transition states are given at the PBEPBE/BS1, B3LYP/BS1, and PBE0/BS1 levels of theory (see Methodology for basis set definitions). Additionally, given are the Δ*E_e_*^‡^ values derived using ccCA-TM. **TS-1** is *C_s_*-symmetric, where the COT ligand is η^6^-bound to Cr. This transition state represents the 120° rotation of the three carbonyl groups about the metal center. **TS-2** (1,3-shift) is a *C_s_*-symmetric complex where the COT ligand is η^4^-bound to Cr. **TS-3** (1,2-shift) is *C_s_*-symmetric structure such that the COT ligand is η^5^-bound to Cr. **TS-4** (1,5-shift) is a *C_s_*-symmetric complex, which possesses an η^4^-bound COT ligand. The highest-energy fluxional transition state, **TS-5** (1,4-shift), is a *C_s_*-symmetric complex, where the COT ligand is η^4^-bound to Cr. Good agreement was observed in the relative Δ*E_e_*^‡^ values computed using PBE0/BS1 and those derived using ccCA-TM. Tabulated bond lengths, Δ*G*^‡^, Δ*E_e_*^‡^, and ΔΔ*E_e_*^‡^ (relative to ccCA-TM) values computed at each level of theory are given in the Supporting Information (Table S1, Table S2, Table S3, and Table S4, respectively). Method and basis set testing was performed for each (**I**, **TS-1**–**TS-5**).

Additionally, identified were two additional geometries, structure **2** and **TS-6** (see Figure S4). Structure **2** is a local minimum (Δ*E_e_* = 24.3 kcal mol^−1^ using the PBE0/BS1 level of theory) connected to **I** by way of **TS-6** (Δ*E_e_*^‡^ = 25.3 kcal mol^−1^). Because **2** is higher in energy than **I**, it was not considered in the fluxional processes of (COT)Cr(CO)_3_.

**Figure 3:**
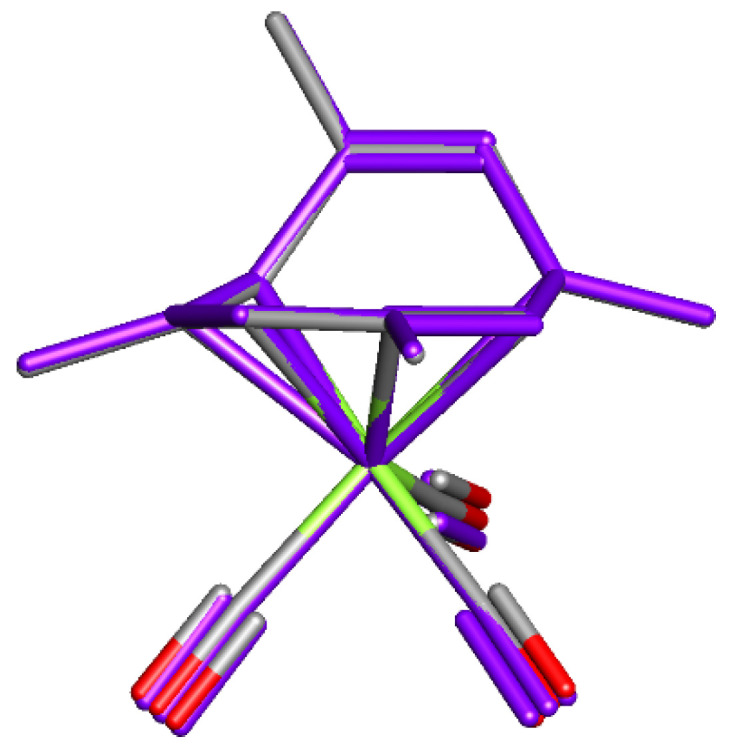
Overlay of the geometry from the X-ray crystal structure (purple) with the PBE0/BS1 optimized structure of (TMCOT)Cr(CO)_3_. Cr: green, O: red, C: grey (H omitted for clarity).

The TMCOT ligand in the X-ray crystal structure of (TMCOT)Cr(CO)_3_ reported by Cotton and coworkers is η^6^-bound to Cr. An overlay of experimental and PBE0/BS1 optimized structures is shown in Figure 3.

The XRD structure (CSD entry: TMCOCR) [29] matches well with the optimized geometry of the lowest energy structure (**II**) for (TMCOT)Cr(CO)_3_ (RMSD = 0.038 Å for the 19 heavy atoms using PBE0/BS1 optimized structure). The X-ray crystal structures for the Mo and W complexes have not been reported. However, the computed structures for these derivatives each contain a η^6^-bound TMCOT ligand and appear to be very similar to the lowest-energy structure computed for the Cr complex (RMSD = 0.146 Å for Mo, 1.181 Å for W relative to (TMCOT)Cr(CO)_3_ for 19 heavy atoms; RMSD calculated using PBE0/BS1 optimized structures).

**Figure 4:**
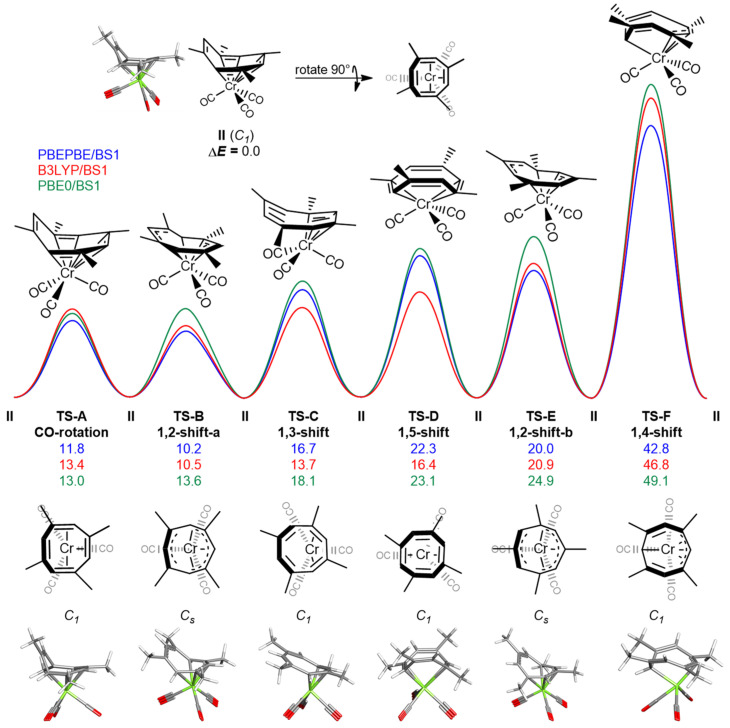
Potential energy surface for various rearrangements in (TMCOT)Cr(CO)_3_. Relative electronic energies are given in kcal mol^−1^. Transition states are ordered in reference to Δ*E_e_*^‡^ values computed using PBE0/BS1.

The potential energy surface for the various rearrangements processes for (TMCOT)Cr(CO)_3_ is given in Figure 4. The lowest-energy geometry is represented by structure **II**. Δ*E_e_*^‡^ values for the described transition states are given at the: PBEPBE/BS1, B3LYP/BS1, and PBE0/BS1 levels of theory for (TMCOT)Cr(CO)_3_. **TS-A** (CO-rotation) is *C_1_*-symmetric and represents a 120° (CO)_3_ rotation about Cr, where the TMCOT ligand remains η^6^-bound to the Cr. The lowest energy fluxional transition state, **TS-B** (1,2-shift-a), is a *C_s_*-symmetric structure in which TMCOT is η^5^-bound to Cr. In **TS-B**, the mirror plane passes through two of the opposing C-H units in the TMCOT ligand. **TS-C** (1,3-shift) is *C_1_*-symmetric, in which the TMCOT ligand is η^4^-bound to the Cr atom. **TS-D** (1,5-shift) is a *C_1_*-symmetric structure in which TMCOT is η^4^-bound to the metal. **TS-E** (1,2-shift-b) is a *C_s_* symmetric structure in which TMCOT is η^5^-bound to the Cr center and represents a second 1,2-shift. The mirror plane in this structure passes through the opposing C–CH_3_ units, in contrast to the opposing C–H units as seen in **TS-A**. **TS-F** (1,4-shift) represents the transition state in which the complex is *C_1_*-symmetric with an η^4^-bound TMCOT ligand.

Table 1 lists the computed relative electronic energies and Gibbs free energies for the different transition states of the (TMCOT)*M*(CO)_3_ complexes computed with the PBEPBE, B3LYP, and PBE0 methods using BS1. Notably, PBE0/BS1 computed energies show that **TS-D** for (TMCOT)W(CO)_3_ is 2.5 kcal mol^−1^ higher in energy than **TS-E**, deviating in the relative Δ*E_e_*^‡^ ordering depicted in (TMCOT)Cr(CO)_3_ and (TMCOT)Mo(CO)_3_. Additionally, there are insignificant differences in the relative energetic ordering of the fluxional processes of each (TMCOT)*M*(CO)_3_ complex, depending on the level of theory implemented. The computed relative electronic energies for (TMCOT)*M*(CO)_3_ and (COT)Cr(CO)_3_ can be found in Table 1 and Table S3, respectively. An additional 1,4-shift transition state (**TS-G**) was located computationally for (TMCOT)W(CO)_3_ (Figure S3).

## 3. Discussion

Several DFT methods utilized for the (COT)Cr(CO)_3_ system disagreed with the relative energy ordering of the fluxional processes obtained with the more rigorous ccCA-TM methodology. It is helpful to determine which methodology is in closest agreement with this composite approach. After testing several levels of theory, it was found that PBE0/BS1 and PBE0/BS3 computed Δ*E_e_*^‡^ values do indeed qualitatively correlate with ccCA-TM. Consequently, PBE0/BS1 was extended to the larger, less symmetric (TMCOT)*M*(CO)_3_ systems. Notably, ccCA-TM relative energetics computed using the weighted and non-weighted double-ζ basis sets for CCSD(T) and CCSD(T, FC1) single points were isoenergetic within 0.1 kcal mol^−1^.

The Δ*E_e_*^‡^ values for (COT)Cr(CO)_3_ computed using the PBE0/BS1 and PBE0/BS3 levels of theory were both in agreement with the relative ordering presented by ccCA-TM. However, PBEPBE energetics computed with BS1, BS2, and BS3 each showed that the 1,2 and 1,3-shifts were nearly isoenergetic with an energy difference of 0.2 kcal mol^−1^. B3LYP electronic energetics computed with BS1, BS3, and BS5 each showed that the 1,3-shift was the lowest energy structure, where the 1,2-shift was lower in energy than the CO-rotation. The electronic energies computed at the B97-1/BS2 and B97-1/BS3 levels of theory depicted that the 1,3-shift was the lowest energy process, followed by the CO-rotation and the 1,2-shift. This data is provided in Table S3.

It is evident that for (COT)Cr(CO)_3_, the 1,3-shift is indeed the lowest energy fluxional process based on experimental and computational results. The experimental Δ*G*^‡^ value for the 1,3-shift of (COT)Cr(CO)_3_ was reported to be 15.2 kcal mol^−1^, which is 1.7 kcal mol^−1^ higher than the PBE0/BS1 computed Δ*G*^‡^ (Δ*G*^‡^_comp_) for **TS-2** (13.5 kcal mol^−1^). There are structural similarities in the transition states of the fluxional processes of each (TMCOT)*M*(CO)_3_ complex. Cotton and coworkers found that 1,2-shift-a (**TS-B**) is the first process that causes coalescence in (TMCOT)Cr(CO)_3_ with a Δ*G*^‡^ of 16.0 kcal mol^−1^ (Δ*G*^‡^_comp_ = 12.6 kcal mol^−1^ using PBE0/BS1). The experimental activation energy for **TS-B** for the Mo derivative is 16.0 kcal mol^−1^ (Δ*G*^‡^_comp_ = 12.8 kcal mol^−1^ using PBE0/BS1, see Table 1), while the experimental activation energy for the W complex is 19.3 kcal mol^−1^ [30] (Δ*G*^‡^_comp_ = 13.2 kcal mol^−1^ using PBE0/BS1, see Table 1).

Solvation of (TMCOT)*M*(CO)_3_ in chloroform SMD raises the Δ*G*^‡^ of most transition states, and slightly alters the relative ordering of the fluxional processes of each (TMCOT)*M*(CO)_3_ complex (Table S7).

**Figure 5:**
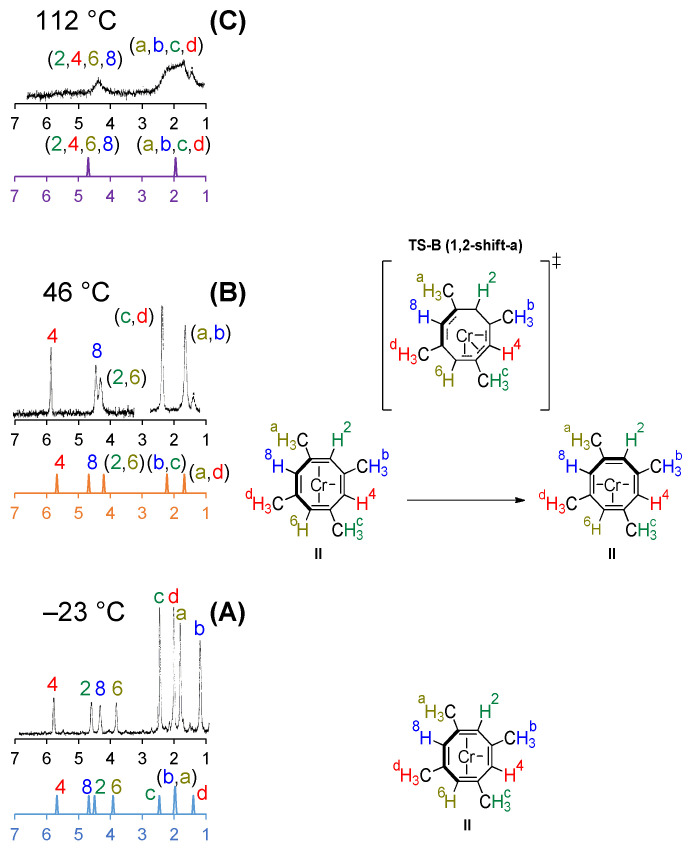
Simulated gas-phase (colored, GIAO-PBE0/BS6//PBE0/BS1) and experimental ^1^H-NMR for (TMCOT)Cr(CO)_3_ in three temperature regimes (**A**–**C**) with corresponding fluxional processes. (CO)_3_ omitted for clarity. All chemical shifts are relative to tetramethylsilane (TMS). The experimental ^1^H-NMR spectra (black) have been adapted in part with permission from Cotton, F. A.; Faller, J. W.; Musco, A., Stereochemically nonrigid organometallic molecules. XII. Temperature dependence of the proton nuclear magnetic resonance spectra of the 1,3,5,7-tetramethylcyclooctatetraene tricarbonyl compounds of chromium, molybdenum, and tungsten *J. Am. Chem. Soc.*, **1968**, *90* (6), 1438-1444. Copyright (1968) American Chemical Society [30].

**Figure 6:**
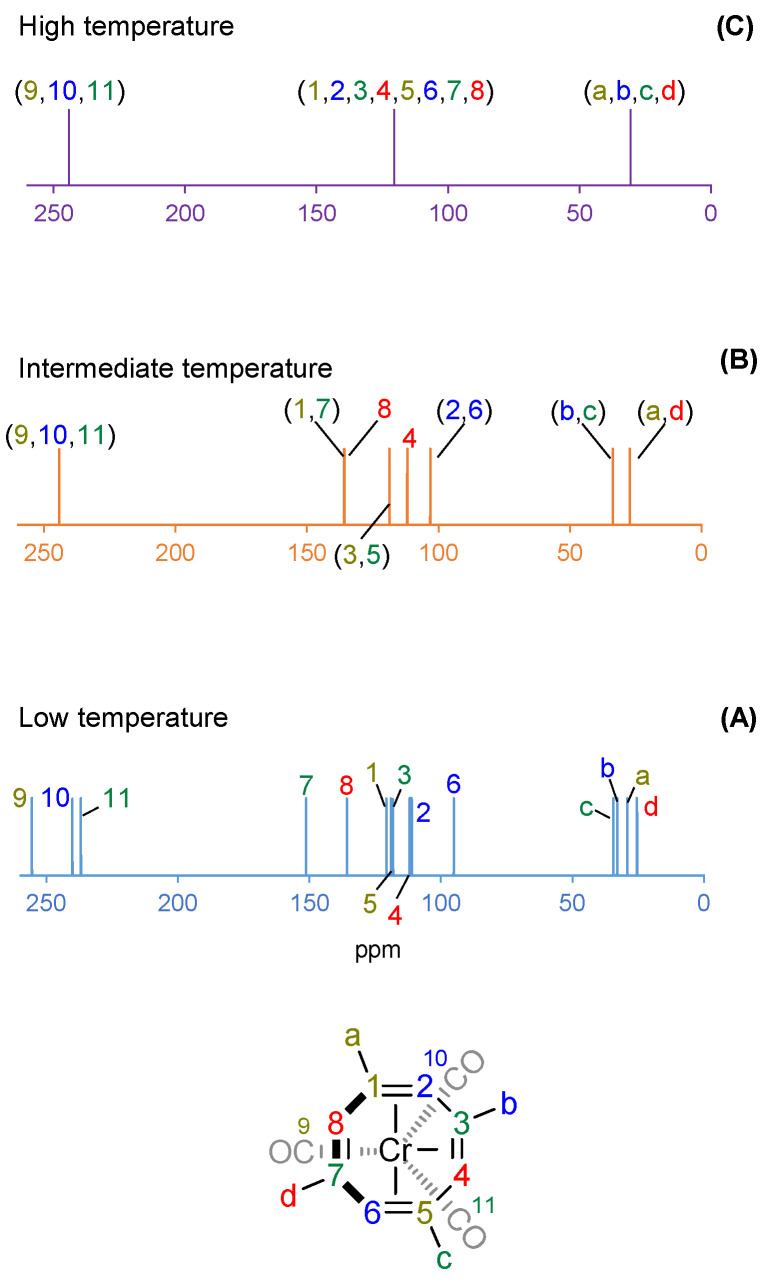
Simulated GIAO-PBE0/BS6//PBE0/BS1 ^13^C-NMR spectrum of (TMCOT)Cr(CO)_3_ at low temperature (**A**), intermediate temperature (**B**), and high temperature (**C**). Chemical shifts are relative to TMS.

Simulated ^1^H-NMR and ^13^C-NMR spectra computed at the GIAO-PBE0/BS6//PBE0/BS1 level of theory (Figure 5 and Figure 6, respectively) show that coalescence of peaks is observed when higher temperature regimes are considered. When the temperature is increased, higher energy fluxional transition states are more easily accessible for (COT)Cr(CO)_3_ and (TMCOT)*M*(CO)_3_.

The experimental and computed ^1^H-NMR spectra of (TMCOT)Cr(CO)_3_ illustrate that there are eight distinct peaks at −23 °C (Figure 5A). In the computed gas-phase ^1^H-NMR spectrum, the peaks for vinyl protons **8** and **2** have reversed assignments when compared to experimental results. Cotton and coworkers stated that there was no basis for assigning the peaks for the methyl groups in the low-temperature limit ^1^H-NMR spectrum, so the experimental labeling is arbitrary [30]. The computed assignments for the methyl peaks are **b**, **c**, **a**, **d** (downfield to upfield) while the experimental assignments were **c**, **d**, **a**, **b** (downfield to upfield) (Figure 5A).

Increasing the temperature to 46 °C leads to coalescence of equivalent vinyl protons **2**/**6** and the methyl protons represented by **b**/**c** and **a**/**d**, respectively, while vinyl protons **4** and **8** remain chemically distinct. This leads to five discernable peaks: three for the vinyl protons and two for the methyl protons (Figure 5B).

At 112 °C, the vinyl protons coalesce near 5 ppm while the methyl protons coalesced around 2 ppm (Figure 5C). Simulated gas-phase ^1^H-NMR spectra for (TMCOT)Mo(CO)_3_ resembles that of (TMCOT)Cr(CO)_3_ in each of the three temperature regimes (Figure S5).

The experimental VT ^13^C-NMR spectrum of (TMCOT)Cr(CO)_3_ shows twelve distinct peaks (^13^C-NMR chemical shifts for CO groups were not reported) [26] at low temperature while the simulated spectrum shows fifteen distinct peaks (Figure 6A). By increasing the temperature to an intermediate temperature, the peaks for carbons **4** and **8** of the TMCOT ligand remain chemically non-equivalent (Figure 6B). However, coalescence of peaks is observed in carbons **1**/**7**, **3**/**5**, **2**/**6**, **b**/**c**, and **a**/**d**, and carbons **9**/**10**/**11** (only observed computationally), thereby indicating a 1,2-shift within this temperature region. Due to the rotation rate of the three CO ligands about the Cr atom, all CO ligands are chemically equivalent. At high temperatures, coalescence is observed in each the olefin carbons, methyl carbons, and carbonyl carbons, respectively (Figure 6C).

## 4. Materials and Methods

All computations were performed using Gaussian 09 Revision D.01 [35]. All DFT computations were performed with a pruned fine integration grid with 75 radial shells and 302 angular points per shell. For all (COT)Cr(CO)_3_ complexes, full geometry optimizations and corresponding harmonic vibrational frequency computations were performed using the PBEPBE/BS1 [36,37,38], PBE0/BS1 [39], PBEPBE/BS2, B3LYP/BS1 [40,41], B3LYP/BS3, B3LYP/BS4, B3LYP/BS5, B97-1/BS2 [42,43], and B97-1/BS3 levels of theory (BS1 = 6-31G(d′) for H, C, O; mod-LANL2DZ(f)) [44,45,46] with effective core potential (ECP) for transition metals, LANL2DZ(d,p) [47,48] with ECP for Si, BS2 = 6-311++G(2df,2p) [49,50] for H, C, O; mod-LANL2TZ [45,48,51] uncontracted to [4s4p3d] for Cr, BS3 = cc-pVTZ [52,53,54,55] for all atoms, BS4 = aug-cc-pVDZ [56] for H, C, O; LANL2DZ with LANL2DZ ECP for Cr, BS5 = aug-cc-pVDZ for H, C, O; SDD [57,58] with SDD ECP for Cr). All PBEPBE/BS1 computations were performed using the density fitting procedure as implemented in Gaussian 09 [59,60,61,62].

Single point computations were performed at the HF/aug-cc-pVXZ-DK (X = D, T, Q) [56,63,64], MP2/aug-cc-pVXZ-DK (X = D, T, Q) [56,63,65,66,67,68,69,70], MP2/cc-pVTZ-DK [63,71], CCSD(T)/cc-pVXZ-DK (X = D, T) [63,72,73,74,75,76], CCSD(T)/aug-cc-pwCVDZ-DK [54], CCSD(T, FC1)/aug-cc-pVDZ-DK, and CCSD(T, FC1)/aug-cc-pwCVDZ-DK levels of theory on B3LYP/BS3 optimized geometries with Douglas–Kroll–Hess 2nd order scalar relativistic calculations [77,78,79,80,81,82] in order to derive the correlation consistent Composite Approach for transition metals (ccCA-TM) [18,19,20,21,22,23,24,25] energetics for each reported structure of (COT)Cr(CO)_3_. The total electronic energy described by ccCA-TM is represented by Equation (1).

(1) $$E_{\mathrm{total}}=E_{\mathrm{ref}}\left(\mathrm{ccCA}\right)+\Delta E\left(\mathrm{CC}\right)+\Delta E\left(\mathrm{CV}\right)+\Delta E\left(\mathrm{ZPE}\right)+\Delta E\left(\mathrm{SO}\right)$$

*E*_ref_(ccCA) represents energies at the MP2/aug-cc-pVXZ (X = D, T, Q) complete basis set (CBS) limit added to the HF/CBS energy. The HF CBS limit can be computed with a two-point extrapolation of HF energies with the aug-cc-pVTZ and aug-cc-pVQZ basis sets as seen in Equation (2).

(2) $$E\left(n\right)=E\left(\mathrm{CBS}\right)+Ae^{1.63n}$$

Equation (2) can be represented algebraically by Equation (3) [83], where B = 1.63.

(3) $$E_{\infty }=E_{X}-\left(\frac{E_{X}-E_{X+1}}{\left(1-e^{-B}\right)}\right)$$

Δ*E*(CC) is a correction that stems from CCSD(T) to account for higher order dynamic correlation effects, as it is not adequately described using MP2. Δ*E*(CV) represents a basis set correction to the core–core and core–valence electron interactions in CCSD(T). Δ*E*(ZPE) is the result of zero-point energy and thermal corrections at 298.15 K, which uses harmonic vibrational frequencies scaled by 0.989. Δ*E*(SO) is the atomic spin-orbit coupling correction [84].

For all (TMCOT)*M*(CO)_3_ complexes, full geometry optimizations and corresponding harmonic vibrational frequency computations were performed at the PBEPBE/BS1, B3LYP/BS1, and PBE0/BS1 levels of theory.

Magnetic shielding tensors for (TMCOT)Cr(CO)_3_ and (TMCOT)Mo(CO)_3_ were computed using the Gauge-Independent Atomic Orbital (GIAO) [85,86,87,88] method at the GIAO-PBE0/BS6//PBE0/BS1 level of theory. BS6 is described as the LANL08(f) [46,89] basis sets and corresponding ECP for Cr and Mo, LANL08(d) [48,89] with LANL2DZ ECP for Si, and the IGLO-ΙΙ basis sets for H, C, and O [90]. The gas-phase computed chemical shift is represented by the difference between the absolute isotropic shielding constant for the reference atom (as computed in TMS) and the absolute isotropic shielding constant for the considered atom within the complex.

Simulated ^1^H-NMR and ^13^C-NMR spectra were obtained using an in-house Fortran program by convoluting the computed absolute isotropic shielding values and relative chemical shift with a Gaussian line shape and broadening of 0.025 ppm [91]. To account for peak averaging, the relative isotropic shielding values for chemically equivalent protons and carbons were considered (e.g., the relative isotropic shielding values for the three protons on a methyl group were considered, and the resulting peak was given at the mean chemical shift of these three peaks). To account for temperature-based peak averaging, the chemical shifts of protons and carbons that become chemically equivalent through fluxional processes at each temperature regime were averaged, thereby resulting in coalesced peaks.

Solvation effects on the fluxional processes have been considered by the using the SMD (chloroform) implicit solvation model via self-consistent reaction field (SCRF). Single-point solvation energy computations were performed on gas-phase optimized structures at the SMD-PBE0//PBE0/BS1 level of theory [92].

## 5. Conclusions

Density functional theory (DFT) was applied to investigate the fluxional processes in (COT)Cr(CO)_3_ and (TMCOT)*M*(CO)_3_ (*M* = Cr, Mo, and W). ccCA-TM energetics demonstrated that **TS-2** (1,3-shift) is the lowest-energy fluxional process of (COT)Cr(CO)_3_ (Δ*E_e_*^‡^ = 17.2 kcal mol^−1^). It was also discovered that the 1,2-shift represented by **TS-B** (also known as 1,2-shift-a) is the lowest-energy fluxional process for all three (TMCOT)*M*(CO)_3_ complexes (Δ*G*^‡^ = 12.6 kcal mol^−1^, 12.8 kcal mol^−1^, and 13.2 kcal mol^−1^ for *M* = Cr, Mo, and W, respectively), which was reaffirmed by the analysis of the experimental and computed ^1^H and ^13^C-NMR chemical shifts. The computed free energy of activation for **TS-B** of each of these complexes is consistently slightly lower than the reported experimental results. Implicit solvation slightly alters the relative energies and ordering for the fluxional transition states of all (TMCOT)*M*(CO)_3_ complexes. By increasing the temperature to the 112 °C, coalescence of the vinyl hydrogen and methyl peaks, respectively, is observed in the ^1^H-NMR spectrum of (TMCOT)Cr(CO)_3_. Similarly, methyl, olefin, and carbonyl carbon peaks coalesce in the high temperature region of the ^13^C-NMR spectrum of (TMCOT)Cr(CO)_3_.

## Figures and Tables

**Table 1:** Computed relative electronic energies and free energies (in parentheses) for (TMCOT)*M*(CO)_3_ (*M* = Cr, Mo, W) fluxional transition states with DFT/BS1 (all energies reported in kcal mol^−1^).

		TS-A (CO-rot)	TS-B (1,2-a)	TS-C (1,3)	TS-D (1,5)	TS-E (1,2-b)	TS-F (1,4)
***M*** **= Cr**							
	**PBEPBE**	11.8 (12.0)	10.2 (9.7)	16.7 (16.6)	22.3 (20.6)	20.0 (17.3)	42.8 (41.8)
	**B3LYP**	13.4 (13.8)	10.5 (9.9)	13.7 (13.9)	16.4 (15.0)	20.9 (18.1)	46.8 (44.8)
	**PBE0**	13.0 (13.4)	13.6 (12.6)	18.1 (18.0)	23.1 (21.3)	24.9 (21.9)	49.1 (47.9)
***M*** **= Mo**							
	**PBEPBE**	12.0 (12.7)	10.1 (9.7)	15.3 (15.9)	19.0 (18.4)	19.9 (18.3)	41.4 (40.8)
	**B3LYP**	14.0 (14.5)	10.6 (9.9)	13.7 (14.3)	15.5 (15.0)	21.2 (18.8)	44.5 (43.7)
	**PBE0**	13.3 (13.8)	13.6 (12.8)	17.5 (18.0)	21.1 (20.3)	24.7 (22.5)	47.0 (46.3)
***M*** **= W**							
	**PBEPBE**	10.2 (10.8)	10.7 (10.1)	19.3 (19.7)	24.7 (23.2)	21.2 (19.7)	40.9 (40.1)
	**B3LYP**	11.6 (12.3)	10.4 (9.9)	17.2 (17.8)	21.7 (21.0)	21.5 (19.8)	42.2 (41.6)
	**PBE0**	11.1 (11.6)	14.0 (13.2)	22.1 (22.3)	28.2 (26.9)	25.7 (23.8)	45.8 (45.1)

## Data Availability

The data presented in this study are available in this article.

## Supplementary Materials

The following are available online, Table S1: Computed bond lengths from Cr to COT C atoms (in Å), Table S2: Δ*G*/Δ*G*^‡^ values for fluxional transition states of (COT)Cr(CO)_3_ (in kcal mol^−1^), Table S3: Δ*E_e_*/Δ*E_e_*^‡^ values for fluxional transition states of (COT)Cr(CO)_3_ (in kcal mol^−1^), Table S4: Raw Energies Utilized in Assessment of ccCA-TM Energies Using P Extrapolation (in a.u.), Table S5: ΔΔ*E_e_*/ΔΔ*E_e_*^‡^ values for fluxional transition states of (COT)Cr(CO)_3_ and statistical parameters (R^2^ = least squared regression, m = slope, b = y-intercept) in kcal mol^−1^ (relative to ccCA-TM), Figure S1: Plot of Δ*E_e_*_,DFT_ vs. Δ*E_e_*_,ccCA-TM_ (in kcal mol^−1^) for (COT)Cr(CO)_3_ computed at each level of theory. Table S6: Experimental and computed bond lengths and angles for (TMCOT)Cr(CO)_3_; computed bond lengths and angles for (TMCOT)Mo(CO)_3_ and (TMCOT)W(CO)_3_ (PBE0/BS1 level of theory). (M = Cr, Mo, W), Figure S2: Top-down view of the reported crystal structure of (TMCOT)Cr(CO)_3_ with atom labels. Hydrogens omitted for clarity, Figure S3: 2D and 3D representation of **TS-G**, Figure S4: 2D and 3D representation of **TS-6** and **2**. Table S7: Δ*G*^‡^_solution_ of (TMCOT)*M*(CO)_3_ complexes with implicit chloroform solvation model at the SMD-PBE0//PBE0/BS1 level of theory (gas phase Δ*G*^‡^ in parentheses). All relative free energies reported in kcal mol^−1^. Figure S5: Simulated (colored) gas-phase and experimental (black) ^1^H-NMR for the lowest energy structure of (TMCOT)Mo(CO)_3_ at the GIAO-PBEPBE/BS6//PBEPBE/BS1 level of theory. Results given for low-temperature limit (**A**, −10° C), low temperature (**B**, 40 °C), and high-temperature limit (**C**, >80 °C). Experimental ^1^H-NMR for high temperature limit not reported due to thermal decomposition of (TMCOT)Mo(CO)_3_ above 80° C. Experimental spectra adapted in part with permission from Cotton, F. A.; Faller, J. W.; Musco, A. Stereochemically Nonrigid Organometallic Compounds. II. 1,3,5,7-Tetramethylcyclo-Octatetraenemolybdenum Tricarbonyl. *J. Am. Chem. Soc.* **1966**, *88* (19), 4506–4507. Copyright (1966) American Chemical Society. Figure S6: Simulated gas-phase (colored, GIAO-PBEPBE/BS6//PBEPBE/auto/BS1) and experimental ^1^H-NMR for (TMCOT)Cr(CO)_3_ in three temperature regimes (**A**–**C**) with corresponding fluxional processes. (CO)_3_ omitted for clarity. All chemical shifts are relative to tetramethylsilane (TMS). Experimental spectra adapted in part with permission from Cotton, F. A.; Faller, J. W.; Musco, A. Stereochemically nonrigid organometallic molecules. XII. Temperature dependence of the proton nuclear magnetic resonance spectra of the 1,3,5,7-tetramethylcyclooctatetraene tricarbonyl compounds of chromium, molybdenum, and tungsten *J. Am. Chem. Soc.*, **1968**, *90* (6), 1438–1444. Copyright (1968) American Chemical Society. Table S8: Experimental and computed ^1^H-NMR chemical shifts in (TMCOT)Cr(CO)_3_ and (TMCOT)Mo(CO)_3_ at the GIAO-PBEPBE/BS6//PBEPBE/auto/BS1 level of theory (measured in ppm; relative to TMS). Figure S7: 2D-representation of (TMCOT)M(CO)_3_ (M = Cr, Mo) with hydrogens labeled. (CO)_3_ omitted for clarity. Table S9: Computed chemical shifts for ^13^C-NMR in (TMCOT)Cr(CO)_3_ at the GIAO-PBEPBE/BS6//PBEPBE/auto/BS1 (relative to TMS). See Figure S2 for appropriate atom labeling. Table S10: Basis Sets Used in Study, Molecular Coordinates for (COT)Cr(CO)_3_ (Å), Molecular Coordinates for (TMCOT)Cr(CO)_3_ (Å), Molecular Coordinates for (TMCOT)Mo(CO)_3_ (Å), and Molecular Coordinates for (TMCOT)W(CO)_3_ (Å).
