# Supplementary material for: Benchmarking the Fluxional Processes of Organometallic Piano-Stool Complexes
Source: Molecules. 2021 Apr 16;26(8):2310. doi: 10.3390/molecules26082310 (PMC8073612; doi:10.3390/molecules26082310)
Supplement: Supplementary file 1 [file molecules-26-02310-s001.zip › molecules-1189280-supplementary.pdf]

Supporting Information

Benchmarking the Fluxional Processes of Organometallic Piano Stool Complexes

Nathan C. Frey, Eric Van Dornshuld, Charles Edwin Webster*

Department of Chemistry, Mississippi State University, Mississippi State, Mississippi 39762, United States

Contents

Table S1. Computed bond lengths from Cr to COT C atoms (in Å). 3

Table S2. $\Delta G/\Delta G^{\ddagger}$ values for fluxional transition states of (COT)Cr$(CO)_3$ (in kcal mol^{-1}).5

Table S3. $\Delta E_e/\Delta E_e^{\ddagger}$ values for fluxional transition states of (COT)Cr$(CO)_3$ (in kcal mol^{-1}). 5

Table S4. Raw Energies Utilized in Assessment of ccCA-TM Energies Using P Extrapolation (in a.u.). 6

Table S5. $\Delta\Delta E_e/\Delta\Delta E_e^{\ddagger}$ values for fluxional transition states of (COT)Cr$(CO)_3$ and statistical parameters (R^2 = least squared regression, m = slope, b = y-intercept) in kcal mol^{-1} (relative to ccCA-TM). 7

Figure S1. Plot of $\Delta E_{e,DFT}$ vs. $\Delta E_{e,ccCA\text{-}TM}$ (in kcal mol^{-1}) for (COT)Cr$(CO)_3$ computed at each level of theory. 8

Table S6. Experimental and computed bond lengths and angles for (TMCOT)Cr$(CO)_3$; computed bond lengths and angles for (TMCOT)Mo$(CO)_3$ and (TMCOT)W$(CO)_3$ (PBE0/BS1 level of theory). (M = Cr, Mo, W) 9

Figure S2. Top-down view of the reported crystal structure of (TMCOT)Cr$(CO)_3$ with atom labels. Hydrogens omitted for clarity.[2] 9

Figure S3. 2D and 3D representation of **TS-G** 10

Figure S4. 2D and 3D representation of **TS-6** and **2**. 10

Table S7. $\Delta G^{\ddagger}_{solution}$ of (TMCOT)$M(CO)_3$ complexes with implicit chloroform solvation model at the SMD-PBE0//PBE0/BS1 level of theory (gas phase $\Delta G^{\ddagger}$ in parentheses). All relative free energies reported in kcal mol^{-1}. 10

Figure S5. Simulated (colored) gas-phase and experimental (black) ^{1}H-NMR for the lowest energy structure of (TMCOT)Mo$(CO)_3$ at the GIAO-PBEPBE/BS6//PBEPBE/BS1 level of theory. Results given for low-temperature limit (**A**, –10° C), low temperature (**B**, 40° C), and high-temperature limit (**C**, >80° C). Experimental ^{1}H-NMR for high temperature limit not reported due to thermal decomposition of (TMCOT)Mo$(CO)_3$ above 80° C. Experimental spectra adapted in part with permission from Cotton, F. A.; Faller, J. W.; Musco, A. Stereochemically Nonrigid Organometallic Compounds. II. 1,3,5,7-Tetramethylcyclo-Octatetraenemolybdenum Tricarbonyl. *J. Am. Chem. Soc.* **1966**, *88* (19), 4506–4507. Copyright (1966) American Chemical Society. 11

Figure S6. Simulated gas-phase (colored, GIAO-PBEPBE/BS6//PBEPBE/auto/BS1) and experimental ^{1}H-NMR for (TMCOT)Cr$(CO)_3$ in three temperature regimes (A, B, C) with corresponding fluxional processes. $(CO)_3$ omitted for clarity. All chemical shifts are relative to tetramethylsilane (TMS). Experimental spectra adapted in part with permission from Cotton, F. A.; Faller, J. W.; Musco, A., Stereochemically nonrigid organometallic molecules. XII. Temperature dependence of the proton nuclear magnetic resonance spectra of the 1,3,5,7-tetramethylcyclooctatetraene tricarbonyl compounds of chromium, molybdenum, and tungsten *J. Am. Chem. Soc.*, **1968**, *90* (6), 1438-1444. Copyright (1968) American Chemical Society. 12

Table S8. Experimental and computed ^{1}H-NMR chemical shifts in (TMCOT)Cr$(CO)_3$ and (TMCOT)Mo$(CO)_3$ at the GIAO-PBEPBE/BS6//PBEPBE/auto/BS1 level of theory (measured in ppm; relative to TMS). 13

Figure S7. 2D-representation of (TMCOT)M$(CO)_3$ (M = Cr, Mo) with hydrogens labelled. $(CO)_3$ omitted for clarity. 13

Table S9. Computed chemical shifts for ^{13}C-NMR in (TMCOT)Cr$(CO)_3$ at the GIAO-PBEPBE/BS6//PBEPBE/auto/BS1 (relative to TMS). See Figure S1 for appropriate atom labelling 14

Table S10. Basis Sets Used in Study 14

Molecular Coordinates for (COT)Cr$(CO)_3$ (Å) 15

Molecular Coordinates for (TMCOT)Cr$(CO)_3$ (Å) 33

Molecular Coordinates for (TMCOT)Mo$(CO)_3$ (Å) 41

Molecular Coordinates for (TMCOT)W$(CO)_3$ (Å) 49

References 58

Table S1. Computed bond lengths from Cr to COT C atoms (in Å).

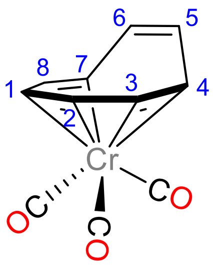

I (minimum)	Level of Theory	C_1	C_2	C_3	C_4	C_5	C_6	C_7	C_8
	PBE0/BS1	2.211	2.211	2.176	2.305	–	–	2.305	2.176
	PBE0/BS3	2.208	2.208	2.177	2.309	–	–	2.309	2.177
	PBEPBE/BS1	2.234	2.234	2.197	2.342	–	–	2.342	2.197
	PBEPBE/BS2	2.231	2.231	2.198	2.343	–	–	2.343	2.198
	PBEPBE/BS3	2.230	2.230	2.196	2.339	–	–	2.339	2.196
	B3LYP/BS1	2.260	2.260	2.235	2.410	–	–	2.410	2.235
	B3LYP/BS3	2.254	2.254	2.235	2.416	–	–	2.416	2.235
	B3LYP/BS4	2.260	2.260	2.235	2.408	–	–	2.408	2.235
	B3LYP/BS5	2.259	2.259	2.234	2.407	–	–	2.407	2.234
	B97-1/BS2	2.249	2.249	2.224	2.384	–	–	2.384	2.224
	B97-1/BS3	2.242	2.242	2.215	2.369	–	–	2.369	2.215
**TS-1 (CO Rot.)**	**Level of Theory**	**C_1**	**C_2**	**C_3**	**C_4**	**C_5**	**C_6**	**C_7**	**C_8**
	PBE0/BS1	2.213	2.213	2.187	2.265	–	–	2.265	2.187
	PBE0/BS3	2.209	2.209	2.186	2.264	–	–	2.264	2.186
	PBEPBE/BS1	2.237	2.237	2.211	2.303	–	–	2.303	2.211
	PBEPBE/BS2	2.232	2.232	2.208	2.301	–	–	2.301	2.208
	PBEPBE/BS3	2.230	2.230	2.196	2.339	–	–	2.339	2.196
	B3LYP/BS1	2.268	2.268	2.244	2.342	–	–	2.342	2.244
	B3LYP/BS3	2.262	2.262	2.240	2.338	–	–	2.338	2.240
	B3LYP/BS4	2.269	2.269	2.242	2.337	–	–	2.337	2.242
	B3LYP/BS5	2.267	2.267	2.242	2.336	–	–	2.336	2.242
	B97-1/BS2	2.253	2.253	2.229	2.312	–	–	2.312	2.229
	B97-1/BS3	2.244	2.244	2.220	2.301	–	–	2.301	2.220
**TS-2 (1,3)**	**Level of Theory**	**C_1**	**C_2**	**C_3**	**C_4**	**C_5**	**C_6**	**C_7**	**C_8**
	PBE0/BS1	2.145	2.145	2.219	–	–	–	–	2.219
	PBE0/BS3	2.147	2.147	2.228	–	–	–	–	2.228
	PBEPBE/BS1	2.167	2.167	2.263	–	–	–	–	2.263
	PBEPBE/BS2	2.167	2.167	2.265	–	–	–	–	2.265
	PBEPBE/BS3	2.168	2.168	2.268	–	–	–	–	2.268
	B3LYP/BS1	2.185	2.185	2.285	–	–	–	–	2.285
	B3LYP/BS3	2.184	2.184	2.290	–	–	–	–	2.290
	B3LYP/BS4	2.186	2.186	2.279	–	–	–	–	2.279

	**B3LYP/BS5**	2.183	2.183	2.278	–	–	–	–	2.278
	**B97-1/BS2**	2.179	2.179	2.269	–	–	–	–	2.269
	**B97-1/BS3**	2.174	2.174	2.265	–	–	–	–	2.265
**TS-3 (1,2)**	**Level of Theory**	**C_1**	**C_2**	**C_3**	**C_4**	**C_5**	**C_6**	**C_7**	**C_8**
	**PBE0/BS1**	2.173	2.128	2.367	–	–	–	2.367	2.128
	**PBE0/BS3**	2.173	2.140	2.380	–	–	–	2.380	2.140
	**PBEPBE/BS1**	2.192	2.158	2.409	–	–	–	2.409	2.158
	**PBEPBE/BS2**	2.193	2.166	2.411	–	–	–	2.411	2.166
	**PBEPBE/BS3**	2.193	2.167	2.413	–	–	–	2.413	2.167
	**B3LYP/BS1**	2.206	2.176	2.442	–	–	–	2.442	2.176
	**B3LYP/BS3**	2.204	2.185	2.453	–	–	–	2.453	2.185
	**B3LYP/BS4**	2.207	2.180	2.440	–	–	–	2.440	2.180
	**B3LYP/BS5**	2.207	2.179	2.438	–	–	–	2.438	2.179
	**B97-1/BS2**	2.203	2.174	2.429	–	–	–	2.429	2.174
	**B97-1/BS3**	2.197	2.169	2.427	–	–	–	2.427	2.169
**TS-4 (1,5)**	**Level of Theory**	**C_1**	**C_2**	**C_3**	**C_4**	**C_5**	**C_6**	**C_7**	**C_8**
	**PBE0/BS1**	2.228	2.228	–	–	2.620	2.620	–	–
	**PBE0/BS3**	2.236	2.236	–	–	2.609	2.609	–	–
	**PBEPBE/BS1**	2.254	2.254	–	–	2.630	2.630	–	–
	**PBEPBE/BS2**	2.263	2.263	–	–	2.611	2.611	–	–
	**PBEPBE/BS3**	2.264	2.264	–	–	2.606	2.606	–	–
	**B3LYP/BS1**	2.288	2.288	–	–	2.706	2.706	–	–
	**B3LYP/BS3**	2.297	2.297	–	–	2.707	2.707	–	–
	**B3LYP/BS4**	2.295	2.295	–	–	2.697	2.697	–	–
	**B3LYP/BS5**	2.294	2.294	–	–	2.695	2.695	–	–
	**B97-1/BS2**	2.272	2.272	–	–	2.681	2.681	–	–
	**B97-1/BS3**	2.270	2.270	–	–	2.677	2.677	–	–
**TS-5 (1,4)**	**Level of Theory**	**C_1**	**C_2**	**C_3**	**C_4**	**C_5**	**C_6**	**C_7**	**C_8**
	**PBE0/BS1**	2.167	2.259	–	–	2.164	–	–	2.259
	**PBE0/BS3**	2.175	2.272	–	–	2.175	–	–	2.272
	**PBEPBE/BS1**	2.193	2.291	–	–	2.212	–	–	2.292
	**PBEPBE/BS2**	2.198	2.300	–	–	2.231	–	–	2.300
	**PBEPBE/BS3**	2.199	2.302	–	–	2.233	–	–	2.302
	**B3LYP/BS1**	2.216	2.321	–	–	2.215	–	–	2.321
	**B3LYP/BS3**	2.221	2.331	–	–	2.228	–	–	2.331
	**B3LYP/BS4**	2.218	2.326	–	–	2.217	–	–	2.326
	**B3LYP/BS5**	2.215	2.326	–	–	2.220	–	–	2.325
	**B97-1/BS2**	2.214	2.319	–	–	2.210	–	–	2.319
	**B97-1/BS3**	2.207	2.310	–	–	2.204	–	–	2.310

Table S2. $\Delta G/\Delta G^{\ddagger}$ values for fluxional transition states of (COT)Cr(CO)$_3$ (in kcal mol^{-1}).

	PBE0/ BS1	PBE0/ BS3	PBEPBE/ BS1	PBEPBE/ BS2	PBEPBE/ BS3	B3LYP/ BS1	B3LYP/ BS3	B3LYP/ BS4	B3LYP/ BS5	B97-1/ BS2	B97-1/ BS3
**I (minimum)**	0.0	0.0	0.0	0.0	0.0	0.0	0.0	0.0	0.0	0.0	0.0
**TS-1 (CO-rot)**	12.9	12.3	11.8	11.5	11.3	13.7	13.4	13.4	13.2	13.0	12.7
**TS-2 (1,3)**	13.5	13.1	12.3	12.3	12.4	9.1	8.5	9.1	8.8	9.7	10.3
**TS-3 (1,2)**	14.8	14.3	11.1	11.2	11.1	10.7	10.0	10.4	10.0	11.4	11.6
**TS-4 (1,5)**	23.3	24.2	22.2	22.6	23.5	16.9	17.7	17.0	17.1	18.6	20.5
**TS-5 (1,4)**	47.4	46.5	40.8	40.7	40.6	44.5	43.6	43.7	43.0	44.3	44.2

Table S3. $\Delta E_e/\Delta E_e^{\ddagger}$ values for fluxional transition states of (COT)Cr(CO)$_3$ (in kcal mol^{-1}).

	PBE0/ BS1	PBE0/ BS3	PBEPBE/ BS1	PBEPBE/ BS2	PBEPBE/ BS3	B3LYP/ BS1	B3LYP/ BS3	B3LYP/ BS4	B3LYP/ BS5	B97-1/ BS2	B97-1/ BS3	ccCA-TM
**I (minimum)**	0.0	0.0	0.0	0.0	0.0	0.0	0.0	0.0	0.0	0.0	0.0	0.0
**TS-1 (CO-rot)**	12.6	12.0	11.6	11.0	11.2	13.3	12.9	13.0	12.7	12.5	12.2	11.0
**TS-2 (1,3)**	14.2	13.9	12.8	13.1	13.0	9.4	8.9	9.4	9.2	10.1	10.8	17.0
**TS-3 (1,2)**	16.8	16.3	12.7	12.9	12.9	12.3	11.6	12.0	11.7	13.2	13.5	18.8
**TS-4 (1,5)**	25.6	26.7	24.3	25.7	24.8	18.6	19.4	18.9	19.0	20.6	21.5	29.2
**TS-5 (1,4)**	48.9	47.9	42.7	42.6	42.8	46.5	45.5	45.6	44.9	46.1	45.9	47.4

Table S4. Raw Energies Utilized in Assessment of ccCA-TM Energies Using P Extrapolation (in a.u.).

	I (minimum)	TS-1 (CO-rot)	TS-2 (1,3)	TS-3 (1,2)	TS-4 (1,5)	TS-5 (1,4)
**HF/aug-cc-pVTZ-DK**	-1695.768876	-1695.746516	-1695.766307	-1695.752945	-1695.747831	-1695.704804
**HF/aug-cc-pVQZ-DK**	-1695.808584	-1695.786219	-1695.806079	-1695.792743	-1695.787631	-1695.744553
**HF/CBS-DK**	-1695.81826	-1695.795893	-1695.81577	-1695.80244	-1695.797329	-1695.754239
**MP2/aug-cc-pVDZ-DK**	-1698.109075	-1698.076155	-1698.056546	-1698.049605	-1698.071521	-1697.963276
**MP2/aug-cc-pVTZ-DK**	-1698.672971	-1698.64005	-1698.621527	-1698.613677	-1698.635623	-1698.527119
**MP2/aug-cc-pVQZ-DK**	-1698.859862	-1698.826771	-1698.808645	-1698.800487	-1698.823193	-1698.713826
**MP2/CBS-DK + HF/CBS-DK (P)**	-3.136330259	-3.125477818	-3.087719343	-3.092691233	-3.121012074	-3.05416263
**MP2/cc-pVTZ-DK**	-1698.61531	-1698.582505	-1698.565085	-1698.557464	-1698.577866	-1698.471052
**CCSD(T)/cc-pVTZ-DK**	-1698.722693	-1698.7046	-1698.698031	-1698.693904	-1698.675409	-1698.647357
**CCSD(T)/aug-cc-pCVDZ-DK**	-1698.259687	-1698.242006	-1698.232798	-1698.229627	-1698.211603	-1698.184003
**CCSD(T,FC1)/aug-cc-pCVDZ-DK**	-1699.123923	-1699.106407	-1699.095199	-1699.092212	-1699.073703	-1699.047402
**D(CC)**	-0.1073827	-0.1220958	-0.1329461	-0.1364394	-0.0975427	-0.1763049
**D(CV)**	-0.8642364	-0.8644015	-0.8624008	-0.8625851	-0.8620994	-0.8633987
**ccCA-TM E_e (P)**	-1699.926209	-1699.907868	-1699.898837	-1699.894156	-1699.877983	-1699.848105
**scaled ZPE**	0.159248635	0.158487913	0.15893827	0.157185185	0.157532442	0.156622985
**ccCA-TM E_{ZPE} (P)**	-1699.766961	-1699.74938	-1699.739898	-1699.736971	-1699.72045	-1699.691482

Table S5. $\Delta\Delta E_e/\Delta\Delta E_e^{\ddagger}$ values for fluxional transition states of $(COT)Cr(CO)_3$ and statistical parameters (R^2 = least squared regression, m = slope, b = y-intercept) in kcal mol^{-1} (relative to ccCA-TM).

	PBE0/ BS1	PBE0/ BS3	PBEPBE/ BS1	PBEPBE/ BS2	PBEPBE/ BS3	B3LYP/ BS1	B3LYP/ BS3	B3LYP/ BS4	B3LYP/ BS5	B97-1/ BS2	B97-1/ BS3
**R^2**	0.982	0.988	0.979	0.984	0.983	0.904	0.913	0.911	0.915	0.937	0.950
**m**	1.006 ± 0.069	0.998 ± 0.055	0.888 ± 0.065	0.898 ± 0.057	0.895 ± 0.059	0.925 ± 0.151	0.915 ± 0.142	0.912 ± 0.142	0.901 ± 0.137	0.933 ± 0.121	0.935 ± 0.107
**b**	–0.998 ± 1.739	–1.065 ± 1.398	–0.913 ± 1.657	–0.926 ± 1.438	–0.965 ± 1.502	–2.345 ± 3.827	–2.442 ± 3.592	–2.263 ± 3.604	–2.282 ± 3.480	–2.102 ± 3.080	–1.904 ± 2.710
**I (minimum)**	0.0	0.0	0.0	0.0	0.0	0.0	0.0	0.0	0.0	0.0	0.0
**TS-1 (CO-rot)**	1.6	0.9	0.5	–0.1	0.2	2.3	1.9	2.0	1.7	1.4	1.1
**TS-2 (1,3)**	–2.8	–3.1	–4.2	–3.9	–4.0	–7.6	–8.1	–7.6	–7.8	–6.8	–6.2
**TS-3 (1,2)**	–2.0	–2.5	–6.1	–5.9	–5.9	–6.5	–7.2	–6.8	–7.1	–5.6	–5.3
**TS-4 (1,5)**	–3.6	–2.6	–5.0	–3.6	–4.4	–10.7	–9.8	–10.3	–10.3	–8.6	–7.7
**TS-5 (1,4)**	1.6	0.6	–4.6	–4.7	–4.6	–0.9	–1.9	–1.8	–2.4	–1.3	–1.5

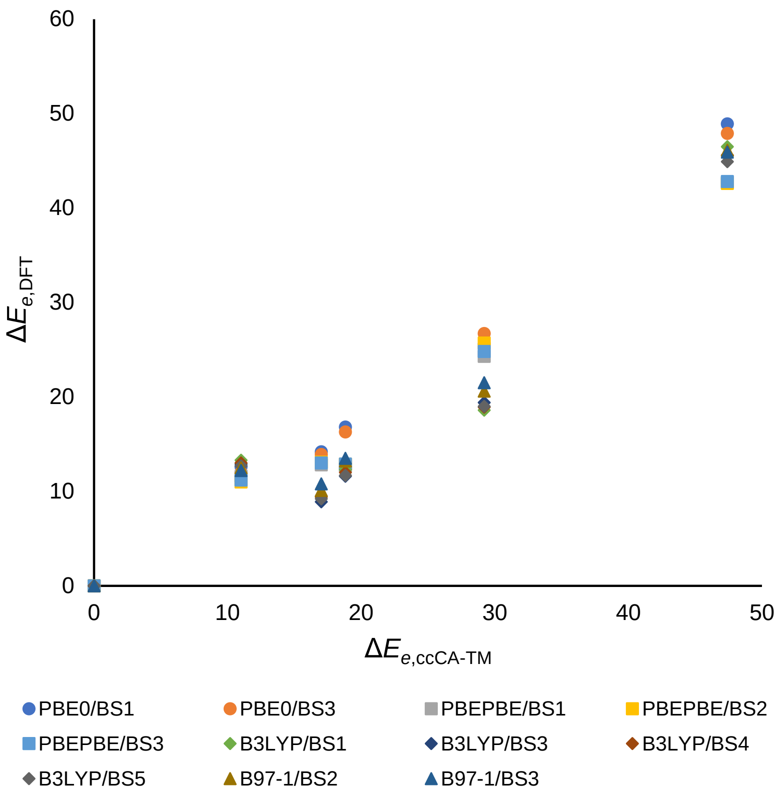

Figure S1. Plot of $\Delta E_{e,\mathrm{DFT}}$ vs. $\Delta E_{e,\mathrm{ccCA\text{-}TM}}$ (in kcal mol^{-1}) for (COT)Cr$(CO)_3$ computed at each level of theory.

Table S4. Experimental and computed bond lengths and angles for (TMCOT)Cr$(CO)_3$; computed bond lengths and angles for (TMCOT)Mo$(CO)_3$ and (TMCOT)W$(CO)_3$ (PBE0/BS1 level of theory). (M = Cr, Mo, W)

	**(TMCOT)Cr(CO)$_3$**		**(TMCOT)Mo(CO)$_3$**	**(TMCOT)W(CO)$_3$**
Bond length (Å)	Expt	Comp	Comp	Comp
M–C2	2.20	2.18	2.31	2.29
M–C3	2.29	2.25	2.39	2.36
M–C12	2.41	2.39	2.50	2.45
M–C13	2.21	2.18	2.31	2.29
M–C14	2.25	2.23	2.37	2.35
M–C15	2.23	2.22	2.35	2.33
M–C1	1.85	1.84	1.99	1.99
M–C8	1.86	1.83	1.99	1.99
M–C9	1.84	1.83	1.99	1.99
Bond angle (°)	Expt	Comp	Comp	Comp
C10–C11–C12	124.4	123.2	124.9	123.7
C11–C10–C3	121.0	120.0	121.4	120.1
C10–C3–C2	124.2	123.2	123.8	122.7
C3–C2–C15	125.1	125.0	125.6	124.9
C2–C15–C14	134.2	133.1	133.9	133.9
C1–M1–C8	94.2	93.0	91.5	92.1
C1–M1–C9	81.5	81.8	82.5	82.1

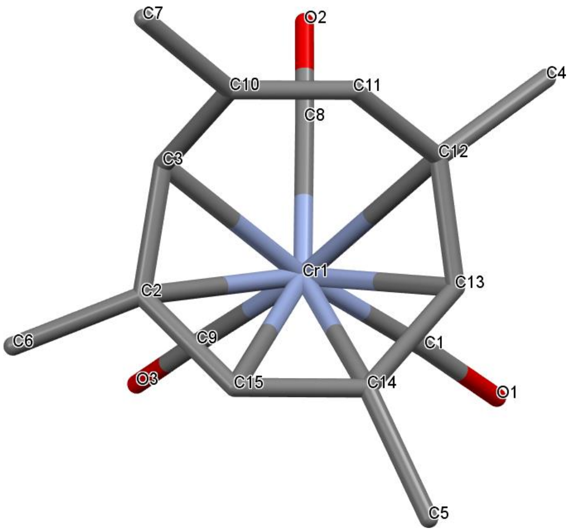

Figure S2. Top-down view of the reported crystal structure of (TMCOT)Cr$(CO)_3$ with atom labels. Hydrogens omitted for clarity.[2]

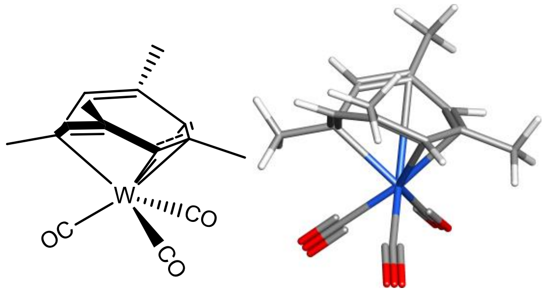

Figure S3. 2D and 3D representation of **TS-G**

TS-6

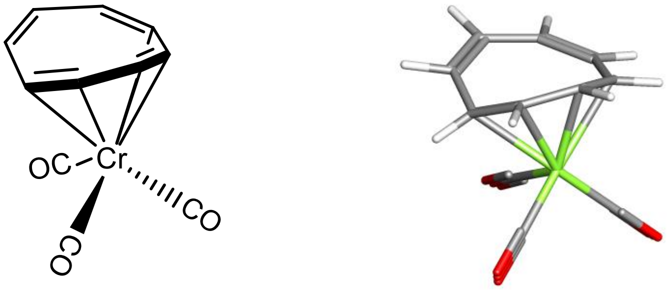

2

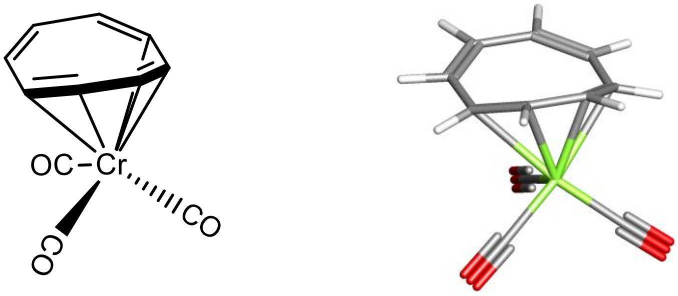

Figure S4. 2D and 3D representation of **TS-6** and **2**.

Table S7. $\Delta G^{\ddagger}_{\text{solution}}$ of (TMCOT)M(CO)$_3$ complexes with implicit chloroform solvation model at the SMD-PBE0//PBE0/BS1 level of theory (gas phase $\Delta G^{\ddagger}$ in parentheses). All relative free energies reported in kcal mol^{-1}.

**Transition State**	**(TMCOT)Cr(CO)$_3$**	**(TMCOT)Mo(CO)$_3$**	**(TMCOT)W(CO)$_3$**
**TS-A (CO-rot)**	12.8 (12.6)	13.6 (13.8)	11.7 (11.6)
**TS-B (1,2-a)**	14.0 (13.4)	13.9 (12.8)	14.5 (13.2)
**TS-C (1,3)**	18.3 (18.0)	21.1 (18.0)	27.0 (22.3)
**TS-D (1,5)**	23.2 (21.3)	25.7 (20.3)	33.2 (26.9)
**TS-E (1,2-b)**	20.9 (21.9)	22.0 (22.5)	24.0 (23.8)
**TS-F (1,4)**	47.4 (47.9)	47.9 (46.3)	48.5 (45.1)

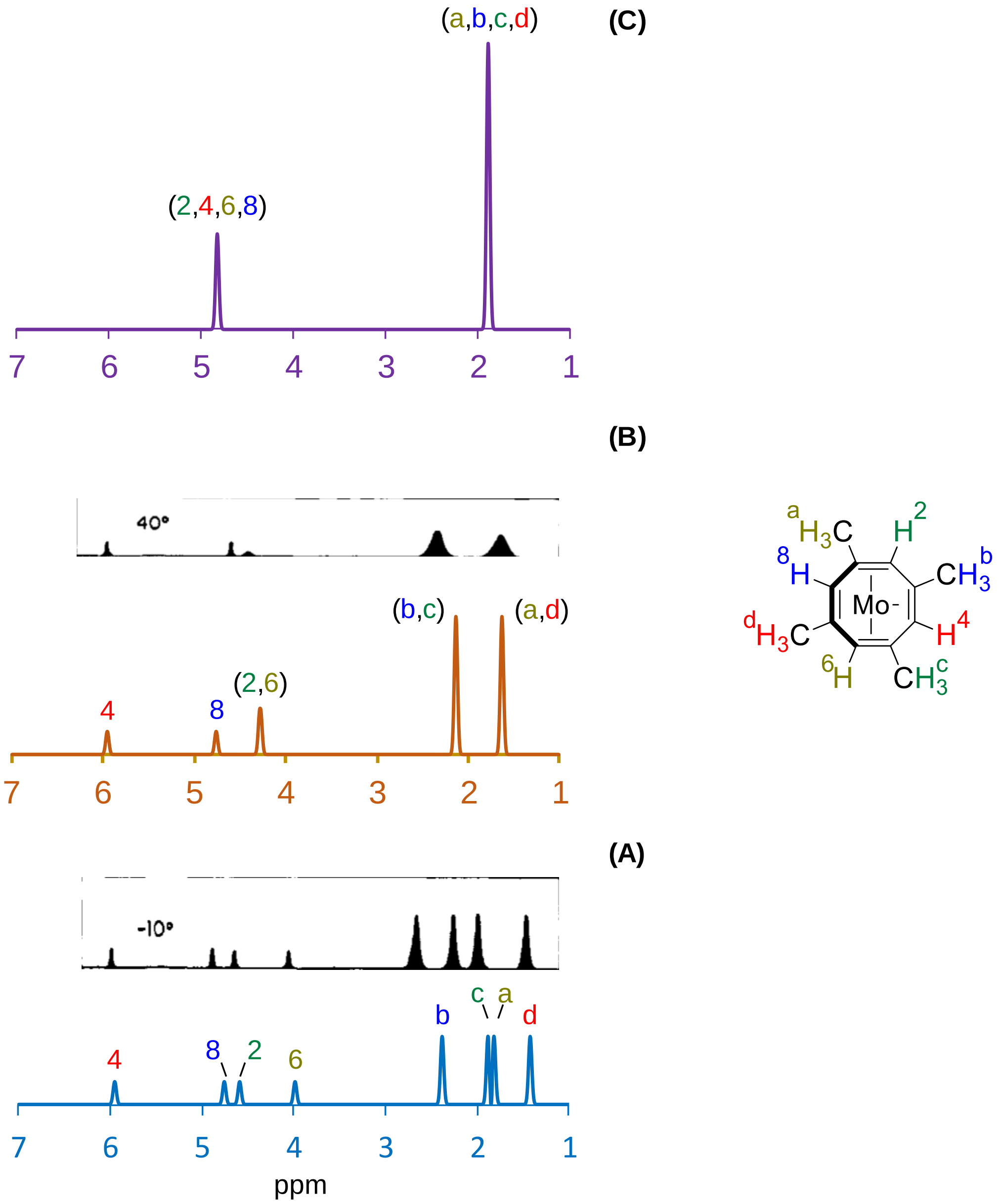

Figure S5. Simulated (colored) gas-phase and experimental (black) ^{1}H-NMR for the lowest energy structure of (TMCOT)Mo(CO)$_3$ at the GIAO-PBEPBE/BS6//PBEPBE/BS1 level of theory. Results given for low-temperature limit (**A**, –10° C), low temperature (**B**, 40° C), and high-temperature limit (**C**, >80° C). Experimental ^{1}H-NMR for high temperature limit not reported due to thermal decomposition of (TMCOT)Mo(CO)$_3$ above 80° C. Experimental spectra adapted in part with permission from Cotton, F. A.; Faller, J. W.; Musco, A. Stereochemically Nonrigid Organometallic Compounds. II. 1,3,5,7-Tetramethylcyclo-Octatetraenemolybdenum Tricarbonyl. *J. Am. Chem. Soc.* **1966**, *88* (19), 4506–4507. Copyright (1966) American Chemical Society.

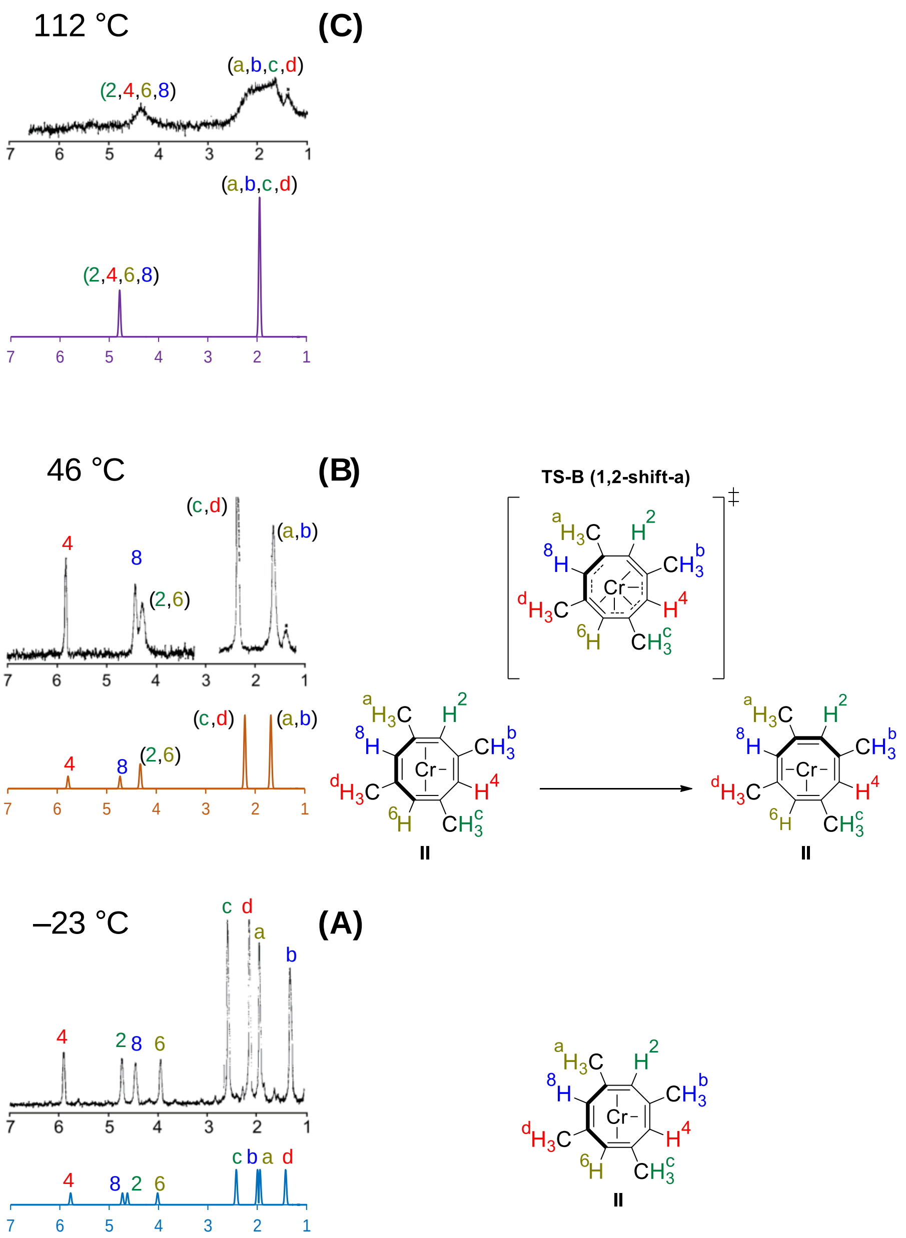

Figure S6. Simulated gas-phase (colored, GIAO-PBEPBE/BS6//PBEPBE/BS1) and experimental ^{1}H-NMR for (TMCOT)Cr(CO)$_3$ in three temperature regimes (A, B, C) with corresponding fluxional processes. (CO)$_3$ omitted for clarity. All chemical shifts are relative to tetramethylsilane (TMS). Experimental spectra adapted in part with permission from Cotton, F. A.; Faller, J. W.; Musco, A., Stereochemically nonrigid organometallic molecules. XII. Temperature dependence of the proton nuclear magnetic resonance spectra of the 1,3,5,7-tetramethylcyclooctatetraene tricarbonyl compounds of chromium, molybdenum, and tungsten *J. Am. Chem. Soc.*, **1968**, *90* (6), 1438-1444. Copyright (1968) American Chemical Society.

Table S8. Experimental and computed ^{1}H-NMR chemical shifts in (TMCOT)Cr$(CO)_3$ and (TMCOT)Mo$(CO)_3$ at the GIAO-PBEPBE/BS6//PBEPBE/auto/BS1 level of theory (measured in ppm; relative to TMS).

	**(TMCOT)Cr(CO)₃**		**(TMCOT)Mo(CO)₃**	
**Low-temperature limit**	**Expt (–23 °C)**	**Comp**	**Expt (–10 °C)**	**Comp**
H(2)	4.7	4.6	4.9	4.8
H(8)	4.3	4.7	4.6	4.6
H(4)	5.9	5.8	6.0	5.9
H(6)	3.9	4.0	4.0	4.0
CH_3(a)	2.6	2.4	1.4	1.4
CH_3(b)	1.9	1.9	1.9	1.8
CH_3(c)	1.3	1.4	2.6	2.3
CH_3(d)	2.1	2.0	2.2	1.9
**Low temperature**	**Exp (46 °C)**	**Comp**	**Exp (40 °C)**	**Comp**
Average of H(2, 6)	4.2	4.3	4.4	4.3
H(8)	4.4	4.7	4.6	4.8
H(4)	5.6	5.8	6.0	5.9
Average of CH_3(a, b)	2.3	2.2	1.7	1.6
Average of CH_3(c, d)	1.6	1.7	2.4	2.1
**High-temperature limit**	**Exp (112 °C)**	**Comp**	**Exp (> 80 °C)**	**Comp**
Average of H(2, 4, 6, 8)	4.3	4.8	–	4.8
Average of CH_3(a, b, c, d)	1.9	2.0	–	1.9

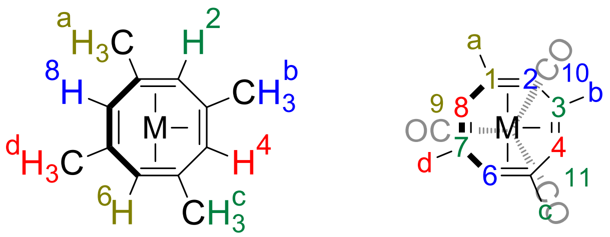

Figure S7. 2D-representation of (TMCOT)M$(CO)_3$ (M = Cr, Mo) with hydrogens labelled. $(CO)_3$ omitted for clarity.

Table S9. Computed chemical shifts for ^{13}C-NMR in (TMCOT)Cr(CO)$_3$ at the GIAO-PBEPBE/BS6//PBEPBE/auto/BS1 (relative to TMS). See Figure S for appropriate atom labelling.

Chemical Shift (ppm)	C(1)	C(2)	C(3)	C(4)	C(5)	C(6)	C(7)	C(8)	C(9)	C(10)	C(11)	C(a)	C(b)	C(c)	C(d)
**Expt.**	114.2	104.7	112.0	106.7	112.9	89.5	139.8	126.3	–	–	–	26.7	29.5	31.6	23.2
**Comp.**	123.7	111.6	120.3	113.4	121.8	98.4	150.6	134.7	245.3	226.6	245.3	29.5	33.9	35.0	25.8

*Experimental ^{13}C chemical shifts reported at –24 °C. No experimental ^{13}C chemical shift for CO groups were reported.

Table S10. Basis Sets Used in Study

Basis Set	
BS1	6-31G(d') for H, C, O<br>mod-LANL2DZ(f) with ECP for transition metals
BS2	6-311++G(2df,2p) for H, C, O<br>mod-LANL2TZ uncontracted to [4s4p3d] for Cr
BS3	cc-pVTZ for all atoms
BS4	aug-cc-pVDZ for H, C, O<br>LANL2DZ with LANL2DZ ECP for Cr
BS5	aug-cc-pVDZ for H, C, O<br>SDD with ECP for Cr
BS6	IGLO-II for H, C, O<br>LANL08 with ECP for Cr, Mo<br>LANl2DZ with ECP for Si

Molecular Coordinates for (COT)Cr$(CO)_3$ (Å)

PBE0/BS1

```
I
Cr   -0.047386 -0.350179  0.000000
C     1.792849 -0.230768  0.000000
O     2.936391 -0.104733  0.000000
C    -0.033948 -1.738162  1.212834
O    -0.011026 -2.634624  1.935405
C    -0.033948 -1.738162 -1.212834
O    -0.011026 -2.634624 -1.935405
C     0.388442  2.672437 -0.662865
C     0.251721  1.424132 -1.441166
C    -0.951791  0.746798 -1.646664
C    -1.985007  0.450346 -0.703748
C    -1.985007  0.450346  0.703748
C    -0.951791  0.746798  1.646664
C     0.251721  1.424132  1.441166
C     0.388442  2.672437  0.662865
H     0.465661  3.601360 -1.231928
H     0.961541  1.313251 -2.263119
H    -1.078535  0.227227 -2.597455
H    -2.832431 -0.084766 -1.131544
H    -2.832431 -0.084766  1.131544
H    -1.078535  0.227227  2.597455
H     0.961541  1.313251  2.263119
H     0.465661  3.601360  1.231928
    el energy= -735.149798374
          zpe= -734.986335
    th energy= -734.972586
  th enthalpy= -734.971641
  free energy= -735.026599

TS-1
Cr    0.310131  0.000000  0.018983
C     1.095356  1.232989 -1.097863
O     1.579159  2.013491 -1.793251
C     1.867616  0.000023  1.021824
O     2.830923  0.000037  1.649063
C     1.095361 -1.233030 -1.097816
O     1.579165 -2.013555 -1.793176
C    -2.706384 -0.661238 -0.645047
C    -1.420389 -1.375048 -0.475073
C    -0.885885 -1.651973  0.807857
C    -0.706449 -0.715281  1.849466
C    -0.706448  0.715333  1.849446
C    -0.885884  1.651996  0.807811
C    -1.420389  1.375036 -0.475111
C    -2.706384  0.661222 -0.645065
H    -3.618362 -1.257784 -0.723130
H    -1.251502 -2.176911 -1.197722
H    -0.417549 -2.619525  0.993061
H    -0.251507 -1.131364  2.748264
H    -0.251505  1.131441  2.748232
H    -0.417546  2.619553  0.992988
H    -1.251501  2.176878 -1.197783
H    -3.618362  1.257767 -0.723164
    el energy= -735.129678719
          zpe= -734.967003
    th energy= -734.954009
  th enthalpy= -734.953065
  free energy= -735.006011

TS-2
Cr    0.034057 -0.437936  0.000000
C    -1.170854 -0.998304  1.314095
O    -1.908003 -1.368251  2.111953
C    -1.170854 -0.998304 -1.314095
O    -1.908003 -1.368251 -2.111953
C     0.755537 -2.086267  0.000000
O     1.240084 -3.134776  0.000000
C    -0.040607  1.940984  1.663419
C     0.892715  0.810886  1.620733
C     1.877254  0.396858  0.713294
C     1.877254  0.396858 -0.713294
C     0.892715  0.810886 -1.620733
C    -0.040607  1.940984 -1.663419
C    -0.628680  2.720011 -0.729171
C    -0.628680  2.720011  0.729171
H    -0.353890  2.155900  2.685102
H     1.062675  0.403114  2.616229
H     2.633728 -0.261324  1.139553
H     2.633728 -0.261324 -1.139553
H     1.062675  0.403114 -2.616229
H    -0.353890  2.155900 -2.685102
H    -1.293090  3.478852 -1.141779
H    -1.293090  3.478852  1.141779
    el energy= -735.127176234
          zpe= -734.964222
    th energy= -734.950597
  th enthalpy= -734.949653
  free energy= -735.005093

TS-3
Cr   -0.010092 -0.469499  0.000000
C     1.869771 -0.409652  0.000000
O     3.013836 -0.349533  0.000000
C    -0.075570 -1.842036  1.217733
O    -0.114803 -2.726831  1.955049
C    -0.075570 -1.842036 -1.217733
O    -0.114803 -2.726831 -1.955049
C    -2.047149  0.285865  0.000000
C    -1.438474  0.489015  1.253561
C    -0.271033  1.222752  1.633620
C     0.246108  2.472932  1.228887
C     0.315174  3.120576  0.000000
C     0.246108  2.472932 -1.228887
C    -0.271033  1.222752 -1.633620
C    -1.438474  0.489015 -1.253561
H    -2.941106 -0.334294  0.000000
H    -1.926046 -0.033705  2.076872
H     0.050886  0.947388  2.637906
H     0.769443  2.998010  2.032970
H     0.761750  4.111780  0.000000
H     0.769443  2.998010 -2.032970
H     0.050886  0.947388 -2.637906
H    -1.926046 -0.033705 -2.076872
    el energy= -735.122989876
          zpe= -734.961852
    th energy= -734.947906
  th enthalpy= -734.946962
  free energy= -735.003058

TS-4
Cr   -0.059206  0.442055  0.000000
C    -1.592175  1.483257  0.000000
O    -2.513954  2.173374  0.000000
C     0.622660  1.559141  1.241339
O     1.065784  2.265894  2.043411
C     0.622660  1.559141 -1.241339
O     1.065784  2.265894 -2.043411
C    -1.494592 -1.642534 -0.677706
C    -0.462542 -2.152853 -1.587956
C     0.844500 -1.831234 -1.594021
C     1.561781 -0.919414 -0.694092
```

```
C     1.561781 -0.919414  0.694092
C     0.844500 -1.831234  1.594021
C    -0.462542 -2.152853  1.587956
C    -1.494592 -1.642534  0.677706
H    -2.403613 -1.285322 -1.165611
H    -0.836591 -2.771133 -2.405419
H     1.442166 -2.212310 -2.424329
H     2.383741 -0.384948 -1.169089
H     2.383741 -0.384948  1.169089
H     1.442166 -2.212310  2.424329
H    -0.836591 -2.771133  2.405419
H    -2.403613 -1.285322  1.165611
    el energy= -735.108933361
          zpe= -734.947626
    th energy= -734.933504
  th enthalpy= -734.932560
  free energy= -734.989541
```

```
TS-5
Cr    0.382049 -0.000318 -0.332554
C     1.366445 -1.528776 -0.546299
O     2.013263 -2.459863 -0.745464
C     1.423902 -0.000347  1.149780
O     2.111739 -0.000431  2.080484
C     1.368345  1.526906 -0.547007
O     2.016342  2.457104 -0.746435
C    -1.292121  1.290430 -1.128948
C    -1.295978  0.000872 -1.703259
C    -1.293409 -1.288798 -1.129173
C    -1.907995 -1.770460  0.121396
C    -1.636945 -1.237009  1.316672
C    -0.807391  0.000139  1.475153
C    -1.634994  1.238582  1.317114
C    -1.905475  1.772686  0.121957
H    -1.077127  2.078884 -1.851388
H    -0.890172  0.000822 -2.721611
H    -1.079138 -2.077354 -1.851718
H    -2.538245 -2.657651  0.046002
H    -2.060099 -1.680718  2.220206
H    -0.370478 -0.000409  2.479004
H    -2.056943  1.683104  2.220809
H    -2.534016  2.661121  0.046997
    el energy= -735.071838896
          zpe= -734.910833
    th energy= -734.897253
  th enthalpy= -734.896309
  free energy= -734.951098
```

```
TS-6
Cr   -0.766299  0.012947 -0.319934
C    -0.232826  0.011821  1.424946
O     0.080115  0.082902  2.530281
C    -1.605469  1.622479  0.122030
O    -2.079781  2.613259  0.456454
C    -2.192608 -1.054599  0.071969
O    -3.082539 -1.764261  0.259676
C     1.997524 -1.599727  0.477649
C     0.694821 -1.743794 -0.204947
C     0.337352 -1.313294 -1.493495
C     0.510007  0.005816 -2.023413
C     0.923385  1.202759 -1.430154
C     1.881882  1.564460 -0.391712
C     2.708394  0.854510  0.410969
C     2.841311 -0.567681  0.673283
H     2.328369 -2.549991  0.900303
H     0.200407 -2.673267  0.077919
H    -0.311768 -1.963662 -2.079582
H    -0.072897  0.166812 -2.935160
H     0.615176  2.070242 -2.012844
H     1.967870  2.646558 -0.289809
H     3.385575  1.463271  1.010365
H     3.753458 -0.822403  1.213194
    el energy= -735.109479356
          zpe= -734.947345
    th energy= -734.933640
  th enthalpy= -734.932696
  free energy= -734.988047
```

```
2
Cr   -0.875553  0.000033 -0.304650
C    -0.184098 -0.000025  1.409215
O     0.232186  0.000032  2.484271
C    -2.009346  1.425034  0.142342
O    -2.676839  2.326929  0.409551
C    -2.009994 -1.424474  0.142151
O    -2.677856 -2.326127  0.409267
C     2.034607 -1.663496  0.043727
C     0.883018 -1.630800 -0.858883
C     0.433302 -0.722114 -1.817720
C     0.433412  0.721329 -1.817999
C     0.883440  1.630420 -0.859707
C     2.034831  1.663298  0.043087
C     2.877753  0.726626  0.547517
C     2.877693 -0.726773  0.547749
H     2.266229 -2.684222  0.349721
H     0.476162 -2.628785 -1.018201
H    -0.200522 -1.155691 -2.595440
H    -0.200430  1.154615 -2.595877
H     0.476726  2.628378 -1.019513
H     2.266478  2.684105  0.348803
H     3.690494  1.140884  1.144132
H     3.690502 -1.140900  1.144364
    el energy= -735.896316253
          zpe= -735.735743
    th energy= -735.720950
  th enthalpy= -735.720006
  free energy= -735.778222
```

PBE0/BS3

```
I
Cr   -0.045253 -0.347029  0.000000
C     1.809447 -0.235706  0.000000
O     2.948540 -0.120341  0.000000
C    -0.040733 -1.746705  1.213008
O    -0.023974 -2.647616  1.922416
C    -0.040733 -1.746705 -1.213008
O    -0.023974 -2.647616 -1.922416
C     0.394486  2.669977 -0.658862
C     0.244461  1.432614 -1.441695
C    -0.953293  0.756568 -1.641569
C    -1.978682  0.457504 -0.701031
C    -1.978682  0.457504  0.701031
C    -0.953293  0.756568  1.641569
C     0.244461  1.432614  1.441695
C     0.394486  2.669977  0.658862
H     0.485774  3.596241 -1.221298
H     0.944533  1.330614 -2.266691
H    -1.087278  0.252302 -2.593810
H    -2.828136 -0.065143 -1.127794
H    -2.828136 -0.065143  1.127794
H    -1.087278  0.252302  2.593810
H     0.944533  1.330614  2.266691
H     0.485774  3.596241  1.221298
    el energy= -1693.41122721
          zpe= -1693.248461
```

```
    th energy= -1693.234711
  th enthalpy= -1693.233767
  free energy= -1693.288845

TS-1
Cr   -0.022176 -0.307633  0.000000
C     1.098150 -1.101654  1.240198
O     1.789672 -1.589054  2.014344
C    -1.032211 -1.874149  0.000000
O    -1.648874 -2.837940  0.000000
C     1.098150 -1.101654 -1.240198
O     1.789672 -1.589054 -2.014344
C     0.659856  2.697981 -0.657193
C     0.471657  1.421958 -1.374191
C    -0.809403  0.896086 -1.645781
C    -1.845105  0.716888 -0.712255
C    -1.845105  0.716888  0.712255
C    -0.809403  0.896086  1.645781
C     0.471657  1.421958  1.374191
C     0.659856  2.697981  0.657193
H     0.754212  3.606867 -1.247713
H     1.185965  1.254784 -2.177149
H    -1.003388  0.449226 -2.616188
H    -2.746845  0.279796 -1.128177
H    -2.746845  0.279796  1.128177
H    -1.003388  0.449226  2.616188
H     1.185965  1.254784  2.177149
H     0.754212  3.606867  1.247713
    el energy= -1693.39215710
          zpe= -1693.230127
    th energy= -1693.217163
  th enthalpy= -1693.216219
  free energy= -1693.269197

TS-2
Cr    0.040766 -0.434714  0.000000
C    -1.163135 -1.022661  1.319652
O    -1.886009 -1.416544  2.111424
C    -1.163135 -1.022661 -1.319652
O    -1.886009 -1.416544 -2.111424
C     0.778068 -2.083933  0.000000
O     1.261671 -3.127869  0.000000
C    -0.064241  1.945093  1.654793
C     0.890589  0.842028  1.615448
C     1.871545  0.434609  0.709825
C     1.871545  0.434609 -0.709825
C     0.890589  0.842028 -1.615448
C    -0.064241  1.945093 -1.654793
C    -0.664046  2.709675 -0.725799
C    -0.664046  2.709675  0.725799
H    -0.383148  2.152262  2.671597
H     1.072535  0.449911  2.609737
H     2.645860 -0.192914  1.137438
H     2.645860 -0.192914 -1.137438
H     1.072535  0.449911 -2.609737
H    -0.383148  2.152262 -2.671597
H    -1.341534  3.450472 -1.138037
H    -1.341534  3.450472  1.138037
    el energy= -1693.38908154
          zpe= -1693.226883
    th energy= -1693.213236
  th enthalpy= -1693.212292
  free energy= -1693.267947

TS-3
Cr   -0.007357 -0.457183  0.000000
C     1.891484 -0.424342  0.000000
O     3.031051 -0.400048  0.000000
C    -0.083945 -1.841810  1.215821
O    -0.133269 -2.734442  1.935118
C    -0.083945 -1.841810 -1.215821
O    -0.133269 -2.734442 -1.935118
C    -2.046099  0.294569  0.000000
C    -1.450199  0.507815  1.250921
C    -0.295146  1.242843  1.640601
C     0.261938  2.463792  1.226644
C     0.362381  3.097465  0.000000
C     0.261938  2.463792 -1.226644
C    -0.295146  1.242843 -1.640601
C    -1.450199  0.507815 -1.250921
H    -2.941166 -0.315361  0.000000
H    -1.943490 -0.005085  2.069920
H     0.008071  0.977109  2.648366
H     0.795292  2.974992  2.026653
H     0.843517  4.067334  0.000000
H     0.795292  2.974992 -2.026653
H     0.008071  0.977109 -2.648366
H    -1.943490 -0.005085 -2.069920
    el energy= -1693.38518754
          zpe= -1693.224728
    th energy= -1693.210764
  th enthalpy= -1693.209820
  free energy= -1693.266117

TS-4
Cr   -0.059766  0.432854  0.000000
C    -1.590165  1.490371  0.000000
O    -2.493972  2.196050  0.000000
C     0.625629  1.561029  1.237165
O     1.068321  2.278677  2.023189
C     0.625629  1.561029 -1.237165
O     1.068321  2.278677 -2.023189
C    -1.492124 -1.641567 -0.674166
C    -0.468583 -2.142594 -1.588334
C     0.834182 -1.834722 -1.593424
C     1.561109 -0.944293 -0.690853
C     1.561109 -0.944293  0.690853
C     0.834182 -1.834722  1.593424
C    -0.468583 -2.142594  1.588334
C    -1.492124 -1.641567  0.674166
H    -2.403672 -1.297706 -1.156197
H    -0.847202 -2.740625 -2.412519
H     1.421085 -2.206499 -2.429524
H     2.385519 -0.421255 -1.162725
H     2.385519 -0.421255  1.162725
H     1.421085 -2.206499  2.429524
H    -0.847202 -2.740625  2.412519
H    -2.403672 -1.297706  1.156197
    el energy= -1693.36874019
          zpe= -1693.208124
    th energy= -1693.193999
  th enthalpy= -1693.193055
  free energy= -1693.250206

TS-5
Cr    0.339404  0.383199  0.000000
C     0.554602  1.394439  1.522448
O     0.735601  2.045680  2.447702
C    -1.154829  1.420179  0.000000
O    -2.086286  2.098804  0.000000
C     0.554602  1.394439 -1.522448
O     0.735601  2.045680 -2.447702
C     1.128399 -1.316018 -1.286005
C     1.693993 -1.317967  0.000000
C     1.128399 -1.316018  1.286005
C    -0.125317 -1.908234  1.771444
C    -1.312381 -1.642561  1.237100
C    -1.470701 -0.822788  0.000000
C    -1.312381 -1.642561 -1.237100
C    -0.125317 -1.908234 -1.771444
```

```
H     1.853203 -1.116452 -2.069044
H     2.720663 -0.947563  0.000000
H     1.853203 -1.116452  2.069044
H    -0.056678 -2.526552  2.661990
H    -2.212670 -2.053992  1.686678
H    -2.467830 -0.384583  0.000000
H    -2.212670 -2.053992 -1.686678
```

PBEPBE/auto/BS1

```
I
Cr   -0.053397 -0.352388  0.000000
C     1.791775 -0.274455  0.000000
O     2.956245 -0.192955  0.000000
C    -0.060357 -1.753806  1.195051
O    -0.044046 -2.681154  1.908587
C    -0.060357 -1.753806 -1.195051
O    -0.044046 -2.681154 -1.908587
C     0.429587  2.700629 -0.668771
C     0.272816  1.453148 -1.455678
C    -0.946660  0.777454 -1.659266
C    -1.992670  0.501223 -0.709596
C    -1.992670  0.501223  0.709596
C    -0.946660  0.777454  1.659266
C     0.272816  1.453148  1.455678
C     0.429587  2.700629  0.668771
H     0.528432  3.636203 -1.240363
H     0.987986  1.329244 -2.282275
H    -1.083831  0.258859 -2.618499
H    -2.856074 -0.023130 -1.140303
H    -2.856074 -0.023130  1.140303
H    -1.083831  0.258859  2.618499
H     0.987986  1.329244  2.282275
H     0.528432  3.636203  1.240363
    el energy= -735.218624797
          zpe= -735.061293
    th energy= -735.047063
  th enthalpy= -735.046119
  free energy= -735.102078

TS-1
Cr   -0.311404  0.000036 -0.028314
C    -1.133581  1.222759  1.072975
O    -1.666240  1.997377  1.769280
C    -1.861648 -0.001347 -1.039936
O    -2.847917 -0.002109 -1.665224
C    -1.132487 -1.221230  1.075343
O    -1.664406 -1.995115  1.773037
C     2.730094 -0.666608  0.699252
C     1.447012 -1.390853  0.498937
C     0.922657 -1.663826 -0.801345
C     0.759318 -0.721048 -1.854705
C     0.759421  0.719775 -1.855200
C     0.922776  1.663248 -0.802473
C     1.447045  1.391505  0.498090
C     2.730071  0.667365  0.699049
H     3.648452 -1.265788  0.802213
H     1.255953 -2.195547  1.225164
H     0.455038 -2.639620 -0.994742
H     0.319494 -1.141625 -2.768830
H     0.319664  1.139691 -2.769656
H     0.455182  2.638885 -0.996738
H     1.255928  2.196895  1.223521
H    -0.056678 -2.526552 -2.661990
    el energy= -1693.33486428
          zpe= -1693.174562
    th energy= -1693.160999
  th enthalpy= -1693.160055
  free energy= -1693.214782

H     3.648411  1.266589  0.801927
    el energy= -735.200181685
          zpe= -735.043660
    th energy= -735.030153
  th enthalpy= -735.029208
  free energy= -735.083254

TS-2
Cr    0.027808 -0.444925  0.000000
C    -1.129959 -1.074035  1.320346
O    -1.849495 -1.509754  2.127971
C    -1.129959 -1.074035 -1.320346
O    -1.849495 -1.509754 -2.127971
C     0.786000 -2.068672  0.000000
O     1.293757 -3.127992  0.000000
C    -0.084169  1.990242  1.675048
C     0.870729  0.874502  1.634465
C     1.862184  0.457328  0.718644
C     1.862184  0.457328 -0.718644
C     0.870729  0.874502 -1.634465
C    -0.084169  1.990242 -1.675048
C    -0.682087  2.774580 -0.730871
C    -0.682087  2.774580  0.730871
H    -0.411247  2.196544  2.703100
H     1.043012  0.461023  2.636244
H     2.635654 -0.190912  1.150443
H     2.635654 -0.190912 -1.150443
H     1.043012  0.461023 -2.636244
H    -0.411247  2.196544 -2.703100
H    -1.358380  3.532760 -1.147741
H    -1.358380  3.532760  1.147741
    el energy= -735.198189551
          zpe= -735.041114
    th energy= -735.027043
  th enthalpy= -735.026098
  free energy= -735.082544

TS-3
Cr   -0.006078 -0.478057  0.000000
C     1.872407 -0.466279  0.000000
O     3.036203 -0.455748  0.000000
C    -0.104528 -1.864087  1.192002
O    -0.163964 -2.783061  1.916844
C    -0.104528 -1.864087 -1.192002
O    -0.163964 -2.783061 -1.916844
C    -2.043439  0.332011  0.000000
C    -1.431605  0.533175  1.265595
C    -0.253195  1.262637  1.646892
C     0.268075  2.523650  1.240292
C     0.342025  3.169324  0.000000
C     0.268075  2.523650 -1.240292
C    -0.253195  1.262637 -1.646892
C    -1.431605  0.533175 -1.265595
```

```
H    -2.951089 -0.282873  0.000000
H    -1.925359  0.005676  2.092893
H     0.076606  0.979144  2.655379
H     0.786911  3.057250  2.053103
H     0.783523  4.172218  0.000000
H     0.786911  3.057250 -2.053103
H     0.076606  0.979144 -2.655379
H    -1.925359  0.005676 -2.092893
    el energy= -735.198327495
          zpe= -735.042699
    th energy= -735.028343
  th enthalpy= -735.027399
  free energy= -735.084461

TS-4
Cr   -0.053243  0.421556  0.000000
C    -1.608039  1.426423  0.000000
O    -2.557207  2.109518  0.000000
C     0.600236  1.574062  1.216495
O     1.034495  2.324644  2.010459
C     0.600236  1.574062 -1.216495
O     1.034495  2.324644 -2.010459
C    -1.490423 -1.672075 -0.685667
C    -0.453363 -2.130123 -1.622680
C     0.868401 -1.805700 -1.627242
C     1.606311 -0.933036 -0.700348
C     1.606311 -0.933036  0.700348
C     0.868401 -1.805700  1.627242
C    -0.453363 -2.130123  1.622680
C    -1.490423 -1.672075  0.685667
H    -2.426209 -1.350456 -1.165759
H    -0.834018 -2.696614 -2.484698
H     1.451429 -2.133570 -2.500691
H     2.437717 -0.391287 -1.169815
H     2.437717 -0.391287  1.169815
H     1.451429 -2.133570  2.500691
H    -0.834018 -2.696614  2.484698
```

PBEPBE/BS2

```
I
Cr   -0.055821 -0.347342  0.000000
C     1.801726 -0.304964  0.000000
O     2.961053 -0.253093  0.000000
C    -0.079778 -1.757488  1.197275
O    -0.071014 -2.685655  1.899302
C    -0.079778 -1.757488 -1.197275
O    -0.071014 -2.685655 -1.899302
C     0.456955  2.693087 -0.664576
C     0.276968  1.458572 -1.455814
C    -0.942132  0.796059 -1.654122
C    -1.983271  0.526768 -0.706489
C    -1.983271  0.526768  0.706489
C    -0.942132  0.796059  1.654122
C     0.276968  1.458572  1.455814
C     0.456955  2.693087  0.664576
H     0.576829  3.623773 -1.229246
H     0.983830  1.334307 -2.281639
H    -1.090864  0.294313 -2.613765
H    -2.853675  0.026229 -1.136237
H    -2.853675  0.026229  1.136237
H    -1.090864  0.294313  2.613765
H     0.983830  1.334307  2.281639
```

```
H    -2.426209 -1.350456  1.165759
    el energy= -735.179977018
          zpe= -735.024338
    th energy= -735.009827
  th enthalpy= -735.008883
  free energy= -735.066692

TS-5
Cr    0.351482 -0.000448 -0.288416
C     1.369976 -1.496266 -0.552929
O     2.062771 -2.407907 -0.799886
C     1.461479  0.000604  1.157993
O     2.181570  0.001065  2.086392
C     1.370596  1.494632 -0.554604
O     2.063652  2.405842 -0.802411
C    -1.324353  1.302057 -1.153218
C    -1.302506  0.001096 -1.727667
C    -1.324905 -1.300447 -1.154556
C    -1.901952 -1.802336  0.105374
C    -1.650274 -1.269159  1.323804
C    -0.895562 -0.000542  1.538402
C    -1.649155  1.269069  1.325284
C    -1.900326  1.803559  0.107313
H    -1.103374  2.094123 -1.881338
H    -0.906809  0.001621 -2.756107
H    -1.104032 -2.091842 -1.883487
H    -2.447218 -2.754664  0.040286
H    -1.984617 -1.805042  2.224503
H    -0.445676 -0.001100  2.544670
H    -1.981899  1.805188  2.226359
H    -2.444003  2.756862  0.043184
    el energy= -735.150526632
          zpe= -734.995828
    th energy= -734.981618
  th enthalpy= -734.980673
  free energy= -735.036989


H     0.576829  3.623773  1.229246
    el energy= -735.459353623
          zpe= -735.302462
    th energy= -735.288282
  th enthalpy= -735.287337
  free energy= -735.343273

TS-1
Cr   -0.032115 -0.306246  0.000000
C     1.069437 -1.143235  1.225867
O     1.757254 -1.686785  1.990529
C    -1.049983 -1.863741  0.000000
O    -1.664310 -2.848837  0.000000
C     1.069437 -1.143235 -1.225867
O     1.757254 -1.686785 -1.990529
C     0.719191  2.720285 -0.662745
C     0.498731  1.448736 -1.390566
C    -0.799648  0.934608 -1.657513
C    -1.847251  0.776276 -0.717368
C    -1.847251  0.776276  0.717368
C    -0.799648  0.934608  1.657513
C     0.498731  1.448736  1.390566
C     0.719191  2.720285  0.662745
```

```
H      0.840744  3.633628 -1.255761
H      1.217730  1.257307 -2.193551
H     -1.001940  0.487115 -2.634270
H     -2.764766  0.357737 -1.136781
H     -2.764766  0.357737  1.136781
H     -1.001940  0.487115  2.634270
H      1.217730  1.257307  2.193551
H      0.840744  3.633628  1.255761
    el energy= -735.441512750
          zpe= -735.285408
    th energy= -735.271974
  th enthalpy= -735.271030
  free energy= -735.324976

TS-2
Cr     0.031650 -0.435678  0.000000
C     -1.118756 -1.095334  1.325567
O     -1.815586 -1.559961  2.126999
C     -1.118756 -1.095334 -1.325567
O     -1.815586 -1.559961 -2.126999
C      0.802321 -2.062637  0.000000
O      1.300996 -3.119155  0.000000
C     -0.107239  1.991301  1.666084
C      0.866182  0.900231  1.628280
C      1.855015  0.492562  0.715368
C      1.855015  0.492562 -0.715368
C      0.866182  0.900231 -1.628280
C     -0.107239  1.991301 -1.666084
C     -0.715823  2.761250 -0.727734
C     -0.715823  2.761250  0.727734
H     -0.434934  2.193238  2.688852
H      1.048785  0.500463  2.627305
H      2.644903 -0.124980  1.147296
H      2.644903 -0.124980 -1.147296
H      1.048785  0.500463 -2.627305
H     -0.434934  2.193238 -2.688852
H     -1.401083  3.503578 -1.143780
H     -1.401083  3.503578  1.143780
    el energy= -735.438707789
          zpe= -735.282152
    th energy= -735.268112
  th enthalpy= -735.267168
  free energy= -735.323658

TS-3
Cr    -0.006947 -0.465411  0.000000
C      1.885096 -0.495541  0.000000
O      3.041372 -0.529742  0.000000
C     -0.120627 -1.861292  1.192153
O     -0.188594 -2.784312  1.900392
C     -0.120627 -1.861292 -1.192153
O     -0.188594 -2.784312 -1.900392
C     -2.040747  0.355514  0.000000
C     -1.437737  0.559712  1.261923
C     -0.260880  1.276190  1.647386
C      0.292575  2.513038  1.236147
C      0.389624  3.148765  0.000000
C      0.292575  2.513038 -1.236147
C     -0.260880  1.276190 -1.647386
C     -1.437737  0.559712 -1.261923
H     -2.956120 -0.236902  0.000000
H     -1.941619  0.048994  2.085397
H      0.055037  0.996192  2.655231
H      0.820775  3.032785  2.043943
H      0.857169  4.133536  0.000000
H      0.820775  3.032785 -2.043943
H      0.055037  0.996192 -2.655231
H     -1.941619  0.048994 -2.085397
    el energy= -735.438733464
          zpe= -735.283578
    th energy= -735.269258
  th enthalpy= -735.268314
  free energy= -735.325448

TS-4
Cr    -0.049483  0.407359  0.000000
C     -1.604876  1.423283  0.000000
O     -2.540292  2.113917  0.000000
C      0.604067  1.576370  1.211574
O      1.031591  2.343685  1.983437
C      0.604067  1.576370 -1.211574
O      1.031591  2.343685 -1.983437
C     -1.489780 -1.660406 -0.682170
C     -0.462133 -2.110619 -1.622537
C      0.857007 -1.807290 -1.626242
C      1.610869 -0.963779 -0.696150
C      1.610869 -0.963779  0.696150
C      0.857007 -1.807290  1.626242
C     -0.462133 -2.110619  1.622537
C     -1.489780 -1.660406  0.682170
H     -2.426258 -1.352054 -1.156421
H     -0.848708 -2.654074 -2.489474
H      1.427266 -2.128402 -2.503310
H      2.444397 -0.434428 -1.162110
H      2.444397 -0.434428  1.162110
H      1.427266 -2.128402  2.503310
H     -0.848708 -2.654074  2.489474
H     -2.426258 -1.352054  1.156421
    el energy= -735.419810391
          zpe= -735.264726
    th energy= -735.250231
  th enthalpy= -735.249287
  free energy= -735.307221

TS-5
Cr     0.286614  0.340723  0.000000
C      0.567717  1.385630  1.484991
O      0.806441  2.083453  2.386040
C     -1.164475  1.455441  0.000000
O     -2.084257  2.176053  0.000000
C      0.567717  1.385630 -1.484991
O      0.806441  2.083453 -2.386040
C      1.155307 -1.348053 -1.297366
C      1.720624 -1.325167  0.000000
C      1.155307 -1.348053  1.297366
C     -0.111426 -1.881253  1.807480
C     -1.324712 -1.639200  1.274614
C     -1.553739 -0.919864  0.000000
C     -1.324712 -1.639200 -1.274614
C     -0.111426 -1.881253 -1.807480
H      1.887712 -1.147621 -2.082262
H      2.760364 -0.980051  0.000000
```

```
H     1.887712 -1.147621  2.082262
H    -0.056825 -2.388187  2.775053
H    -2.216772 -1.932104  1.836354
H    -2.553408 -0.473084  0.000000
H    -2.216772 -1.932104 -1.836354
H    -0.056825 -2.388187 -2.775053
    el energy= -735.391210914
```

PBEPBE/BS3

```
I
Cr   -0.053200 -0.346894  0.000000
C     1.805829 -0.285340  0.000000
O     2.965223 -0.220184  0.000000
C    -0.071181 -1.760130  1.195545
O    -0.060724 -2.691312  1.895220
C    -0.071181 -1.760130 -1.195545
O    -0.060724 -2.691312 -1.895220
C     0.441188  2.694941 -0.664416
C     0.270660  1.457270 -1.452985
C    -0.945732  0.788674 -1.653741
C    -1.984904  0.514299 -0.706563
C    -1.984904  0.514299  0.706563
C    -0.945732  0.788674  1.653741
C     0.270660  1.457270  1.452985
C     0.441188  2.694941  0.664416
H     0.551915  3.626210 -1.230226
H     0.977625  1.338947 -2.279819
H    -1.092194  0.287650 -2.614189
H    -2.851721  0.006855 -1.136080
H    -2.851721  0.006855  1.136080
H    -1.092194  0.287650  2.614189
H     0.977625  1.338947  2.279819
H     0.551915  3.626210  1.230226
    el energy= -1693.44464560
          zpe= -1693.287782
    th energy= -1693.273601
  th enthalpy= -1693.272656
  free energy= -1693.328616

TS-1
Cr   -0.032046 -0.307188  0.000000
C     1.073459 -1.137281  1.229431
O     1.765461 -1.671917  1.997760
C    -1.047473 -1.869240  0.000000
O    -1.658179 -2.857554  0.000000
C     1.073459 -1.137281 -1.229431
O     1.765461 -1.671917 -1.997760
C     0.711407  2.720574 -0.662632
C     0.495145  1.446759 -1.387631
C    -0.802843  0.930888 -1.656798
C    -1.849844  0.768305 -0.717276
C    -1.849844  0.768305  0.717276
C    -0.802843  0.930888  1.656798
C     0.495145  1.446759  1.387631
C     0.711407  2.720574  0.662632
H     0.828572  3.634289 -1.256215
H     1.213651  1.258100 -2.192015
H    -1.004444  0.486021 -2.635028
H    -2.765728  0.345646 -1.136704
H    -2.765728  0.345646  1.136704
H    -1.004444  0.486021  2.635028
H     1.213651  1.258100  2.192015
```

```
          zpe= -735.237078
    th energy= -735.222832
  th enthalpy= -735.221888
  free energy= -735.278438
```

```
H     0.828572  3.634289  1.256215
    el energy= -1693.42716645
          zpe= -1693.271070
    th energy= -1693.257640
  th enthalpy= -1693.256695
  free energy= -1693.310651

TS-2
Cr    0.030806 -0.440811  0.000000
C    -1.120408 -1.097175  1.328552
O    -1.820041 -1.555456  2.132630
C    -1.120408 -1.097175 -1.328552
O    -1.820041 -1.555456 -2.132630
C     0.801887 -2.066924  0.000000
O     1.305411 -3.122468  0.000000
C    -0.104260  1.995557  1.665297
C     0.865018  0.901211  1.627115
C     1.853518  0.489628  0.715032
C     1.853518  0.489628 -0.715032
C     0.865018  0.901211 -1.627115
C    -0.104260  1.995557 -1.665297
C    -0.710238  2.767989 -0.727430
C    -0.710238  2.767989  0.727430
H    -0.433153  2.195923  2.688135
H     1.046404  0.502162  2.626914
H     2.641887 -0.129581  1.147794
H     2.641887 -0.129581 -1.147794
H     1.046404  0.502162 -2.626914
H    -0.433153  2.195923 -2.688135
H    -1.393571  3.512261 -1.143662
H    -1.393571  3.512261  1.143662
    el energy= -1693.42374242
          zpe= -1693.267227
    th energy= -1693.253172
  th enthalpy= -1693.252227
  free energy= -1693.308778

TS-3
Cr   -0.005235 -0.465827  0.000000
C     1.890158 -0.486714  0.000000
O     3.047395 -0.513176  0.000000
C    -0.116850 -1.864179  1.189832
O    -0.185139 -2.789756  1.896462
C    -0.116850 -1.864179 -1.189832
O    -0.185139 -2.789756 -1.896462
C    -2.041040  0.348462  0.000000
C    -1.439756  0.556011  1.262016
C    -0.266334  1.277741  1.647330
C     0.286538  2.514796  1.235937
C     0.384290  3.149905  0.000000
C     0.286538  2.514796 -1.235937
C    -0.266334  1.277741 -1.647330
C    -1.439756  0.556011 -1.262016
```

```
H    -2.952303 -0.250744  0.000000
H    -1.940752  0.042452  2.085633
H     0.048759  1.000509  2.656407
H     0.814049  3.034806  2.044158
H     0.853280  4.134219  0.000000
H     0.814049  3.034806 -2.044158
H     0.048759  1.000509 -2.656407
H    -1.940752  0.042452 -2.085633
    el energy= -1693.42403373
          zpe= -1693.268917
    th energy= -1693.254572
  th enthalpy= -1693.253628
  free energy= -1693.310894

TS-4
Cr   -0.047008  0.402558  0.000000
C    -1.612808  1.405349  0.000000
O    -2.557935  2.083911  0.000000
C     0.600526  1.575298  1.210908
O     1.026621  2.342092  1.985652
C     0.600526  1.575298 -1.210908
O     1.026621  2.342092 -1.985652
C    -1.485582 -1.660661 -0.682533
C    -0.459068 -2.094038 -1.630019
C     0.860803 -1.792443 -1.632876
C     1.619248 -0.962174 -0.696216
C     1.619248 -0.962174  0.696216
C     0.860803 -1.792443  1.632876
C    -0.459068 -2.094038  1.630019
C    -1.485582 -1.660661  0.682533
H    -2.427169 -1.362404 -1.153098
H    -0.847774 -2.621078 -2.506194
H     1.426316 -2.098301 -2.518527
H     2.454366 -0.433228 -1.160250
H     2.454366 -0.433228  1.160250
H     1.426316 -2.098301  2.518527
H    -0.847774 -2.621078  2.506194
H    -2.427169 -1.362404  1.153098
    el energy= -1693.40372685
          zpe= -1693.248588
    th energy= -1693.234081
  th enthalpy= -1693.233136
  free energy= -1693.291160

TS-5
Cr    0.287456  0.344507  0.000000
C     0.567250  1.388676  1.487105
O     0.805147  2.084136  2.391674
C    -1.167191  1.456020  0.000000
O    -2.092688  2.170995  0.000000
C     0.567250  1.388676 -1.487105
O     0.805147  2.084136 -2.391674
C     1.155661 -1.347216 -1.297602
C     1.720189 -1.323798  0.000000
C     1.155661 -1.347216  1.297602
C    -0.109395 -1.882700  1.808768
C    -1.322368 -1.644393  1.274489
C    -1.550382 -0.924564  0.000000
C    -1.322368 -1.644393 -1.274489
C    -0.109395 -1.882700 -1.808768
H     1.888581 -1.145401 -2.081880
H     2.759261 -0.975820  0.000000
H     1.888581 -1.145401  2.081880
H    -0.053330 -2.388493  2.776957
H    -2.214510 -1.940115  1.834803
H    -2.550013 -0.476839  0.000000
H    -2.214510 -1.940115 -1.834803
H    -0.053330 -2.388493 -2.776957
    el energy= -1693.37670695
          zpe= -1693.222555
    th energy= -1693.208321
  th enthalpy= -1693.207376
  free energy= -1693.263862
```

B3LYP/BS1

```
I
Cr   -0.035449 -0.382273  0.000000
C     1.832639 -0.319327  0.000000
O     2.982417 -0.232082  0.000000
C    -0.065785 -1.769675  1.242922
O    -0.078972 -2.654839  1.984478
C    -0.065785 -1.769675 -1.242922
O    -0.078972 -2.654839 -1.984478
C     0.449469  2.704125 -0.664897
C     0.240104  1.492522 -1.488779
C    -0.957322  0.804268 -1.654088
C    -1.992225  0.503654 -0.702763
C    -1.992225  0.503654  0.702763
C    -0.957322  0.804268  1.654088
C     0.240104  1.492522  1.488779
C     0.449469  2.704125  0.664897
H     0.601263  3.634446 -1.217038
H     0.926800  1.391698 -2.330651
H    -1.091674  0.282646 -2.601922
H    -2.852253 -0.005862 -1.135668
H    -2.852253 -0.005862  1.135668
H    -1.091674  0.282646  2.601922
H     0.926800  1.391698  2.330651
H     0.601263  3.634446  1.217038
    el energy= -735.924800456
          zpe= -735.763519
    th energy= -735.749249
  th enthalpy= -735.748305
  free energy= -735.804530

TS-1
Cr    0.341122 -0.000005 -0.006211
C     1.131231 -1.261507  1.112532
O     1.607233 -2.057546  1.800677
C     1.901723  0.000226 -1.042564
O     2.852010  0.000335 -1.695059
C     1.131330  1.260829  1.113173
O     1.607434  2.056539  1.801622
C    -2.723069  0.662594  0.682656
C    -1.463656  1.417833  0.461387
C    -0.927607  1.659633 -0.824552
C    -0.743194  0.715972 -1.865330
C    -0.743246 -0.715054 -1.865643
C    -0.927835 -1.659164 -0.825310
C    -1.463951 -1.418014  0.460687
C    -2.723194 -0.662656  0.682376
```

```
H    -3.640489  1.244758  0.802449
H    -1.298383  2.235334  1.165472
H    -0.458756  2.625188 -1.017295
H    -0.307644  1.135064 -2.771661
H    -0.307746 -1.133750 -2.772180
H    -0.459050 -2.624666 -1.018472
H    -1.298774 -2.235903  1.164341
H    -3.640708 -1.244699  0.802004
    el energy= -735.903610497
          zpe= -735.743141
    th energy= -735.729690
  th enthalpy= -735.728746
  free energy= -735.782650
```

TS-2

```
Cr   -0.025772  0.470278  0.000000
C     1.181707  1.044266  1.338225
O     1.920429  1.407140  2.142091
C     1.181707  1.044266 -1.338225
O     1.920429  1.407140 -2.142091
C    -0.799430  2.122385  0.000000
O    -1.311520  3.160367  0.000000
C     0.053350 -1.978102  1.668835
C    -0.905449 -0.865136  1.631605
C    -1.877479 -0.441307  0.716322
C    -1.877479 -0.441307 -0.716322
C    -0.905449 -0.865136 -1.631605
C     0.053350 -1.978102 -1.668835
C     0.652325 -2.751122 -0.730938
C     0.652325 -2.751122  0.730938
H     0.369110 -2.189996  2.690421
H    -1.086016 -0.470028  2.630013
H    -2.645989  0.201911  1.142481
H    -2.645989  0.201911 -1.142481
H    -1.086016 -0.470028 -2.630013
H     0.369110 -2.189996 -2.690421
H     1.326381 -3.502558 -1.141791
H     1.326381 -3.502558  1.141791
    el energy= -735.909824703
          zpe= -735.748805
    th energy= -735.734868
  th enthalpy= -735.733924
  free energy= -735.790018
```

TS-3

```
Cr   -0.001775 -0.492233  0.000000
C     1.902869 -0.455781  0.000000
O     3.050207 -0.403907  0.000000
C    -0.094506 -1.869757  1.247442
O    -0.148853 -2.742660  2.001289
C    -0.094506 -1.869757 -1.247442
O    -0.148853 -2.742660 -2.001289
C    -2.052299  0.321442  0.000000
C    -1.451614  0.530001  1.260883
C    -0.295906  1.268778  1.666631
C     0.267675  2.495541  1.240222
C     0.372565  3.124963  0.000000
C     0.267675  2.495541 -1.240222
C    -0.295906  1.268778 -1.666631
C    -1.451614  0.530001 -1.260883
H    -2.953733 -0.287221  0.000000
H    -1.948908  0.006921  2.077570
H    -0.001970  1.009183  2.683288
H     0.791306  3.020636  2.043697
H     0.848878  4.102648  0.000000
H     0.791306  3.020636 -2.043697
H    -0.001970  1.009183 -2.683288
H    -1.948908  0.006921 -2.077570
    el energy= -735.905142409
          zpe= -735.745941
    th energy= -735.731683
  th enthalpy= -735.730739
  free energy= -735.787480
```

TS-4

```
Cr   -0.048095  0.485602  0.000000
C    -1.624576  1.496059  0.000000
O    -2.581303  2.142538  0.000000
C     0.631175  1.605856  1.273791
O     1.064493  2.302625  2.093391
C     0.631175  1.605856 -1.273791
O     1.064493  2.302625 -2.093391
C    -1.498344 -1.695306 -0.678574
C    -0.457789 -2.180671 -1.600996
C     0.850909 -1.854802 -1.607239
C     1.583630 -0.960843 -0.693606
C     1.583630 -0.960843  0.693606
C     0.850909 -1.854802  1.607239
C    -0.457789 -2.180671  1.600996
C    -1.498344 -1.695306  0.678574
H    -2.417018 -1.361196 -1.164387
H    -0.830506 -2.785332 -2.429317
H     1.444720 -2.219361 -2.447643
H     2.405445 -0.426073 -1.167082
H     2.405445 -0.426073  1.167082
H     1.444720 -2.219361  2.447643
H    -0.830506 -2.785332  2.429317
H    -2.417018 -1.361196  1.164387
    el energy= -735.895186163
          zpe= -735.735587
    th energy= -735.721229
  th enthalpy= -735.720285
  free energy= -735.777570
```

TS-5

```
Cr    0.407284  0.000281 -0.283105
C     1.408959 -1.543744 -0.553286
O     2.067980 -2.455405 -0.807352
C     1.435640  0.000869  1.245006
O     2.085939  0.001333  2.203274
C     1.405875  1.546019 -0.552970
O     2.062961  2.459089 -0.807108
C    -1.304727  1.293382 -1.167309
C    -1.269130 -0.001208 -1.733014
C    -1.303501 -1.295775 -1.167416
C    -1.924049 -1.790642  0.076975
C    -1.698881 -1.257905  1.287107
C    -0.912060 -0.000272  1.496544
C    -1.701710  1.255669  1.286677
C    -1.927348  1.787622  0.076410
H    -1.082105  2.079890 -1.888600
H    -0.857651 -0.001067 -2.747373
```

```
H     -1.079995 -2.082191 -1.888548
H     -2.507490 -2.708625 -0.009598
H     -2.106822 -1.745889  2.174831
H     -0.505444  0.000392  2.511786
H     -2.111672  1.742465  2.174129
H     -2.513093  2.704061 -0.010981
    el energy= -735.850711911
          zpe= -735.692071
    th energy= -735.677972
  th enthalpy= -735.677028
  free energy= -735.733629
```

TS-6

```
Cr     0.770221 -0.039960 -0.271463
C      0.334336  0.149373  1.520382
O      0.089121  0.189063  2.646979
C      1.626305 -1.650657  0.222483
O      2.121593 -2.628095  0.575895
C      2.255779  1.030721 -0.090786
O      3.176480  1.730454 -0.052516
C     -2.008754  1.633406  0.441961
C     -0.763170  1.767395 -0.339997
C     -0.385107  1.201158 -1.563663
C     -0.533652 -0.161358 -2.006025
C     -1.020795 -1.312062 -1.385244
C     -2.023482 -1.588907 -0.362741
C     -2.814631 -0.811784  0.422505
C     -2.865155  0.616739  0.695179
H     -2.283376  2.581522  0.906724
H     -0.286153  2.730224 -0.161690
H      0.265314  1.805459 -2.194522
H      0.076396 -0.378338 -2.887186
H     -0.709358 -2.219208 -1.901046
H     -2.191453 -2.660179 -0.251174
H     -3.539823 -1.373058  1.012362
H     -3.724438  0.897126  1.304440
    el energy= -735.893854093
```

2

```
Cr    -0.875553  0.000033 -0.304650
C     -0.184098 -0.000025  1.409215
O      0.232186  0.000032  2.484271
C     -2.009346  1.425034  0.142342
O     -2.676839  2.326929  0.409551
C     -2.009994 -1.424474  0.142151
O     -2.677856 -2.326127  0.409267
C      2.034607 -1.663496  0.043727
C      0.883018 -1.630800 -0.858883
C      0.433302 -0.722114 -1.817720
C      0.433412  0.721329 -1.817999
C      0.883440  1.630420 -0.859707
C      2.034831  1.663298  0.043087
C      2.877753  0.726626  0.547517
C      2.877693 -0.726773  0.547749
H      2.266229 -2.684222  0.349721
H      0.476162 -2.628785 -1.018201
H     -0.200522 -1.155691 -2.595440
H     -0.200430  1.154615 -2.595877
H      0.476726  2.628378 -1.019513
H      2.266478  2.684105  0.348803
H      3.690494  1.140884  1.144132
H      3.690502 -1.140900  1.144364
    el energy= -735.896316253
          zpe= -735.735743
    th energy= -735.720950
  th enthalpy= -735.720006
  free energy= -735.778222
```

TS-7

```
Cr     0.318661  0.012708 -0.021700
C      1.849663 -1.078847  0.129143
O      2.823619 -1.687016  0.207458
C      1.643609  1.319164 -0.154432
O      2.507891  2.083500 -0.231089
C      0.142255 -0.026245  1.847405
O     -0.027948 -0.085227  2.985157
C     -2.607066  0.574073  0.480494
C     -1.486483  1.530499  0.241620
C     -0.800639  1.645639 -0.996660
C     -0.927165  0.451001 -1.777487
C     -0.027340 -0.631596 -2.029848
C     -0.448639 -1.721019 -1.162125
C     -1.326390 -1.575774 -0.049334
C     -2.551945 -0.754597  0.264182
H     -3.551777  1.017987  0.800396
H     -1.409106  2.368861  0.933400
H     -0.181966  2.508404 -1.222255
H     -1.911944  0.038156 -1.597793
H      0.847821 -0.685478 -2.667711
H      0.125531 -2.646770 -1.191882
H     -1.288041 -2.456338  0.596325
H     -3.466041 -1.333666  0.420364
    el energy= -735.855943756
          zpe= -735.695164
    th energy= -735.681422
  th enthalpy= -735.680477
  free energy= -735.735298
```

3

```
Cr    -0.363077 -0.038963  0.008833
C     -0.112107  1.792578  0.389106
O      0.042413  2.907343  0.627057
C     -1.507364  0.337620 -1.413220
O     -2.227794  0.557320 -2.288030
C     -1.879418 -0.020881  1.084190
O     -2.823251 -0.029887  1.749166
C      0.560955 -1.476845  1.368364
C     -0.111287 -2.187772  0.290614
C      0.052310 -1.845979 -1.067442
C      1.290342 -1.087685 -1.199598
C      1.559833  0.251059 -1.451941
C      2.630783  0.727765 -0.553136
C      2.562629  0.328500  0.741468
C      1.414310 -0.332422  1.451301
H      0.106474 -1.711466  2.331378
H     -0.905092 -2.864334  0.600858
H     -0.589009 -2.232155 -1.853636
H      2.059569 -1.538673 -0.578366
H      1.142634  0.860212 -2.248379
H      3.456825  1.343829 -0.905338
H      3.357654  0.642205  1.420208
H      1.387937  0.041646  2.477490
```

```
    el energy= -735.890534470
          zpe= -735.728992
    th energy= -735.715338
  th enthalpy= -735.714394
  free energy= -735.769016

TS-8
Cr    1.235836  0.000514 -0.418289
C     3.037536  0.001662 -0.923446
O     4.148938  0.002545 -1.236316
C     1.592976  1.286686  0.834359
O     1.800183  2.100756  1.631464
C     1.593908 -1.288665  0.831032
O     1.802123 -2.104938  1.625613
C    -3.917275  0.673657 -0.212391
C    -2.891909  1.603792  0.276842
C    -1.568636  1.618254  0.030825
C    -0.808819  0.689278 -0.825494
C    -0.808551 -0.689265 -0.825043
C    -1.568377 -1.617915  0.031628
C    -2.891713 -1.603598  0.277353
C    -3.917216 -0.673823 -0.212224
H    -4.838459  1.161127 -0.539240
H    -3.285923  2.439921  0.859216
H    -0.992308  2.455181  0.426252
H    -0.274793  1.187028 -1.661785
H    -0.274122 -1.187278 -1.660900
H    -0.991997 -2.454534  0.427616
H    -3.285606 -2.439607  0.859996
H    -4.838350 -1.161452 -0.538945
    el energy= -735.879188426
          zpe= -735.720927
    th energy= -735.705606
  th enthalpy= -735.704662
  free energy= -735.764971

4
Cr   -1.046925 -0.207990 -0.316170
C    -2.583519 -1.207389  0.019484
O    -3.541265 -1.831999  0.191552
C    -0.781065  0.035990  1.476656
O    -0.593937  0.201510  2.606807
C    -2.023417  1.344409 -0.410547
O    -2.622719  2.335000 -0.452931
C     3.393973 -1.093910 -0.037783
C     2.102386 -1.786513 -0.048043
C     0.980295 -1.412613 -0.697564
C     0.708307 -0.212194 -1.502490
C     0.838959  1.106135 -1.116961
C     1.537113  1.660927  0.048839
C     2.711370  1.284010  0.599852
C     3.636792  0.205428  0.232110
H     4.261023 -1.743679 -0.170747
H     2.081792 -2.747840  0.469711
H     0.142446 -2.140567 -0.680301
H     0.246267 -0.395209 -2.482879
H     0.475841  1.851000 -1.825844
H     1.077906  2.565240  0.449254
H     3.088371  1.926008  1.398454
H     4.688739  0.495039  0.285700
    el energy= -735.885236129
          zpe= -735.726604
    th energy= -735.711320
  th enthalpy= -735.710376
  free energy= -735.770560
```

B3LYP/BS3

```
I
Cr   -0.034015 -0.378288  0.000000
C     1.848810 -0.338516  0.000000
O     2.994030 -0.277392  0.000000
C    -0.079040 -1.774986  1.243457
O    -0.102912 -2.662949  1.972985
C    -0.079040 -1.774986 -1.243457
O    -0.102912 -2.662949 -1.972985
C     0.463123  2.701962 -0.660579
C     0.235515  1.505227 -1.489244
C    -0.954914  0.818954 -1.647573
C    -1.981159  0.515159 -0.699479
C    -1.981159  0.515159  0.699479
C    -0.954914  0.818954  1.647573
C     0.235515  1.505227  1.489244
C     0.463123  2.701962  0.660579
H     0.634567  3.625625 -1.205487
H     0.911115  1.412145 -2.332814
H    -1.097027  0.314886 -2.596407
H    -2.840870  0.017612 -1.130865
H    -2.840870  0.017612  1.130865
H    -1.097027  0.314886  2.596407
H     0.911115  1.412145  2.332814
H     0.634567  3.625625  1.205487
    el energy= -1694.40027290
          zpe= -1694.239252
    th energy= -1694.225023
  th enthalpy= -1694.224079
  free energy= -1694.280321

TS-1
Cr   -0.010765 -0.336861  0.000000
C     1.111656 -1.138693  1.265502
O     1.796176 -1.622768  2.051621
C    -1.056502 -1.905543  0.000000
O    -1.698322 -2.855859  0.000000
C     1.111656 -1.138693 -1.265502
O     1.796176 -1.622768 -2.051621
C     0.699371  2.713250 -0.658101
C     0.459314  1.464471 -1.413867
C    -0.824398  0.939174 -1.651384
C    -1.858826  0.756486 -0.712153
C    -1.858826  0.756486  0.712153
C    -0.824398  0.939174  1.651384
C     0.459314  1.464471  1.413867
C     0.699371  2.713250  0.658101
H     0.836456  3.624074 -1.234854
H     1.155158  1.300575 -2.230245
H    -1.025377  0.492765 -2.618764
H    -2.766373  0.339007 -1.130388
H    -2.766373  0.339007  1.130388
H    -1.025377  0.492765  2.618764
H     1.155158  1.300575  2.230245
H     0.836456  3.624074  1.234854
```

el energy= -1694.37967640
zpe= -1694.219424
th energy= -1694.206054
th enthalpy= -1694.205110
free energy= -1694.258914

TS-2

Cr	0.033393	-0.473542	0.000000
C	-1.175890	-1.068746	1.343592
O	-1.904333	-1.448124	2.140536
C	-1.175890	-1.068746	-1.343592
O	-1.904333	-1.448124	-2.140536
C	0.823065	-2.125339	0.000000
O	1.336768	-3.156805	0.000000
C	-0.073686	1.985608	1.658650
C	0.895900	0.891819	1.622998
C	1.866696	0.476067	0.712125
C	1.866696	0.476067	-0.712125
C	0.895900	0.891819	-1.622998
C	-0.073686	1.985608	-1.658650
C	-0.675350	2.750799	-0.727054
C	-0.675350	2.750799	0.727054
H	-0.392055	2.191625	2.674065
H	1.085451	0.510745	2.618384
H	2.651019	-0.135576	1.139720
H	2.651019	-0.135576	-1.139720
H	1.085451	0.510745	-2.618384
H	-0.392055	2.191625	-2.674065
H	-1.352761	3.490664	-1.137102
H	-1.352761	3.490664	1.137102

el energy= -1694.38615001
zpe= -1694.225443
th energy= -1694.211541
th enthalpy= -1694.210597
free energy= -1694.266726

TS-3

Cr	-0.000802	-0.483822	0.000000
C	1.921263	-0.469347	0.000000
O	3.063093	-0.447516	0.000000
C	-0.104416	-1.872293	1.245942
O	-0.168085	-2.751537	1.983148
C	-0.104416	-1.872293	-1.245942
O	-0.168085	-2.751537	-1.983148
C	-2.047343	0.335571	0.000000
C	-1.457962	0.550816	1.257022
C	-0.311760	1.286981	1.668379
C	0.282235	2.487653	1.236508
C	0.411143	3.103621	0.000000
C	0.282235	2.487653	-1.236508
C	-0.311760	1.286981	-1.668379
C	-1.457962	0.550816	-1.257022
H	-2.950820	-0.258736	0.000000
H	-1.962567	0.042184	2.069438
H	-0.032696	1.035387	2.684781
H	0.812238	3.000785	2.034703
H	0.913203	4.061506	0.000000
H	0.812238	3.000785	-2.034703
H	-0.032696	1.035387	-2.684781
H	-1.962567	0.042184	-2.069438

el energy= -1694.38174046
zpe= -1694.222806
th energy= -1694.208581
th enthalpy= -1694.207637
free energy= -1694.264403

TS-4

Cr	-0.047402	0.486276	0.000000
C	-1.628844	1.500719	0.000000
O	-2.577757	2.149034	0.000000
C	0.633581	1.616721	1.271819
O	1.065177	2.321841	2.077800
C	0.633581	1.616721	-1.271819
O	1.065177	2.321841	-2.077800
C	-1.490859	-1.702756	-0.674308
C	-0.457275	-2.182065	-1.596569
C	0.844319	-1.862963	-1.602642
C	1.578875	-0.981296	-0.689989
C	1.578875	-0.981296	0.689989
C	0.844319	-1.862963	1.602642
C	-0.457275	-2.182065	1.596569
C	-1.490859	-1.702756	0.674308
H	-2.408403	-1.380100	-1.155836
H	-0.830877	-2.775549	-2.424185
H	1.431234	-2.221206	-2.442314
H	2.401161	-0.457328	-1.160062
H	2.401161	-0.457328	1.160062
H	1.431234	-2.221206	2.442314
H	-0.830877	-2.775549	2.424185
H	-2.408403	-1.380100	1.155836

el energy= -1694.36933926
zpe= -1694.210053
th energy= -1694.195726
th enthalpy= -1694.194782
free energy= -1694.252119

TS-5

Cr	0.291505	0.407711	0.000000
C	0.562595	1.429376	1.541391
O	0.797388	2.085923	2.452658
C	-1.243284	1.438605	0.000000
O	-2.197460	2.085203	0.000000
C	0.562595	1.429376	-1.541391
O	0.797388	2.085923	-2.452658
C	1.162852	-1.327895	-1.289829
C	1.720142	-1.293120	0.000000
C	1.162852	-1.327895	1.289829
C	-0.084310	-1.925359	1.787277
C	-1.285489	-1.702019	1.255483
C	-1.494593	-0.924385	0.000000
C	-1.285489	-1.702019	-1.255483
C	-0.084310	-1.925359	-1.787277
H	1.885554	-1.120936	-2.070031
H	2.741399	-0.916700	0.000000
H	1.885554	-1.120936	2.070031
H	-0.004828	-2.497716	2.705308
H	-2.169488	-2.095219	1.747711
H	-2.499885	-0.512870	0.000000
H	-2.169488	-2.095219	-1.747711
H	-0.004828	-2.497716	-2.705308

el energy= -1694.32779678
zpe= -1694.169431

```
    th energy= -1694.155391
  th enthalpy= -1694.154447
```

B3LYP/BS4

```
I
Cr   -0.036354 -0.374770  0.000000
C     1.838430 -0.339988  0.000000
O     2.992106 -0.282039  0.000000
C    -0.078574 -1.768834  1.240402
O    -0.098502 -2.662069  1.975804
C    -0.078574 -1.768834 -1.240402
O    -0.098502 -2.662069 -1.975804
C     0.465987  2.706175 -0.665259
C     0.246162  1.497008 -1.489011
C    -0.954929  0.814447 -1.655044
C    -1.990049  0.516449 -0.703167
C    -1.990049  0.516449  0.703167
C    -0.954929  0.814447  1.655044
C     0.246162  1.497008  1.489011
C     0.465987  2.706175  0.665259
H     0.628940  3.637754 -1.215773
H     0.933303  1.394145 -2.332687
H    -1.096165  0.302256 -2.608970
H    -2.857110  0.016284 -1.137136
H    -2.857110  0.016284  1.137136
H    -1.096165  0.302256  2.608970
H     0.933303  1.394145  2.332687
H     0.628940  3.637754  1.215773
    el energy= -736.034420863
          zpe= -735.873915
    th energy= -735.859657
  th enthalpy= -735.858713
  free energy= -735.914916
```

```
TS-1
Cr    0.335402  0.000000  0.010538
C     1.136467  1.259857 -1.108913
O     1.626560  2.048746 -1.799301
C     1.898818  0.000046  1.052904
O     2.855410  0.000088  1.699756
C     1.136457 -1.259964 -1.108796
O     1.626561 -2.048921 -1.799098
C    -2.720064 -0.663030 -0.698444
C    -1.461914 -1.416360 -0.465358
C    -0.933746 -1.659549  0.824563
C    -0.756241 -0.715766  1.866087
C    -0.756232  0.715912  1.866037
C    -0.933731  1.659619  0.824440
C    -1.461904  1.416330 -0.465459
C    -2.720060  0.662991 -0.698485
H    -3.638524 -1.244467 -0.831008
H    -1.294083 -2.235276 -1.170113
H    -0.476616 -2.631260  1.023997
H    -0.333296 -1.136441  2.779782
H    -0.333281  1.136646  2.779701
H    -0.476589  2.631341  1.023797
H    -1.294072  2.235191 -1.170279
H    -3.638517  1.244424 -0.831086
    el energy= -736.013725065
          zpe= -735.854048
    th energy= -735.840616
  th enthalpy= -735.839672
```

```
  free energy= -1694.210816
```

```
  free energy= -735.893538
```

```
TS-2
Cr    0.031299 -0.467937  0.000000
C    -1.178536 -1.056240  1.337171
O    -1.916271 -1.435046  2.137196
C    -1.178536 -1.056240 -1.337171
O    -1.916271 -1.435046 -2.137196
C     0.811767 -2.120634  0.000000
O     1.324816 -3.160965  0.000000
C    -0.064806  1.982354  1.667714
C     0.896190  0.870917  1.629042
C     1.877206  0.457996  0.716000
C     1.877206  0.457996 -0.716000
C     0.896190  0.870917 -1.629042
C    -0.064806  1.982354 -1.667714
C    -0.664711  2.757702 -0.730845
C    -0.664711  2.757702  0.730845
H    -0.380036  2.196627  2.691250
H     1.082783  0.482365  2.630969
H     2.661533 -0.166575  1.145881
H     2.661533 -0.166575 -1.145881
H     1.082783  0.482365 -2.630969
H    -0.380036  2.196627 -2.691250
H    -1.336323  3.512583 -1.144904
H    -1.336323  3.512583  1.144904
    el energy= -736.019470285
          zpe= -735.859178
    th energy= -735.845259
  th enthalpy= -735.844315
  free energy= -735.900362
```

```
TS-3
Cr   -0.003146 -0.486280  0.000000
C     1.908042 -0.483942  0.000000
O     3.058122 -0.466038  0.000000
C    -0.109126 -1.868860  1.245963
O    -0.171382 -2.748224  1.995315
C    -0.109126 -1.868860 -1.245963
O    -0.171382 -2.748224 -1.995315
C    -2.051096  0.337419  0.000000
C    -1.450680  0.546652  1.261003
C    -0.286827  1.276326  1.663825
C     0.282060  2.501318  1.241115
C     0.393227  3.130170  0.000000
C     0.282060  2.501318 -1.241115
C    -0.286827  1.276326 -1.663825
C    -1.450680  0.546652 -1.261003
H    -2.963723 -0.257503  0.000000
H    -1.956911  0.035321  2.081723
H     0.003638  1.017997  2.683962
H     0.809682  3.024008  2.046109
H     0.877389  4.106341  0.000000
H     0.809682  3.024008 -2.046109
H     0.003638  1.017997 -2.683962
H    -1.956911  0.035321 -2.081723
    el energy= -736.015295215
          zpe= -735.856839
```

```
    th energy= -735.842594
  th enthalpy= -735.841650
  free energy= -735.898334

TS-4
Cr   -0.049301  0.486301  0.000000
C    -1.619351  1.509616  0.000000
O    -2.571983  2.166545  0.000000
C     0.638783  1.607598  1.272548
O     1.076489  2.310390  2.088392
C     0.638783  1.607598 -1.272548
O     1.076489  2.310390 -2.088392
C    -1.501885 -1.681768 -0.678857
C    -0.467767 -2.187659 -1.596680
C     0.844313 -1.873912 -1.602435
C     1.577777 -0.976107 -0.693901
C     1.577777 -0.976107  0.693901
C     0.844313 -1.873912  1.602435
C    -0.467767 -2.187659  1.596680
C    -1.501885 -1.681768  0.678857
H    -2.419258 -1.340364 -1.166427
H    -0.844463 -2.800709 -2.419782
H     1.437899 -2.254506 -2.438646
H     2.404180 -0.447097 -1.170038
H     2.404180 -0.447097  1.170038
H     1.437899 -2.254506  2.438646
H    -0.844463 -2.800709  2.419782
H    -2.419258 -1.340364  1.166427
    el energy= -736.004312890
          zpe= -735.845717
    th energy= -735.831323
  th enthalpy= -735.830379
```

B3LYP/BS5

```
I
Cr   -0.036286 -0.377801  0.000000
C     1.836001 -0.324725  0.000000
O     2.989074 -0.253195  0.000000
C    -0.071009 -1.768168  1.240984
O    -0.085214 -2.659920  1.978716
C    -0.071009 -1.768168 -1.240984
O    -0.085214 -2.659920 -1.978716
C     0.456922  2.704834 -0.665270
C     0.241325  1.493964 -1.487574
C    -0.958145  0.807966 -1.654410
C    -1.991929  0.506354 -0.703310
C    -1.991929  0.506354  0.703310
C    -0.958145  0.807966  1.654410
C     0.241325  1.493964  1.487574
C     0.456922  2.704834  0.665270
H     0.616798  3.636456 -1.216446
H     0.928302  1.393623 -2.331771
H    -1.098550  0.297765 -2.609554
H    -2.856688  0.002376 -1.137542
H    -2.856688  0.002376  1.137542
H    -1.098550  0.297765  2.609554
H     0.928302  1.393623  2.331771
H     0.616798  3.636456  1.216446
    el energy= -736.718929497
          zpe= -736.558388
    th energy= -736.544131
  th enthalpy= -736.543187
```

```
  free energy= -735.887810

TS-5
Cr    0.403858  0.000108 -0.289282
C     1.420441 -1.538102 -0.557339
O     2.085699 -2.451279 -0.797935
C     1.432551  0.000023  1.241322
O     2.090446  0.000037  2.197188
C     1.419619  1.538853 -0.556939
O     2.084350  2.452456 -0.797388
C    -1.317249  1.295200 -1.166956
C    -1.281219 -0.000494 -1.730755
C    -1.317083 -1.296052 -1.166664
C    -1.930508 -1.791233  0.080955
C    -1.702337 -1.256195  1.290156
C    -0.912052  0.000073  1.495076
C    -1.703040  1.255883  1.289709
C    -1.931270  1.790481  0.080359
H    -1.100710  2.082962 -1.891232
H    -0.887325 -0.000618 -2.754100
H    -1.100474 -2.083948 -1.890774
H    -2.517260 -2.710056  0.000225
H    -2.106660 -1.746862  2.180278
H    -0.504585  0.000379  2.511052
H    -2.107974  1.746370  2.179655
H    -2.518685  2.708844 -0.000800
    el energy= -735.961736393
          zpe= -735.803886
    th energy= -735.789802
  th enthalpy= -735.788858
  free energy= -735.845289


  free energy= -736.599390

TS-1
Cr    0.336736  0.000002  0.009221
C     1.131758  1.259754 -1.111388
O     1.616904  2.049823 -1.804115
C     1.898888  0.000014  1.049669
O     2.855326  0.000030  1.696941
C     1.131744 -1.259802 -1.111337
O     1.616889 -2.049906 -1.804024
C    -2.720683 -0.663030 -0.692014
C    -1.460848 -1.414787 -0.462240
C    -0.930027 -1.658807  0.826759
C    -0.747735 -0.715734  1.867460
C    -0.747728  0.715803  1.867439
C    -0.930013  1.658843  0.826705
C    -1.460837  1.414776 -0.462283
C    -2.720679  0.663019 -0.692028
H    -3.638817 -1.244912 -0.823525
H    -1.294243 -2.233773 -1.167045
H    -0.474346 -2.631443  1.025131
H    -0.321446 -1.136535  2.779460
H    -0.321434  1.136625  2.779427
H    -0.474323  2.631482  1.025042
H    -1.294232  2.233736 -1.167118
H    -3.638810  1.244904 -0.823551
    el energy= -736.698685959
          zpe= -736.538928
```

```
    th energy= -736.525512
  th enthalpy= -736.524568
  free energy= -736.578398
```

TS-2

```
Cr    0.034120 -0.475107  0.000000
C    -1.180884 -1.049919  1.334400
O    -1.923931 -1.418362  2.134541
C    -1.180884 -1.049919 -1.334400
O    -1.923931 -1.418362 -2.134541
C     0.805998 -2.131095  0.000000
O     1.313852 -3.174000  0.000000
C    -0.059847  1.984421  1.666971
C     0.894873  0.867073  1.627495
C     1.876696  0.451229  0.715574
C     1.876696  0.451229 -0.715574
C     0.894873  0.867073 -1.627495
C    -0.059847  1.984421 -1.666971
C    -0.653598  2.765375 -0.730622
C    -0.653598  2.765375  0.730622
H    -0.374385  2.198809  2.690618
H     1.079780  0.478810  2.629856
H     2.660436 -0.173143  1.146689
H     2.660436 -0.173143 -1.146689
H     1.079780  0.478810 -2.629856
H    -0.374385  2.198809 -2.690618
H    -1.320654  3.523919 -1.145260
H    -1.320654  3.523919  1.145260
    el energy= -736.704324308
          zpe= -736.544072
    th energy= -736.530137
  th enthalpy= -736.529193
  free energy= -736.585308
```

TS-3

```
Cr   -0.002355 -0.488756  0.000000
C     1.906119 -0.471710  0.000000
O     3.056070 -0.443191  0.000000
C    -0.102871 -1.868005  1.246821
O    -0.163190 -2.746517  1.997524
C    -0.102871 -1.868005 -1.246821
O    -0.163190 -2.746517 -1.997524
C    -2.052117  0.329244  0.000000
C    -1.451718  0.539329  1.260557
C    -0.289008  1.272378  1.661536
C     0.275793  2.500114  1.240604
C     0.384150  3.130175  0.000000
C     0.275793  2.500114 -1.240604
C    -0.289008  1.272378 -1.661536
C    -1.451718  0.539329 -1.260557
H    -2.963413 -0.267648  0.000000
H    -1.956509  0.028431  2.082358
H     0.004855  1.012568  2.680439
H     0.802539  3.022880  2.046021
H     0.865377  4.107775  0.000000
H     0.802539  3.022880 -2.046021
H     0.004855  1.012568 -2.680439
H    -1.956509  0.028431 -2.082358
    el energy= -736.700295450
          zpe= -736.541839
    th energy= -736.527580
  th enthalpy= -736.526636
  free energy= -736.583390
```

TS-4

```
Cr   -0.048050  0.490680  0.000000
C    -1.617899  1.509494  0.000000
O    -2.572781  2.163626  0.000000
C     0.640321  1.606120  1.275147
O     1.079020  2.303646  2.095229
C     0.640321  1.606120 -1.275147
O     1.079020  2.303646 -2.095229
C    -1.500832 -1.675690 -0.679092
C    -0.469900 -2.188249 -1.596449
C     0.842570 -1.876366 -1.602369
C     1.573730 -0.976139 -0.694468
C     1.573730 -0.976139  0.694468
C     0.842570 -1.876366  1.602369
C    -0.469900 -2.188249  1.596449
C    -1.500832 -1.675690  0.679092
H    -2.416306 -1.328973 -1.166479
H    -0.848831 -2.801373 -2.418349
H     1.437264 -2.258569 -2.436852
H     2.401804 -0.449451 -1.170422
H     2.401804 -0.449451  1.170422
H     1.437264 -2.258569  2.436852
H    -0.848831 -2.801373  2.418349
H    -2.416306 -1.328973  1.166479
    el energy= -736.688676857
          zpe= -736.530048
    th energy= -736.515656
  th enthalpy= -736.514711
  free energy= -736.572125
```

TS-5

```
Cr    0.409457  0.000217 -0.289458
C     1.425675 -1.535964 -0.552887
O     2.088867 -2.451363 -0.791104
C     1.424454 -0.000005  1.248430
O     2.072569 -0.000063  2.211111
C     1.424584  1.537084 -0.552215
O     2.087101  2.453048 -0.790215
C    -1.309098  1.295786 -1.169860
C    -1.271034 -0.000686 -1.732451
C    -1.308903 -1.296965 -1.169521
C    -1.931384 -1.793377  0.073488
C    -1.712495 -1.256410  1.283252
C    -0.921159  0.000116  1.488141
C    -1.713663  1.255890  1.282629
C    -1.932658  1.792244  0.072670
H    -1.089354  2.082701 -1.894047
H    -0.877024 -0.000843 -2.756015
H    -1.089008 -2.084039 -1.893483
H    -2.518037 -2.711643 -0.012333
H    -2.124023 -1.743596  2.171856
H    -0.517055  0.000571  2.505440
H    -2.126244  1.742656  2.170978
H    -2.520439  2.709731 -0.013801
    el energy= -736.647320934
          zpe= -736.489463
    th energy= -736.475376
  th enthalpy= -736.474432
```

```
  free energy= -736.530891
```

B97-1/BS2

```
I
Cr   -0.038992 -0.369002  0.000000
C     1.839242 -0.311717  0.000000
O     2.983930 -0.231529  0.000000
C    -0.068125 -1.768103  1.241783
O    -0.076850 -2.656716  1.970363
C    -0.068125 -1.768103 -1.241783
O    -0.076850 -2.656716 -1.970363
C     0.449549  2.694287 -0.661995
C     0.242305  1.479747 -1.478665
C    -0.958557  0.802036 -1.651419
C    -1.990093  0.501094 -0.702380
C    -1.990093  0.501094  0.702380
C    -0.958557  0.802036  1.651419
C     0.242305  1.479747  1.478665
C     0.449549  2.694287  0.661995
H     0.598902  3.620220 -1.213613
H     0.926728  1.378580 -2.317213
H    -1.097593  0.292502 -2.600744
H    -2.849255 -0.002658 -1.133178
H    -2.849255 -0.002658  1.133178
H    -1.097593  0.292502  2.600744
H     0.926728  1.378580  2.317213
H     0.598902  3.620220  1.213613
    el energy= -735.952274029
          zpe= -735.791612
    th energy= -735.777451
  th enthalpy= -735.776506
  free energy= -735.832561

TS-1
Cr   -0.012677 -0.326144  0.000000
C     1.112355 -1.127188  1.262536
O     1.800469 -1.610597  2.045678
C    -1.051983 -1.896204  0.000000
O    -1.689569 -2.849489  0.000000
C     1.112355 -1.127188 -1.262536
O     1.800469 -1.610597 -2.045678
C     0.688429  2.710429 -0.659833
C     0.465446  1.446778 -1.404795
C    -0.824638  0.925004 -1.656599
C    -1.862146  0.743123 -0.715378
C    -1.862146  0.743123  0.715378
C    -0.824638  0.925004  1.656599
C     0.465446  1.446778  1.404795
C     0.688429  2.710429  0.659833
H     0.812092  3.621667 -1.242543
H     1.166707  1.282792 -2.219980
H    -1.023805  0.474569 -2.625050
H    -2.769069  0.317165 -1.132448
H    -2.769069  0.317165  1.132448
H    -1.023805  0.474569  2.625050
H     1.166707  1.282792  2.219980
H     0.812092  3.621667  1.242543
    el energy= -735.932422381
          zpe= -735.772405
    th energy= -735.759124
  th enthalpy= -735.758180
  free energy= -735.811790

TS-2
Cr    0.034257 -0.451314  0.000000
C    -1.181779 -1.042223  1.334958
O    -1.910646 -1.428018  2.128728
C    -1.181779 -1.042223 -1.334958
O    -1.910646 -1.428018 -2.128728
C     0.808568 -2.116820  0.000000
O     1.307959 -3.154110  0.000000
C    -0.065875  1.965750  1.663648
C     0.905641  0.867922  1.627450
C     1.882784  0.455737  0.714260
C     1.882784  0.455737 -0.714260
C     0.905641  0.867922 -1.627450
C    -0.065875  1.965750 -1.663648
C    -0.674015  2.727973 -0.729150
C    -0.674015  2.727973  0.729150
H    -0.384326  2.173533  2.681101
H     1.094058  0.482497  2.623736
H     2.662668 -0.166074  1.141242
H     2.662668 -0.166074 -1.141242
H     1.094058  0.482497 -2.623736
H    -0.384326  2.173533 -2.681101
H    -1.356398  3.465902 -1.140137
H    -1.356398  3.465902  1.140137
    el energy= -735.936108899
          zpe= -735.775819
    th energy= -735.761924
  th enthalpy= -735.760980
  free energy= -735.817108

TS-3
Cr   -0.004281 -0.471909  0.000000
C     1.913936 -0.464229  0.000000
O     3.056188 -0.449525  0.000000
C    -0.099774 -1.860493  1.247136
O    -0.157730 -2.738846  1.985175
C    -0.099774 -1.860493 -1.247136
O    -0.157730 -2.738846 -1.985175
C    -2.058415  0.325131  0.000000
C    -1.461654  0.536356  1.258588
C    -0.305328  1.270924  1.665367
C     0.283118  2.479320  1.237402
C     0.405198  3.103240  0.000000
C     0.283118  2.479320 -1.237402
C    -0.305328  1.270924 -1.665367
C    -1.461654  0.536356 -1.258588
H    -2.962059 -0.272633  0.000000
H    -1.964207  0.026143  2.074465
H    -0.018716  1.011449  2.680170
H     0.819382  2.989303  2.036805
H     0.905402  4.064260  0.000000
H     0.819382  2.989303 -2.036805
H    -0.018716  1.011449 -2.680170
H    -1.964207  0.026143 -2.074465
    el energy= -735.931273438
          zpe= -735.772810
    th energy= -735.758573
  th enthalpy= -735.757629
```

```
  free energy= -735.814455
```

```
TS-4
Cr   -0.052467  0.463225  0.000000
C    -1.616556  1.505425  0.000000
O    -2.540862  2.188293  0.000000
C     0.632218  1.597024  1.271753
O     1.063512  2.309327  2.070854
C     0.632218  1.597024 -1.271753
O     1.063512  2.309327 -2.070854
C    -1.498012 -1.691091 -0.676293
C    -0.461312 -2.175921 -1.598316
C     0.843318 -1.853119 -1.604724
C     1.575560 -0.962299 -0.693062
C     1.575560 -0.962299  0.693062
C     0.843318 -1.853119  1.604724
C    -0.461312 -2.175921  1.598316
C    -1.498012 -1.691091  0.676293
H    -2.415484 -1.362477 -1.159292
H    -0.834128 -2.773998 -2.425775
H     1.431869 -2.214020 -2.444998
H     2.401738 -0.439842 -1.163462
H     2.401738 -0.439842  1.163462
H     1.431869 -2.214020  2.444998
H    -0.834128 -2.773998  2.425775
H    -2.415484 -1.362477  1.159292
    el energy= -735.919438863
          zpe= -735.760586
    th energy= -735.746238
  th enthalpy= -735.745294
  free energy= -735.802882
```

```
TS-5
Cr    0.314527  0.404906  0.000000
C     0.566566  1.419701  1.549758
O     0.774584  2.071981  2.469633
C    -1.218898  1.429405  0.000000
O    -2.168586  2.083046  0.000000
C     0.566566  1.419701 -1.549758
O     0.774584  2.071981 -2.469633
C     1.152800 -1.327652 -1.293372
C     1.715731 -1.309360  0.000000
C     1.152800 -1.327652  1.293372
C    -0.102917 -1.923768  1.787689
C    -1.301453 -1.679430  1.253096
C    -1.486298 -0.876672  0.000000
C    -1.301453 -1.679430 -1.253096
C    -0.102917 -1.923768 -1.787689
H     1.879401 -1.127421 -2.075344
H     2.741805 -0.937380  0.000000
H     1.879401 -1.127421  2.075344
H    -0.027631 -2.519254  2.693967
H    -2.194173 -2.084076  1.724888
H    -2.493470 -0.461377  0.000000
H    -2.194173 -2.084076 -1.724888
H    -0.027631 -2.519254 -2.693967
    el energy= -735.878795739
          zpe= -735.720729
    th energy= -735.706713
  th enthalpy= -735.705769
  free energy= -735.761911
```

B97-1/BS3

```
I
Cr    0.039971  0.364415  0.000000
C    -1.832378  0.295517  0.000000
O    -2.978120  0.211929  0.000000
C     0.063260  1.764261  1.230069
O     0.067905  2.661702  1.950982
C     0.063260  1.764261 -1.230069
O     0.067905  2.661702 -1.950982
C    -0.436903 -2.695320 -0.661614
C    -0.245475 -1.471322 -1.469427
C     0.956488 -0.793244 -1.650581
C     1.987722 -0.493980 -0.702916
C     1.987722 -0.493980  0.702916
C     0.956488 -0.793244  1.650581
C    -0.245475 -1.471322  1.469427
C    -0.436903 -2.695320  0.661614
H    -0.569045 -3.621482 -1.217280
H    -0.934717 -1.370077 -2.304176
H     1.095291 -0.286522 -2.601416
H     2.844643  0.014858 -1.132707
H     2.844643  0.014858  1.132707
H     1.095291 -0.286522  2.601416
H    -0.934717 -1.370077  2.304176
H    -0.569045 -3.621482  1.217280
    el energy= -1694.10660525
          zpe= -1693.945839
    th energy= -1693.931761
  th enthalpy= -1693.930817
  free energy= -1693.986681
```

```
TS-1
Cr   -0.016701 -0.321932  0.000000
C     1.104750 -1.124931  1.253956
O     1.793949 -1.615879  2.034042
C    -1.048323 -1.890433  0.000000
O    -1.678449 -2.850527  0.000000
C     1.104750 -1.124931 -1.253956
O     1.793949 -1.615879 -2.034042
C     0.686141  2.714199 -0.659569
C     0.471594  1.442451 -1.394392
C    -0.819246  0.922150 -1.654697
C    -1.858037  0.742064 -0.715436
C    -1.858037  0.742064  0.715436
C    -0.819246  0.922150  1.654697
C     0.471594  1.442451  1.394392
C     0.686141  2.714199  0.659569
H     0.800739  3.623996 -1.246310
H     1.177842  1.273944 -2.204605
H    -1.017805  0.475832 -2.625186
H    -2.764406  0.314248 -1.132249
H    -2.764406  0.314248  1.132249
H    -1.017805  0.475832  2.625186
H     1.177842  1.273944  2.204605
H     0.800739  3.623996  1.246310
    el energy= -1694.08719667
          zpe= -1693.927120
    th energy= -1693.913894
  th enthalpy= -1693.912950
```

free energy= -1693.966451

TS-2

Cr	0.036832	-0.455918	0.000000
C	-1.170502	-1.050324	1.331244
O	-1.898097	-1.441046	2.126273
C	-1.170502	-1.050324	-1.331244
O	-1.898097	-1.441046	-2.126273
C	0.803392	-2.110995	0.000000
O	1.302418	-3.151589	0.000000
C	-0.071023	1.973593	1.662494
C	0.893830	0.869309	1.624466
C	1.875524	0.458860	0.713669
C	1.875524	0.458860	-0.713669
C	0.893830	0.869309	-1.624466
C	-0.071023	1.973593	-1.662494
C	-0.673131	2.741106	-0.729167
C	-0.673131	2.741106	0.729167
H	-0.389524	2.179814	2.680250
H	1.079401	0.482444	2.620870
H	2.655552	-0.162249	1.141635
H	2.655552	-0.162249	-1.141635
H	1.079401	0.482444	-2.620870
H	-0.389524	2.179814	-2.680250
H	-1.350677	3.483459	-1.140316
H	-1.350677	3.483459	1.140316

el energy= -1694.08940965
zpe= -1693.929066
th energy= -1693.915209
th enthalpy= -1693.914265
free energy= -1693.970322

TS-3

Cr	-0.002889	-0.471521	0.000000
C	1.910513	-0.461363	0.000000
O	3.054369	-0.449975	0.000000
C	-0.099484	-1.859286	1.232500
O	-0.160045	-2.747577	1.962348
C	-0.099484	-1.859286	-1.232500
O	-0.160045	-2.747577	-1.962348
C	-2.052293	0.320791	0.000000
C	-1.456177	0.533775	1.258500
C	-0.300985	1.271725	1.661644
C	0.277736	2.486315	1.236323
C	0.394583	3.112029	0.000000
C	0.277736	2.486315	-1.236323
C	-0.300985	1.271725	-1.661644
C	-1.456177	0.533775	-1.258500
H	-2.952367	-0.282713	0.000000
H	-1.955524	0.021185	2.074830
H	-0.010116	1.012276	2.675322
H	0.810345	2.998848	2.036560
H	0.888149	4.076531	0.000000
H	0.810345	2.998848	-2.036560
H	-0.010116	1.012276	-2.675322
H	-1.955524	0.021185	-2.074830

el energy= -1694.08508337
zpe= -1693.926505
th energy= -1693.912320
th enthalpy= -1693.911376
free energy= -1693.968123

TS-4

Cr	0.054040	-0.461316	0.000000
C	1.616258	-1.491710	0.000000
O	2.547053	-2.168688	0.000000
C	-0.624765	-1.597806	1.257742
O	-1.058980	-2.315302	2.053847
C	-0.624765	-1.597806	-1.257742
O	-1.058980	-2.315302	-2.053847
C	1.492181	1.692637	-0.676397
C	0.455654	2.173221	-1.600241
C	-0.848207	1.848405	-1.606105
C	-1.577354	0.956533	-0.693227
C	-1.577354	0.956533	0.693227
C	-0.848207	1.848405	1.606105
C	0.455654	2.173221	1.600241
C	1.492181	1.692637	0.676397
H	2.410980	1.366455	-1.158426
H	0.828440	2.768803	-2.429536
H	-1.437391	2.204951	-2.447841
H	-2.400710	0.429937	-1.164174
H	-2.400710	0.429937	1.164174
H	-1.437391	2.204951	2.447841
H	0.828440	2.768803	2.429536
H	2.410980	1.366455	1.158426

el energy= -1694.07114528
zpe= -1693.912207
th energy= -1693.897902
th enthalpy= -1693.896957
free energy= -1693.954437

TS-5

Cr	0.316115	0.400117	0.000000
C	0.561382	1.412549	1.540620
O	0.769225	2.064400	2.464304
C	-1.198907	1.441945	0.000000
O	-2.142870	2.107532	0.000000
C	0.561382	1.412549	-1.540620
O	0.769225	2.064400	-2.464304
C	1.146494	-1.325013	-1.292809
C	1.711020	-1.310281	0.000000
C	1.146494	-1.325013	1.292809
C	-0.107603	-1.925827	1.786471
C	-1.304642	-1.679507	1.250976
C	-1.483910	-0.872007	0.000000
C	-1.304642	-1.679507	-1.250976
C	-0.107603	-1.925827	-1.786471
H	1.871740	-1.121277	-2.075164
H	2.735819	-0.934285	0.000000
H	1.871740	-1.121277	2.075164
H	-0.031266	-2.523664	2.690967
H	-2.199451	-2.084606	1.718314
H	-2.486039	-0.444458	0.000000
H	-2.199451	-2.084606	-1.718314
H	-0.031266	-2.523664	-2.690967

el energy= -1694.03350669
zpe= -1693.875316
th energy= -1693.861397
th enthalpy= -1693.860453
free energy= -1693.916280

Molecular Coordinates for (TMCOT)Cr$(CO)_3$ (Å)

PBE0/BS1

```
II
C     2.536410  0.095897 -0.033772
C     1.436369  1.005236 -0.447383
C     0.629885  1.704174  0.467701
C    -0.016077  1.121367  1.607909
C    -0.347127 -0.205973  1.954110
C    -0.050450 -1.429564  1.255388
C     0.902653 -1.727650  0.285608
C     2.266687 -1.159078  0.306592
Cr   -0.596720  0.055258 -0.249506
C    -0.138524 -0.514084 -1.929636
C    -2.182585 -0.877961 -0.257590
C    -1.670453  1.373412 -0.934524
O     0.208143 -0.886999 -2.964154
O    -3.192919 -1.433478 -0.283883
O    -2.390130  2.151490 -1.390379
C     3.910982  0.697922 -0.002866
C     0.343599  3.169642  0.230233
C    -1.315618 -0.371507  3.105563
C     0.797510 -3.065007 -0.408864
H     3.954835  1.540872  0.701309
H     4.185730  1.097747 -0.989454
H     4.668722 -0.036800  0.291370
H     1.478553 -3.792371  0.058414
H     1.091381 -2.982947 -1.462329
H    -0.220326 -3.468928 -0.370689
H    -2.118636 -1.073166  2.850421
H    -1.775719  0.577697  3.397490
H    -0.792089 -0.778644  3.981428
H    -0.639279  3.477900  0.603277
H     0.394932  3.410082 -0.837247
H     1.106968  3.775033  0.739838
H     3.058645 -1.839810  0.630812
H    -0.782970 -2.212697  1.461554
H    -0.567888  1.851675  2.199983
H     1.658102  1.579116 -1.351535
    el energy= -892.224976056
          zpe= -891.949782
    th energy= -891.929554
  th enthalpy= -891.928610
  free energy= -891.996913

TS-A
C    -2.592326  0.043452 -0.254779
C    -1.451513 -0.833917 -0.634508
C    -0.803093 -1.624133  0.359589
C    -0.258544 -1.054911  1.536996
C     0.106865  0.283473  1.905299
C    -0.060219  1.498650  1.183685
C    -0.916646  1.782515  0.102530
C    -2.312289  1.291620  0.089931
Cr    0.597783 -0.035409 -0.235078
C     0.878146 -1.027079 -1.747466
C     1.477417  1.263038 -1.181496
C     2.244342 -0.579484  0.375305
O     1.036444 -1.665537 -2.695391
O     2.055109  2.058312 -1.786623
O     3.266432 -0.933831  0.772435
C    -3.959515 -0.573380 -0.245807
C    -0.592778 -3.102891  0.142639
C     0.959700  0.377142  3.152946
C    -0.742614  3.115219 -0.587445
H    -4.008467 -1.409699  0.466221
H    -4.211882 -0.985655 -1.233107
H    -4.731220  0.154906  0.027828
H    -1.485838  3.830138 -0.201318
H    -0.917016  3.025379 -1.665983
H     0.252930  3.544431 -0.437157
H     0.311760  0.434796  4.038462
H     1.615582 -0.491713  3.275236
H     1.584821  1.276054  3.144454
H    -1.484739 -3.657984  0.467026
H    -0.446834 -3.320229 -0.921385
H     0.273190 -3.489721  0.691405
H    -3.081998  2.002275  0.405062
H     0.659418  2.270659  1.462711
H     0.213627 -1.790813  2.188841
H    -1.615608 -1.370440 -1.574313
    el energy= -892.204187881
          zpe= -891.929758
    th energy= -891.910224
  th enthalpy= -891.909280
  free energy= -891.975636

TS-B
C    -0.026757  2.201392  1.231760
C    -0.184448  2.834822  0.000000
C    -0.026757  2.201392 -1.231760
C    -0.157248  0.818047 -1.546812
C    -1.274475 -0.054202 -1.262036
C    -1.858438 -0.296024  0.000000
C    -1.274475 -0.054202  1.262036
C    -0.157248  0.818047  1.546812
Cr    0.268776 -0.797800  0.000000
C     2.086315 -0.426321  0.000000
C     0.450651 -2.155562  1.209893
C     0.450651 -2.155562 -1.209893
O     3.201200 -0.145433  0.000000
O     0.578948 -3.034516  1.949411
O     0.578948 -3.034516 -1.949411
C     0.418398  3.065448  2.380878
C     0.418398  3.065448 -2.380878
C    -1.914107 -0.725748 -2.453388
C    -1.914107 -0.725748  2.453388
H     0.620566  4.098510  2.081956
H     1.329639  2.646106  2.829028
H    -0.343647  3.069320  3.172745
H    -2.612134 -0.032586  2.944456
H    -1.161771 -1.014207  3.195858
H    -2.469641 -1.625037  2.164051
```

```
H    -2.469641 -1.625037 -2.164051
H    -2.612134 -0.032586 -2.944456
H    -1.161771 -1.014207 -3.195858
H    -0.343647  3.069320 -3.172745
H     0.620566  4.098510 -2.081956
H     1.329639  2.646106 -2.829028
H     0.263653  0.590069  2.528631
H    -2.699161 -0.988110  0.000000
H     0.263653  0.590069 -2.528631
H    -0.095658  3.919312  0.000000
    el energy= -892.203247978
          zpe= -891.929558
    th energy= -891.909679
  th enthalpy= -891.908734
  free energy= -891.976785
```

TS-C

```
C     1.580892  1.686035  0.258653
C     0.215079  1.628120  0.816565
C    -0.477713  0.770357  1.691344
C    -0.457368 -0.660234  1.722405
C     0.223644 -1.620610  0.957524
C     1.607155 -1.648583  0.455806
C     2.543839 -0.737496  0.103386
C     2.500455  0.726408  0.009399
Cr   -0.688690 -0.003353 -0.315531
C    -0.826508  1.233049 -1.695366
C    -0.838282 -1.387385 -1.543710
C    -2.477367  0.006395 -0.165402
O    -0.934402  2.003416 -2.542176
O    -0.944821 -2.250022 -2.296641
O    -3.628317  0.010050 -0.049067
C     1.932003  3.100770 -0.138251
C    -1.567717  1.404718  2.528647
C    -0.339966 -3.027517  1.069597
C     3.880288 -1.272684 -0.354733
H     1.874520  3.777702  0.725981
H     1.216303  3.477252 -0.883885
H     2.936568  3.172176 -0.566460
H     4.691072 -0.863692  0.265439
H     4.091923 -0.964871 -1.388981
H     3.930879 -2.364775 -0.305785
H     0.196343 -3.592878  1.845624
H    -1.405266 -3.019083  1.320622
H    -0.222749 -3.575635  0.127134
H    -1.176518  1.630160  3.530726
H    -1.914885  2.345867  2.089295
H    -2.435151  0.746212  2.649423
H     3.425133  1.117133 -0.416595
H     1.930142 -2.679879  0.307893
H    -1.312949 -1.065151  2.263232
H    -0.227096  2.624312  0.816956
    el energy= -892.196067018
          zpe= -891.921207
    th energy= -891.901404
  th enthalpy= -891.900460
  free energy= -891.968284
```

TS-D

```
C    -1.824772 -0.822530 -1.349770
C    -0.631007 -1.440567 -0.716371
C    -0.432155 -1.611168  0.651830
C    -1.165495 -0.880133  1.690327
C    -1.436206  0.443924  1.777572
C    -1.086370  1.471193  0.777429
C    -1.422083  1.517277 -0.539840
C    -2.182134  0.465605 -1.236780
Cr    0.811929  0.141105 -0.156152
C     1.893923 -0.600616 -1.381883
C     1.823724  1.671743 -0.294077
C     1.986625 -0.402519  1.094104
O     2.573424 -1.092789 -2.182426
O     2.497804  2.603707 -0.391301
O     2.735973 -0.739682  1.911293
C    -2.619080 -1.775038 -2.200357
C     0.303204 -2.836468  1.142010
C    -2.041101  1.014013  3.032264
C    -1.142233  2.751310 -1.357299
H    -2.967020 -2.630592 -1.605235
H    -1.993674 -2.185772 -3.005648
H    -3.489023 -1.288853 -2.654855
H    -2.090913  3.206264 -1.678215
H    -0.585704  2.505286 -2.270275
H    -0.574677  3.498463 -0.793089
H    -2.985157  1.531744  2.813185
H    -2.236636  0.236183  3.777890
H    -1.369888  1.759549  3.482611
H    -0.424345 -3.625723  1.387035
H     0.975595 -3.225781  0.370491
H     0.894998 -2.639524  2.042642
H    -3.060765  0.814725 -1.784129
H    -0.624527  2.367310  1.199183
H    -1.398682 -1.475924  2.576426
H    -0.162547 -2.199930 -1.345868
    el energy= -892.188172977
          zpe= -891.914867
    th energy= -891.894566
  th enthalpy= -891.893621
  free energy= -891.963048
```

TS-E

```
C     0.884883 -1.629468  0.192299
C     2.224749 -1.218249 -0.009715
C     2.895376 -0.000126  0.080720
C     2.224670  1.218067 -0.008403
C     0.884887  1.629115  0.194239
C     0.026338  1.245624  1.281259
C    -0.247018 -0.001217  1.890877
C     0.026484 -1.247340  1.279967
Cr   -0.769222 -0.000045 -0.226553
C    -2.151218 -1.196182 -0.317052
C    -2.151030  1.196384 -0.315529
C    -0.479573  0.001803 -2.078724
O    -3.050250 -1.918310 -0.376557
O    -3.049922  1.918760 -0.374164
O    -0.273920  0.003124 -3.207078
C     0.585567 -3.019946 -0.356012
C     4.399673  0.000016 -0.037075
C     0.585528  3.020190 -0.352507
C    -1.135792 -0.002020  3.110266
H     1.068841 -3.803135  0.248693
H     0.942841 -3.127335 -1.386481
```

```
H    -0.489179 -3.227673 -0.366670
H    -0.531970 -0.003796  4.028042
H    -1.779843 -0.888648  3.134973
H    -1.778204  0.885713  3.137353
H     1.068893  3.802684  0.253012
H     0.942688  3.128732 -1.382893
H    -0.489213  3.227953 -0.362811
H     4.881471  0.000252  0.950751
H     4.764013 -0.887826 -0.569795
H     4.763841  0.887726 -0.570137
H    -0.537773 -2.079606  1.708507
H    -0.537929  2.077355  1.710795
H     2.862147  2.029831 -0.379683
H     2.862307 -2.029651 -0.381658
    el energy= -892.185219784
          zpe= -891.913395
    th energy= -891.892696
  th enthalpy= -891.891752
  free energy= -891.962076

TS-F
C     0.195152  1.303588 -1.516431
C     0.589509 -0.000295 -1.902516
C     0.183712 -1.301525 -1.519302
C    -1.155107 -1.739575 -1.073947
C    -1.819852 -1.231960 -0.026630
C    -1.319747  0.005179  0.669695
C    -1.809407  1.247603 -0.024718
C    -1.140090  1.751846 -1.070726
Cr    0.785286 -0.003662  0.254282
C     1.615529  1.528754  0.795533
C     0.487754 -0.004194  2.039654
C     1.601734 -1.544320  0.793131
O     2.205406  2.467875  1.110960
O     0.313782 -0.004652  3.184463
O     2.182840 -2.489282  1.107403
C     1.033240  2.442579 -2.064396
C     1.011989 -2.446587 -2.069599
C    -3.089841 -1.848358  0.483847
C    -3.074940  1.872930  0.485980
H     2.059733  2.131668 -2.290137
H     1.080182  3.281836 -1.360874
H     0.587233  2.823761 -2.995586
H    -3.351143  2.759152 -0.096931
H    -2.957384  2.177870  1.536043
H    -3.918624  1.169407  0.457198
H    -3.928318 -1.138590  0.456089
H    -3.372812 -2.731948 -0.099802
H    -2.974165 -2.155169  1.533575
H     0.562775 -2.822003 -3.001587
H     2.041141 -2.144077 -2.294648
H     1.051623 -3.287647 -1.367778
H    -1.527852  2.640831 -1.574073
H    -1.720217  0.006234  1.690786
H    -1.550111 -2.624660 -1.578523
H     1.597041 -0.004299 -2.336577
    el energy= -892.146807420
          zpe= -891.873611
    th energy= -891.853765
  th enthalpy= -891.852821
  free energy= -891.920639
```

PBEPBE/auto/BS1

```
II
C     2.558381  0.131923 -0.079889
C     1.431314  1.025845 -0.483668
C     0.623703  1.730889  0.446958
C     0.008837  1.140956  1.612852
C    -0.295740 -0.201070  1.978810
C    -0.000650 -1.432027  1.271973
C     0.957477 -1.736742  0.289486
C     2.314373 -1.136960  0.276743
Cr   -0.610811  0.048842 -0.241843
C    -0.184405 -0.529210 -1.931142
C    -2.193244 -0.891681 -0.224401
C    -1.734349  1.333777 -0.909564
O     0.132619 -0.914654 -2.989539
O    -3.222096 -1.451896 -0.247988
O    -2.501404  2.096765 -1.361355
C     3.931510  0.758520 -0.079917
C     0.317717  3.200471  0.205321
C    -1.236736 -0.373981  3.163168
C     0.858218 -3.087294 -0.397231
H     3.978799  1.615669  0.620259
H     4.184474  1.159144 -1.081489
H     4.712665  0.033671  0.207745
H     1.584370 -3.799084  0.045217
H     1.106946 -3.002200 -1.470660
H    -0.152804 -3.521985 -0.314398
H    -2.044599 -1.090696  2.930329
H    -1.701714  0.577966  3.468689
H    -0.681422 -0.777303  4.031790
H    -0.663974  3.505384  0.606783
H     0.334499  3.432803 -0.873296
H     1.095841  3.823298  0.689335
H     3.134480 -1.806733  0.582719
H    -0.720807 -2.230951  1.503363
H    -0.544035  1.871783  2.219172
H     1.625363  1.594814 -1.407262
    el energy= -892.262116341
          zpe= -891.996490
    th energy= -891.975575
  th enthalpy= -891.974630
  free energy= -892.044549

TS-A
C    -2.613263  0.004616 -0.321206
C    -1.444914 -0.866674 -0.664273
C    -0.804708 -1.650319  0.356778
C    -0.295804 -1.058850  1.552196
C     0.036457  0.300190  1.919963
C    -0.127020  1.513717  1.173299
C    -0.975214  1.799346  0.069339
C    -2.363350  1.276323  0.008856
Cr    0.614787 -0.036388 -0.220210
C     0.984893 -1.048985 -1.700807
C     1.489501  1.257445 -1.179224
C     2.267772 -0.514899  0.429509
O     1.222885 -1.702167 -2.644057
O     2.080338  2.057260 -1.800802
```

```
O     3.317197 -0.832866  0.839304
C    -3.977480 -0.638724 -0.333437
C    -0.574199 -3.137091  0.156516
C     0.850529  0.423747  3.201003
C    -0.788767  3.138066 -0.621747
H    -4.030260 -1.471772  0.395177
H    -4.203532 -1.076613 -1.325572
H    -4.774583  0.084015 -0.086791
H    -1.554280  3.854608 -0.258449
H    -0.932225  3.045340 -1.712768
H     0.206998  3.575698 -0.442231
H     0.167534  0.477060  4.070865
H     1.523318 -0.437388  3.354724
H     1.461982  1.341754  3.204716
H    -1.478533 -3.703099  0.455660
H    -0.388454 -3.361270 -0.908379
H     0.281922 -3.516531  0.741004
H    -3.165145  1.983866  0.278139
H     0.578162  2.304489  1.469417
H     0.169540 -1.786412  2.231720
H    -1.567300 -1.405692 -1.618352
    el energy= -892.243386222
          zpe= -891.978555
    th energy= -891.958304
  th enthalpy= -891.957360
  free energy= -892.025422
```

TS-B

```
C    -0.013843  2.233859  1.243549
C    -0.155261  2.869280  0.000000
C    -0.013843  2.233859 -1.243549
C    -0.163900  0.841985 -1.560995
C    -1.287799 -0.035073 -1.274302
C    -1.871821 -0.281064  0.000000
C    -1.287799 -0.035073  1.274302
C    -0.163900  0.841985  1.560995
Cr    0.270961 -0.807375  0.000000
C     2.093925 -0.457344  0.000000
C     0.438856 -2.183192  1.190692
C     0.438856 -2.183192 -1.190692
O     3.233039 -0.201151  0.000000
O     0.561997 -3.093507  1.922840
O     0.561997 -3.093507 -1.922840
C     0.436443  3.099313  2.401175
C     0.436443  3.099313 -2.401175
C    -1.931337 -0.712004 -2.471708
C    -1.931337 -0.712004  2.471708
H     0.667007  4.133906  2.098016
H     1.337936  2.661693  2.871807
H    -0.339878  3.127931  3.190844
H    -2.629597 -0.013537  2.973853
H    -1.172115 -1.010751  3.215161
H    -2.496780 -1.613575  2.178195
H    -2.496780 -1.613575 -2.178195
H    -2.629597 -0.013537 -2.973853
H    -1.172115 -1.010751 -3.215161
H    -0.339878  3.127931 -3.190844
H     0.667007  4.133906 -2.098016
H     1.337936  2.661693 -2.871807
H     0.269132  0.602131  2.544274
H    -2.718991 -0.978649  0.000000
H     0.269132  0.602131 -2.544274
H    -0.053827  3.961478  0.000000
    el energy= -892.245859145
          zpe= -891.981232
    th energy= -891.960778
  th enthalpy= -891.959834
  free energy= -892.029126
```

TS-C

```
C     1.576459  1.722250  0.243140
C     0.231684  1.622375  0.855217
C    -0.429294  0.720540  1.729926
C    -0.380300 -0.720339  1.718723
C     0.328082 -1.659685  0.931776
C     1.696635 -1.638188  0.387834
C     2.612185 -0.685414  0.030559
C     2.524666  0.781311 -0.045358
Cr   -0.708904 -0.004143 -0.310506
C    -0.957424  1.267638 -1.637254
C    -0.913132 -1.374823 -1.542820
C    -2.486406 -0.033993 -0.107937
O    -1.150551  2.069840 -2.464971
O    -1.066152 -2.241771 -2.311383
O    -3.654103 -0.053462  0.034376
C     1.871521  3.153483 -0.173634
C    -1.504639  1.315519  2.627940
C    -0.205928 -3.089337  1.019850
C     3.958017 -1.185273 -0.466334
H     1.822182  3.841296  0.693217
H     1.112258  3.505448 -0.900462
H     2.864401  3.255451 -0.641670
H     4.779089 -0.765792  0.148405
H     4.146016 -0.849841 -1.505557
H     4.035918 -2.284564 -0.439835
H     0.381840 -3.672827  1.755851
H    -1.266160 -3.116220  1.320401
H    -0.117317 -3.605758  0.047342
H    -2.364451  0.635442  2.757632
H    -1.080361  1.516816  3.631128
H    -1.882438  2.271912  2.228281
H     3.429461  1.206815 -0.501552
H     2.043544 -2.664104  0.199394
H    -1.217918 -1.162137  2.275255
H    -0.238683  2.614250  0.890380
    el energy= -892.235458690
          zpe= -891.970046
    th energy= -891.949575
  th enthalpy= -891.948631
  free energy= -892.018088
```

TS-D

```
C    -1.859301 -0.786930 -1.373056
C    -0.655835 -1.431964 -0.767088
C    -0.449583 -1.661794  0.607114
C    -1.145988 -0.949922  1.687270
C    -1.398785  0.389661  1.828786
C    -1.081105  1.451150  0.848140
C    -1.440284  1.539238 -0.477452
C    -2.211819  0.509957 -1.207105
Cr    0.798621  0.145433 -0.173969
C     1.918013 -0.577116 -1.373598
```

```
C     1.799662  1.679155 -0.317564
C     1.979784 -0.393207  1.065960
O     2.642257 -1.056093 -2.170294
O     2.480827  2.627603 -0.428260
O     2.747936 -0.749008  1.884321
C    -2.663450 -1.705502 -2.264908
C     0.302523 -2.907400  1.044611
C    -1.930526  0.921057  3.142682
C    -1.187086  2.817832 -1.251161
H    -3.021089 -2.589002 -1.701459
H    -2.035089 -2.095404 -3.089773
H    -3.535470 -1.193748 -2.706657
H    -2.152995  3.280937 -1.536570
H    -0.643646  2.616938 -2.192474
H    -0.609886  3.551392 -0.663894
H    -2.890963  1.452570  2.997816
H    -2.084935  0.114736  3.879374
H    -1.227912  1.657892  3.579967
H    -0.421937 -3.706496  1.304184
H     0.948091 -3.285729  0.234434
H     0.934916 -2.729759  1.931822
H    -3.095502  0.885530 -1.746406
H    -0.625273  2.349591  1.292275
H    -1.338831 -1.566513  2.579509
H    -0.191856 -2.178645 -1.428821
    el energy= -892.226613058
          zpe= -891.962625
    th energy= -891.941714
  th enthalpy= -891.940770
  free energy= -892.011671
```

TS-E

```
C     0.914012 -1.649231  0.194455
C     2.261758 -1.230613 -0.021612
C     2.932241  0.000134  0.055340
C     2.261668  1.230836 -0.020190
C     0.913724  1.648955  0.196235
C     0.051909  1.257771  1.287519
C    -0.223533 -0.001132  1.899929
C     0.051968 -1.259248  1.285989
Cr   -0.784344  0.000158 -0.229907
C    -2.182187 -1.173775 -0.297052
C    -2.182300  1.174003 -0.296247
C    -0.521679  0.001265 -2.086215
O    -3.112600 -1.888444 -0.344750
O    -3.112639  1.888750 -0.343748
O    -0.347129  0.002074 -3.238124
C     0.603036 -3.042362 -0.363315
C     4.444517  0.000169 -0.072039
C     0.602618  3.042715 -0.360029
C    -1.104601 -0.002181  3.135257
H     1.057665 -3.838249  0.261567
H     0.992051 -3.158951 -1.389646
H    -0.482983 -3.228621 -0.403824
H    -0.485817 -0.005739  4.053257
H    -1.753803 -0.894775  3.167361
H    -1.749905  0.893044  3.171690
H     0.991019  3.160233 -1.386486
H    -0.483393  3.229184 -0.399722
H     1.057771  3.837933  0.265329
H     4.809035 -0.893350 -0.611684
H     4.809289  0.895997 -0.607641
H     4.935226 -0.002148  0.920766
H    -0.520529 -2.094960  1.718110
H    -0.520607  2.092920  1.720693
H     2.904555  2.051520 -0.387272
H     2.904721 -2.050701 -0.389898
    el energy= -892.230238929
          zpe= -891.967386
    th energy= -891.946100
  th enthalpy= -891.945156
  free energy= -892.016913
```

TS-F

```
C     0.154148  1.315713 -1.553447
C     0.565700  0.000440 -1.925259
C     0.155950 -1.315391 -1.553376
C    -1.170576 -1.763686 -1.073389
C    -1.836647 -1.257794 -0.006527
C    -1.372206 -0.000939  0.679910
C    -1.838157  1.255453 -0.006266
C    -1.172830  1.762275 -1.073182
Cr    0.753959  0.000424  0.252914
C     1.622375  1.519596  0.773155
C     0.555430  0.000208  2.063074
C     1.624126 -1.517678  0.773166
O     2.258183  2.456632  1.080277
O     0.428214 -0.000006  3.231710
O     2.260970 -2.454010  1.080320
C     0.987974  2.462206 -2.115975
C     0.991281 -2.460767 -2.115933
C    -3.067413 -1.927486  0.555148
C    -3.069317  1.923998  0.555906
H     2.011293  2.142598 -2.378104
H     1.065484  3.298406 -1.399202
H     0.512972  2.861064 -3.035288
H    -3.345213  2.823588 -0.021874
H    -2.900652  2.230116  1.607655
H    -3.940108  1.239533  0.565737
H    -3.939298 -1.244364  0.562885
H    -3.341231 -2.828334 -0.021659
H    -2.899576 -2.231784  1.607545
H     0.516929 -2.860074 -3.035385
H     2.014263 -2.139872 -2.377819
H     1.069688 -3.296964 -1.399258
H    -1.554086  2.683356 -1.541187
H    -1.742698 -0.001240  1.720398
H    -1.550664 -2.685142 -1.541598
H     1.569917  0.001122 -2.383286
    el energy= -892.193987396
          zpe= -891.930296
    th energy= -891.909788
  th enthalpy= -891.908844
  free energy= -891.978007
```

B3LYP/BS1

```
II
C     0.578212  1.719731  0.549062
C    -0.023827  1.051563  1.674651
C    -0.285753 -0.302932  1.959354
C     0.069163 -1.490628  1.205908
C     1.053771 -1.749549  0.267091
C     2.366203 -1.067144  0.223970
C     2.548882  0.214878 -0.087376
C     1.405992  1.117557 -0.411765
Cr   -0.646792  0.055748 -0.272289
C    -1.786560  1.383109 -0.875369
C    -0.219520 -0.450244 -2.004864
C    -2.202199 -0.968429 -0.256784
O    -2.537686  2.170128 -1.269703
O     0.108108 -0.788320 -3.060674
O    -3.184516 -1.578366 -0.256175
C     0.229054  3.192412  0.399264
C    -1.203542 -0.578721  3.141597
C     1.022981 -3.081159 -0.457951
C     3.910834  0.866905 -0.118768
H     0.960866  3.796520  0.956038
H    -0.768814  3.435729  0.779924
H     0.280237  3.503402 -0.649737
H     3.970298  1.692383  0.605536
H     4.113999  1.302666 -1.108090
H     4.708642  0.150799  0.109417
H     1.817384 -3.741525 -0.077623
H     0.061739 -3.593712 -0.341213
H     1.209770 -2.945282 -1.530764
H    -0.629404 -1.011859  3.973065
H    -1.691302  0.331354  3.505380
H    -1.985500 -1.300878  2.876956
H     1.593372  1.751101 -1.281876
H     3.223154 -1.703448  0.460657
H    -0.633940 -2.306419  1.377922
H    -0.576890  1.729582  2.323644
    el energy= -893.191300325
          zpe= -892.918704
    th energy= -892.898013
  th enthalpy= -892.897069
  free energy= -892.966648
```

```
TS-A
C    -2.599949 -0.057461 -0.294655
C    -1.433771 -0.940488 -0.609190
C    -0.767697 -1.661902  0.422409
C    -0.246854 -1.032610  1.584664
C     0.066373  0.329561  1.907410
C    -0.149757  1.526378  1.152789
C    -1.030637  1.805890  0.097449
C    -2.387282  1.216434  0.015791
Cr    0.645845 -0.035891 -0.259180
C     1.461162  1.309705 -1.236129
C     2.321255 -0.493434  0.401376
C     0.977852 -1.087719 -1.746829
O     1.977136  2.147332 -1.846323
O     3.348908 -0.794548  0.836398
O     1.158813 -1.762281 -2.668977
C    -3.960886 -0.709352 -0.318505
C    -0.510188 -3.149618  0.261962
C     0.887227  0.506644  3.177742
C    -0.902098  3.150643 -0.591808
H    -4.019891 -1.525236  0.416895
H    -4.166317 -1.157745 -1.301487
H    -4.759006  0.008769 -0.097105
H    -1.689181  3.830539 -0.228732
H    -1.042050  3.053166 -1.675106
H     0.068040  3.625099 -0.414791
H     0.216341  0.602031  4.043622
H     1.552763 -0.343284  3.364103
H     1.498816  1.414324  3.136879
H    -1.391547 -3.719024  0.592570
H    -0.339661 -3.402047 -0.790553
H     0.356017 -3.492165  0.839181
H    -3.212589  1.892836  0.256676
H     0.548838  2.322199  1.413844
H     0.226290 -1.730041  2.275215
H    -1.574511 -1.518080 -1.526507
    el energy= -893.169934552
          zpe= -892.898189
    th energy= -892.878188
  th enthalpy= -892.877244
  free energy= -892.944715
```

```
TS-B
C    -0.023863  2.217057  1.242410
C    -0.147530  2.846140  0.000000
C    -0.023863  2.217057 -1.242410
C    -0.204835  0.842264 -1.581016
C    -1.306158 -0.040552 -1.268716
C    -1.877345 -0.293543  0.000000
C    -1.306158 -0.040552  1.268716
C    -0.204835  0.842264  1.581016
Cr    0.286899 -0.819614  0.000000
C     2.126944 -0.438820  0.000000
C     0.465143 -2.183955  1.238903
C     0.465143 -2.183955 -1.238903
O     3.242822 -0.150501  0.000000
O     0.590438 -3.052885  1.994031
O     0.590438 -3.052885 -1.994031
C     0.440082  3.086978  2.392969
C     0.440082  3.086978 -2.392969
C    -1.961943 -0.721585 -2.457910
C    -1.961943 -0.721585  2.457910
H     0.691103  4.104647  2.078150
H     1.325662  2.641339  2.866859
H    -0.335102  3.141851  3.170793
H    -2.665713 -0.029612  2.943795
H    -1.219101 -1.011857  3.209123
H    -2.515476 -1.619599  2.160920
H    -2.515476 -1.619599 -2.160920
H    -2.665713 -0.029612 -2.943795
H    -1.219101 -1.011857 -3.209123
H    -0.335102  3.141851 -3.170793
H     0.691103  4.104647 -2.078150
H     1.325662  2.641339 -2.866859
H     0.195339  0.618504  2.571268
H    -2.720558 -0.981270  0.000000
H     0.195339  0.618504 -2.571268
H    -0.021577  3.926505  0.000000
```

```
    el energy= -893.174501287
          zpe= -892.903215
    th energy= -892.882966
  th enthalpy= -892.882022
  free energy= -892.950901

TS-C
C    -1.566071  1.716923 -0.268948
C    -0.215012  1.626545 -0.868021
C     0.454393  0.731158 -1.721701
C     0.404181 -0.705970 -1.725442
C    -0.306926 -1.648792 -0.970260
C    -1.676548 -1.631028 -0.423529
C    -2.583349 -0.689384 -0.058507
C    -2.502349  0.778129  0.015390
Cr    0.729878 -0.005937  0.334160
C     0.912117  1.272198  1.698066
C     0.859053 -1.397789  1.589378
C     2.538331 -0.033860  0.135242
O     1.032821  2.061083  2.530328
O     0.936054 -2.258268  2.352995
O     3.688256 -0.052175 -0.010113
C    -1.886253  3.152554  0.114423
C     1.535018  1.327299 -2.612982
C     0.210784 -3.081879 -1.086177
C    -3.928894 -1.190516  0.439259
H    -1.842289  3.814180 -0.762888
H    -1.147017  3.532635  0.834860
H    -2.878609  3.246553  0.566316
H    -4.745094 -0.772735 -0.168533
H    -4.111811 -0.864590  1.473821
H    -4.004294 -2.281902  0.406742
H    -0.391546 -3.645929 -1.813865
H     1.256665 -3.115081 -1.406615
H     0.135605 -3.608149 -0.126947
H     1.124888  1.508575 -3.617253
H     1.893763  2.286409 -2.224472
H     2.397846  0.660686 -2.722704
H    -3.408096  1.194141  0.455925
H    -2.022514 -2.650845 -0.255633
H     1.239887 -1.136084 -2.275281
H     0.237641  2.615984 -0.906152
    el energy= -893.169530211
          zpe= -892.897022
    th energy= -892.876881
  th enthalpy= -892.875937
  free energy= -892.944553

TS-D
C    -1.697599 -0.719456 -1.540725
C    -0.584084 -1.423523 -0.844601
C    -0.488826 -1.699573  0.513385
C    -1.317698 -1.070480  1.556094
C    -1.627143  0.234234  1.739898
C    -1.207306  1.363218  0.874239
C    -1.443261  1.549191 -0.453420
C    -2.084153  0.554376 -1.337420
Cr    0.878438  0.157869 -0.071210
C     1.990139 -0.400734 -1.398605
C     1.830253  1.751028  0.004333
C     2.049811 -0.526222  1.143405
O     2.677814 -0.763479 -2.262365
O     2.444445  2.731594  0.043215
O     2.782462 -0.952054  1.938327
C    -2.373533 -1.566212 -2.596955
C     0.240810 -2.953746  0.963225
C    -2.381077  0.674200  2.976187
C    -1.162564  2.889106 -1.102908
H    -2.791103 -2.482499 -2.156225
H    -1.647689 -1.886507 -3.358451
H    -3.181131 -1.023345 -3.100252
H    -2.107609  3.343256 -1.436987
H    -0.532416  2.778207 -1.994194
H    -0.672576  3.584301 -0.413697
H    -3.315226  1.187263  2.706423
H    -2.627351 -0.174258  3.623687
H    -1.786825  1.391189  3.561820
H    -0.488128 -3.765216  1.117475
H     0.960712 -3.286228  0.208451
H     0.777515 -2.810130  1.907201
H    -2.886089  0.951557 -1.963469
H    -0.806113  2.217905  1.423575
H    -1.626807 -1.756501  2.348293
H    -0.052080 -2.118783 -1.494822
    el energy= -893.165191791
          zpe= -892.894111
    th energy= -892.873513
  th enthalpy= -892.872569
  free energy= -892.942677

TS-E
C    -0.233088  0.910408  1.665850
C     0.015259  2.238076  1.229690
C    -0.041977  2.900650  0.000000
C     0.015259  2.238076 -1.229690
C    -0.233088  0.910408 -1.665850
C    -1.299621  0.039934 -1.253282
C    -1.901532 -0.239587  0.000000
C    -1.299621  0.039934  1.253282
Cr    0.245613 -0.797803  0.000000
C     0.329997 -2.185485  1.228215
C     0.329997 -2.185485 -1.228215
C     2.121620 -0.502778  0.000000
O     0.389189 -3.071713  1.969200
O     0.389189 -3.071713 -1.969200
O     3.251930 -0.292034  0.000000
C     0.276505  0.621764  3.082719
C     0.121487  4.410406  0.000000
C     0.276505  0.621764 -3.082719
C    -3.141768 -1.114353  0.000000
H    -0.376320  1.077143  3.844429
H     1.286888  1.019346  3.232028
H     0.319925 -0.452602  3.287758
H    -4.050980 -0.496407  0.000000
H    -3.179996 -1.756879  0.887594
H    -3.179996 -1.756879 -0.887594
H    -0.376320  1.077143 -3.844429
H     1.286888  1.019346 -3.232028
H     0.319925 -0.452602 -3.287758
H     0.665242  4.759135  0.887764
H     0.665242  4.759135 -0.887764
H    -0.851445  4.923236  0.000000
```

```
H    -1.734537 -0.531207  2.076242
H    -1.734537 -0.531207 -2.076242
H     0.393621  2.875942 -2.036346
H     0.393621  2.875942  2.036346
    el energy= -893.158036811
          zpe= -892.888629
    th energy= -892.867584
  th enthalpy= -892.866640
  free energy= -892.937776
```

TS-F

```
C    -0.126400 -1.308173 -1.547917
C    -0.533387 -0.000642 -1.914027
C    -0.131805  1.307932 -1.544406
C     1.191702  1.756078 -1.055132
C     1.840857  1.251768  0.007663
C     1.348444  0.002558  0.696386
C     1.841025 -1.248480  0.012518
C     1.194644 -1.754570 -1.051666
Cr   -0.797081  0.001824  0.283343
C    -1.675386 -1.564814  0.747473
C    -0.553346 -0.005798  2.106304
C    -1.669343  1.568277  0.756664
O    -2.307613 -2.497433  1.002335
O    -0.393384 -0.009572  3.253938
O    -2.299055  2.501494  1.015847
C    -0.939336 -2.452075 -2.144203
C    -0.948930  2.450069 -2.138449
C     3.092885  1.894958  0.552940
C     3.087847 -1.895328  0.565322
H    -1.955566 -2.142543 -2.413838
H    -1.015125 -3.299446 -1.452869
H    -0.451072 -2.823702 -3.058703
H     3.366313 -2.789906 -0.004414
H     2.937596 -2.192159  1.614096
H     3.943810 -1.205289  0.556066
H     3.946470  1.202000  0.539831
H     3.371285  2.787726 -0.019675
H     2.949509  2.193864  1.602075
H    -0.464334  2.822907 -3.054439
H    -1.965342  2.138309 -2.404965
H    -1.024564  3.297206 -1.446818
H     1.588472 -2.654508 -1.529567
H     1.721093  0.004724  1.726112
H     1.585632  2.652949 -1.538783
H    -1.520633 -0.002367 -2.388131
    el energy= -893.116737517
          zpe= -892.846219
    th energy= -892.825942
  th enthalpy= -892.824998
  free energy= -892.895198
```

Molecular Coordinates for (TMCOT)Mo$(CO)_3$ (Å)

PBE0/BS1

```
II
C     2.649382  0.131574 -0.268016
C     1.526790  1.066024 -0.545696
C     0.803913  1.740549  0.455026
C     0.289374  1.145081  1.668052
C     0.020426 -0.184350  2.070104
C     0.252580 -1.428175  1.362758
C     1.092251 -1.730556  0.290992
C     2.428959 -1.121141  0.119594
Mo   -0.605908  0.038026 -0.206581
C    -0.370064 -0.630275 -2.062628
C    -2.307861 -0.980591  0.001626
C    -1.837491  1.462237 -0.836504
O    -0.140036 -1.048705 -3.110600
O    -3.315113 -1.532149  0.108170
O    -2.601884  2.245284 -1.202361
C     4.024235  0.716908 -0.421222
C     0.467461  3.201889  0.261062
C    -0.766626 -0.343635  3.353256
C     0.951397 -3.096399 -0.341467
H     4.166384  1.569717  0.257696
H     4.174771  1.099145 -1.441074
H     4.805663 -0.022842 -0.214572
H     1.705429 -3.787629  0.065301
H     1.115200 -3.044829 -1.424437
H    -0.042089 -3.524612 -0.168133
H    -0.103412 -0.692235  4.157137
H    -1.224907  0.596407  3.675843
H    -1.562294 -1.088918  3.240559
H     1.246293  3.821857  0.728662
H     0.441421  3.454992 -0.804148
H    -0.497278  3.477051  0.701569
H     3.269484 -1.784588  0.342803
H    -0.428326 -2.220437  1.679605
H    -0.178527  1.876852  2.326927
H     1.668239  1.662684 -1.450646
    el energy= -873.521605073
          zpe= -873.248247
    th energy= -873.227411
  th enthalpy= -873.226467
  free energy= -873.296868

TS-A
C    -2.667131  0.104261 -0.466839
C    -1.518651 -0.808129 -0.742278
C    -1.012580 -1.640017  0.312968
C    -0.557516 -1.091934  1.538686
C    -0.226705  0.242424  1.998750
C    -0.311989  1.489439  1.321385
C    -0.991351  1.799640  0.108014
C    -2.391238  1.339490 -0.072482
Mo    0.597421 -0.022068 -0.196067
C     0.998218 -0.956014 -1.899193
C     1.734481  1.414746 -0.977389
C     2.270188 -0.813549  0.575031
O     1.195303 -1.534026 -2.877959
O     2.401703  2.236764 -1.434164
O     3.200891 -1.283704  1.061794
C    -4.044780 -0.474572 -0.602502
C    -0.857534 -3.126474  0.104319
C     0.470912  0.264724  3.343316
C    -0.731771  3.169851 -0.478191
H    -4.186729 -1.321090  0.084726
H    -4.210777 -0.862382 -1.617725
H    -4.819630  0.270734 -0.390915
H    -1.472279  3.891097 -0.095896
H    -0.832747  3.152174 -1.569177
H     0.266186  3.549688 -0.234822
H    -0.274210  0.203956  4.148722
H     1.160699 -0.577965  3.464365
H     1.036604  1.190843  3.485904
H    -0.593440 -3.340640 -0.937331
H    -0.086775 -3.564089  0.748617
H    -1.809991 -3.636419  0.309999
H    -3.176432  2.058075  0.182702
H     0.327110  2.263724  1.750932
H    -0.169471 -1.848855  2.221991
H    -1.608294 -1.337578 -1.696206
    el energy= -873.500378975
          zpe= -873.227731
    th energy= -873.207662
  th enthalpy= -873.206718
  free energy= -873.274824

TS-B
C     0.036655  2.373919  1.242803
C    -0.040157  3.003574  0.000000
C     0.036655  2.373919 -1.242803
C    -0.251134  1.028558 -1.605098
C    -1.379025  0.189008 -1.275601
C    -1.954158 -0.040506  0.000000
C    -1.379025  0.189008  1.275601
C    -0.251134  1.028558  1.605098
Mo    0.263842 -0.741689  0.000000
C     2.274794 -0.606359  0.000000
C     0.301634 -2.227538  1.289929
C     0.301634 -2.227538 -1.289929
O     3.417713 -0.485463  0.000000
O     0.305400 -3.124944  2.019476
O     0.305400 -3.124944 -2.019476
C     0.574549  3.209280  2.376549
C     0.574549  3.209280 -2.376549
C    -2.064999 -0.469687 -2.449663
C    -2.064999 -0.469687  2.449663
H     0.926885  4.191658  2.048151
H     1.407578  2.686698  2.865394
H    -0.198329  3.352222  3.144975
H    -2.755975  0.241391  2.924935
H    -1.336562 -0.777092  3.208007
H    -2.635111 -1.354549  2.145747
H    -2.635111 -1.354549 -2.145747
H    -2.755975  0.241391 -2.924935
```

```
H    -1.336562 -0.777092 -3.208007
H    -0.198329  3.352222 -3.144975
H     0.926885  4.191658 -2.048151
H     1.407578  2.686698 -2.865394
H     0.110597  0.798243  2.609166
H    -2.824246 -0.694817  0.000000
H     0.110597  0.798243 -2.609166
H     0.181593  4.068697  0.000000
    el energy= -873.500002766
          zpe= -873.227718
    th energy= -873.207234
  th enthalpy= -873.206290
  free energy= -873.276518
```

TS-C

```
C    -1.750179 -1.684788  0.247242
C    -0.475115 -1.636784  0.980690
C     0.179648 -0.738722  1.840949
C     0.163612  0.704345  1.839289
C    -0.473891  1.664862  1.042656
C    -1.775627  1.669633  0.362878
C    -2.654285  0.746508 -0.101376
C    -2.609915 -0.718160 -0.157226
Mo    0.636775 -0.006826 -0.299198
C     0.984755 -1.387751 -1.713054
C     1.067095  1.507210 -1.538707
C     2.513260 -0.067059  0.057245
O     1.210003 -2.202121 -2.493668
O     1.330580  2.405181 -2.208508
O     3.650183 -0.103967  0.287870
C    -2.068626 -3.102911 -0.167759
C     1.195123 -1.340929  2.788817
C     0.053971  3.080366  1.209458
C    -3.911492  1.274526 -0.752775
H    -2.151561 -3.759123  0.710109
H    -1.260383 -3.509082 -0.793108
H    -3.001734 -3.167676 -0.735490
H    -4.801679  0.881143 -0.240884
H    -3.980292  0.943532 -1.798966
H    -3.963103  2.367428 -0.735674
H    -0.599655  3.651051  1.885286
H     1.069013  3.093734  1.618103
H     0.072160  3.609702  0.249448
H     0.739405 -1.470872  3.780761
H     1.532135 -2.324013  2.444590
H     2.077254 -0.701929  2.907309
H    -3.464981 -1.110598 -0.707892
H    -2.075998  2.694731  0.143903
H     0.970736  1.114386  2.445630
H    -0.062003 -2.640534  1.077672
    el energy= -873.493675568
          zpe= -873.219863
    th energy= -873.199620
  th enthalpy= -873.198675
  free energy= -873.268243
```

TS-D

```
C    -1.790483 -0.672096 -1.603979
C    -0.733340 -1.426487 -0.885317
C    -0.685410 -1.700455  0.480212
C    -1.566009 -1.078475  1.483578
C    -1.867085  0.222367  1.684912
C    -1.380062  1.363269  0.878667
C    -1.539644  1.573401 -0.456611
C    -2.150406  0.607509 -1.386481
Mo    0.798014  0.152837 -0.027410
C     2.028103 -0.459992 -1.364147
C     1.916438  1.781679  0.141301
C     2.011780 -0.695826  1.184217
O     2.751412 -0.837516 -2.191398
O     2.589743  2.715466  0.226754
O     2.727809 -1.218310  1.935323
C    -2.435730 -1.460873 -2.711025
C    -0.007102 -2.966705  0.950088
C    -2.695054  0.643237  2.869053
C    -1.203057  2.911597 -1.063763
H    -2.893144 -2.378866 -2.317325
H    -1.682547 -1.775344 -3.447190
H    -3.205692 -0.882473 -3.232533
H    -2.124294  3.394767 -1.421852
H    -0.543717  2.804574 -1.933745
H    -0.723522  3.578925 -0.340446
H    -3.600541  1.175372  2.545790
H    -2.996847 -0.215652  3.477679
H    -2.133652  1.337881  3.510420
H    -0.756421 -3.765933  1.063466
H     0.743120 -3.304229  0.227558
H     0.488377 -2.838115  1.918590
H    -2.907636  1.037861 -2.045898
H    -0.999664  2.203419  1.465498
H    -1.942482 -1.780234  2.232634
H    -0.207294 -2.141139 -1.521067
    el energy= -873.488028007
          zpe= -873.215518
    th energy= -873.194981
  th enthalpy= -873.194037
  free energy= -873.264532
```

TS-E

```
C    -0.276816  1.073282  1.685384
C     0.077190  2.365380  1.230078
C     0.069321  3.024518  0.000000
C     0.077190  2.365380 -1.230078
C    -0.276816  1.073282 -1.685384
C    -1.374862  0.250517 -1.259008
C    -1.982129 -0.004734  0.000000
C    -1.374862  0.250517  1.259008
Mo    0.236795 -0.712153  0.000000
C     0.202242 -2.207898  1.276084
C     0.202242 -2.207898 -1.276084
C     2.271882 -0.642514  0.000000
O     0.161534 -3.113982  1.992201
O     0.161534 -3.113982 -1.992201
O     3.416960 -0.583013  0.000000
C     0.178579  0.763045  3.107486
C     0.360567  4.507080  0.000000
C     0.178579  0.763045 -3.107486
C    -3.253912 -0.819236  0.000000
H    -0.499579  1.207720  3.852501
H     1.185094  1.151218  3.297815
H     0.211397 -0.316381  3.292766
H    -4.131301 -0.157575  0.000000
```

H   -3.321293 -1.458916 0.887293
H   -3.321293 -1.458916 -0.887293
H   -0.499579 1.207720 -3.852501
H   1.185094 1.151218 -3.297815
H   0.211397 -0.316381 -3.292766
H   0.932363 4.808362 0.887301
H   0.932363 4.808362 -0.887301
H   -0.564465 5.100860 0.000000
H   -1.844024 -0.301068 2.077351
H   -1.844024 -0.301068 -2.077351
H   0.515937 2.975236 -2.028146
H   0.515937 2.975236 2.028146
el energy= -873.482256329
zpe= -873.211284
th energy= -873.190240
th enthalpy= -873.189296
free energy= -873.261034

TS-F
C   0.321311 1.298890 1.622812
C   0.032783 -0.002055 2.106298
C   0.318303 -1.302675 1.619791
C   1.505834 -1.744960 0.850737
C   1.921112 -1.244763 -0.322790
C   1.298188 -0.000869 -0.896375
C   1.921609 1.242399 -0.322388
C   1.507849 1.741255 0.852388
Mo   -0.775017 0.000093 -0.094194
C   -1.791373 1.646888 -0.501590
C   -0.911915 0.003218 -2.054414
C   -1.793437 -1.643902 -0.506524
O   -2.459501 2.574764 -0.653985
O   -0.945425 0.004829 -3.209763
O   -2.463104 -2.570234 -0.661773
C   -0.348329 2.447722 2.353652
C   -0.353832 -2.451701 2.348016
C   3.027045 -1.883693 -1.111035
C   3.024984 1.883770 -1.112219
H   -1.286771 2.142338 2.830825
H   -0.569480 3.279903 1.675539
H   0.314557 2.838421 3.141258
H   3.419560 2.771675 -0.605054
H   2.661278 2.189597 -2.103994
H   3.859369 1.189510 -1.284512
H   3.861749 -1.188688 -1.278812
H   3.420393 -2.772764 -0.604950
H   2.666588 -2.187045 -2.104745
H   0.307653 -2.844961 3.135536
H   -1.292226 -2.145633 2.824872
H   -0.575805 -3.282247 1.668170
H   1.998549 2.636821 1.242715
H   1.443681 -0.000783 -1.982772
H   1.996603 -2.640315 1.241519
H   -0.811515 -0.001754 2.806554
el energy= -873.446752008
zpe= -873.175165
th energy= -873.154805
th enthalpy= -873.153861
free energy= -873.223139

PBEPBE/auto/BS1

II
C   -2.670790 0.155114 0.304182
C   -1.531041 1.086675 0.562385
C   -0.807379 1.758896 -0.455615
C   -0.313272 1.148710 -1.681835
C   -0.060822 -0.194870 -2.084560
C   -0.292375 -1.440765 -1.359817
C   -1.142574 -1.748307 -0.283187
C   -2.469548 -1.113629 -0.084412
Mo   0.622541 0.037469 0.206101
C   0.426060 -0.615133 2.072606
C   2.312381 -0.999620 -0.018182
C   1.881605 1.451144 0.800430
O   0.219026 -1.035634 3.143915
O   3.329703 -1.567325 -0.135607
O   2.674983 2.241149 1.145865
C   -4.045648 0.755437 0.481725
C   -0.460978 3.228536 -0.272264
C   0.705874 -0.369857 -3.388376
C   -1.006485 -3.123212 0.350297
H   -4.196900 1.615032 -0.200530
H   -4.177157 1.146661 1.510010
H   -4.843260 0.016845 0.290150
H   -1.811842 -3.795636 -0.009378
H   -1.106422 -3.063464 1.448969
H   -0.033243 -3.589462 0.119117
H   0.023671 -0.733913 -4.180989
H   1.158875 0.573423 -3.735758
H   1.510679 -1.118440 -3.281475
H   -1.243493 3.855002 -0.745027
H   -0.430449 3.491429 0.798692
H   0.510875 3.498588 -0.720376
H   -3.330899 -1.773485 -0.279353
H   0.385252 -2.244999 -1.682301
H   0.151572 1.879417 -2.358200
H   -1.653725 1.689077 1.476546
el energy= -873.518012298
zpe= -873.254164
th energy= -873.232606
th enthalpy= -873.231662
free energy= -873.303858

TS-A
C   -2.691918 -0.080176 0.507711
C   -1.526815 0.832775 0.757230
C   -1.022362 1.655612 -0.320066
C   -0.587471 1.086410 -1.556361
C   -0.272596 -0.264863 -2.006918
C   -0.353623 -1.511218 -1.303779
C   -1.036533 -1.818449 -0.079978
C   -2.431572 -1.334887 0.124187
Mo   0.613537 0.023697 0.192905
C   1.072193 0.995773 1.861904
C   1.747839 -1.404283 0.991950
C   2.287026 0.757794 -0.619673
O   1.315003 1.602767 2.833657
O   2.425143 -2.235512 1.462877
O   3.234067 1.207167 -1.137744
C   -4.070432 0.515866 0.653621
C   -0.852926 3.151349 -0.126678

```
C     0.403519 -0.314241 -3.371384
C    -0.767039 -3.185115  0.528888
H    -4.215057  1.364112 -0.044492
H    -4.224207  0.920685  1.673378
H    -4.861342 -0.228553  0.456910
H    -1.526287 -3.915307  0.177346
H    -0.843704 -3.146095  1.629382
H     0.230902 -3.576094  0.268342
H    -0.362144 -0.263928 -4.169715
H     1.098822  0.529672 -3.521888
H     0.966512 -1.252142 -3.510309
H    -0.552976  3.373834  0.912285
H    -0.095449  3.584668 -0.802713
H    -1.815760  3.669792 -0.306526
H    -3.235179 -2.055542 -0.103627
H     0.284475 -2.299320 -1.731315
H    -0.203811  1.838200 -2.260879
H    -1.590779  1.371276  1.717553
    el energy= -873.498902969
          zpe= -873.235697
    th energy= -873.214940
  th enthalpy= -873.213996
  free energy= -873.283689

TS-B
C     0.054578  2.404544  1.253551
C    -0.012475  3.035259  0.000000
C     0.054578  2.404544 -1.253551
C    -0.241947  1.049032 -1.614592
C    -1.384714  0.214095 -1.286558
C    -1.963454 -0.015327  0.000000
C    -1.384714  0.214095  1.286558
C    -0.241947  1.049032  1.614592
Mo    0.267728 -0.761090  0.000000
C     2.277479 -0.655614  0.000000
C     0.283271 -2.251293  1.288161
C     0.283271 -2.251293 -1.288161
O     3.440135 -0.549027  0.000000
O     0.271215 -3.162902  2.028135
O     0.271215 -3.162902 -2.028135
C     0.591538  3.243781  2.396636
C     0.591538  3.243781 -2.396636
C    -2.081347 -0.440586 -2.468188
C    -2.081347 -0.440586  2.468188
H     0.958403  4.229526  2.066665
H     1.421201  2.713557  2.901933
H    -0.190252  3.399857  3.165958
H    -2.773035  0.281215  2.945788
H    -1.351153 -0.753518  3.234258
H    -2.662527 -1.328310  2.164275
H    -2.662527 -1.328310 -2.164275
H    -2.773035  0.281215 -2.945788
H    -1.351153 -0.753518 -3.234258
H    -0.190252  3.399857 -3.165958
H     0.958403  4.229526 -2.066665
H     1.421201  2.713557 -2.901933
H     0.129594  0.808028  2.621941
H    -2.846649 -0.666053  0.000000
H     0.129594  0.808028 -2.621941
H     0.211229  4.109002  0.000000
    el energy= -873.501856739
          zpe= -873.238728
    th energy= -873.217603
  th enthalpy= -873.216659
  free energy= -873.288396

TS-C
C     1.752627 -1.721366 -0.228757
C     0.486074 -1.640187 -0.985838
C    -0.144860 -0.715530 -1.854827
C    -0.105792  0.737890 -1.830753
C     0.557136  1.686725 -1.020736
C     1.847967  1.654696 -0.315554
C     2.718344  0.699095  0.143590
C     2.643745 -0.768059  0.186167
Mo   -0.663638 -0.002247  0.304282
C    -1.094621 -1.402515  1.669877
C    -1.107921  1.510073  1.539401
C    -2.533577 -0.021228 -0.109220
O    -1.376512 -2.243751  2.430854
O    -1.378548  2.423453  2.216898
O    -3.680055 -0.034716 -0.379070
C     2.030832 -3.153892  0.198114
C    -1.144747 -1.293120 -2.847230
C     0.052088  3.121838 -1.176305
C     3.988478  1.204480  0.808597
H     2.104201 -3.825048 -0.679948
H     1.199047 -3.537891  0.821693
H     2.962454 -3.242343  0.780655
H     4.881833  0.801131  0.291636
H     4.053661  0.856350  1.858370
H     4.057976  2.304605  0.805702
H     0.737353  3.698299 -1.828800
H    -0.959018  3.160668 -1.613521
H     0.018618  3.638029 -0.200310
H    -0.665283 -1.389985 -3.841224
H    -1.487078 -2.296050 -2.541731
H    -2.032846 -0.648038 -2.965749
H     3.493133 -1.188510  0.742108
H     2.164840  2.677430 -0.067889
H    -0.899988  1.174366 -2.450360
H     0.050167 -2.641942 -1.099078
    el energy= -873.493614740
          zpe= -873.229197
    th energy= -873.208295
  th enthalpy= -873.207351
  free energy= -873.278505

TS-D
C    -1.788207 -0.668946 -1.634433
C    -0.730556 -1.439623 -0.919904
C    -0.680707 -1.748267  0.453129
C    -1.540656 -1.138877  1.486878
C    -1.846320  0.171535  1.718659
C    -1.393283  1.334915  0.916025
C    -1.568567  1.572481 -0.428572
C    -2.159638  0.616853 -1.388867
Mo    0.793490  0.151206 -0.033370
C     2.058319 -0.423205 -1.363759
C     1.854802  1.818657  0.138138
C     2.022367 -0.672348  1.188811
O     2.810679 -0.778852 -2.198906
```

O	2.490622	2.799836	0.221659
O	2.749005	-1.193019	1.957527
C	-2.410909	-1.428646	-2.785328
C	0.014301	-3.028362	0.889868
C	-2.630649	0.563853	2.952479
C	-1.272825	2.946010	-1.000833
H	-2.883419	-2.364388	-2.429153
H	-1.634828	-1.729659	-3.516156
H	-3.171323	-0.830329	-3.315346
H	-2.217322	3.421661	-1.333826
H	-0.619556	2.884357	-1.889865
H	-0.795224	3.607910	-0.259410
H	-3.563701	1.094219	2.679529
H	-2.896062	-0.314481	3.564835
H	-2.047398	1.262197	3.584845
H	-0.734263	-3.838627	1.010725
H	0.753074	-3.355239	0.139081
H	0.537425	-2.913428	1.854966
H	-2.906360	1.061957	-2.064719
H	-1.033662	2.184933	1.516962
H	-1.881478	-1.852187	2.254516
H	-0.199769	-2.149446	-1.571425

el energy= -873.487677852
zpe= -873.224584
th energy= -873.203374
th enthalpy= -873.202430
free energy= -873.274605

TS-E

C	-0.247039	1.103834	1.694400
C	0.103913	2.409998	1.240357
C	0.095652	3.070945	0.000000
C	0.103913	2.409998	-1.240357
C	-0.247039	1.103834	-1.694400
C	-1.363403	0.290912	-1.268689
C	-1.981989	0.044454	0.000000
C	-1.363403	0.290912	1.268689
Mo	0.238860	-0.739858	0.000000
C	0.152694	-2.235828	1.274510
C	0.152694	-2.235828	-1.274510
C	2.270108	-0.734962	0.000000
O	0.075712	-3.152511	2.003312
O	0.075712	-3.152511	-2.003312
O	3.435055	-0.710988	0.000000
C	0.226765	0.781574	3.117363
C	0.372645	4.564952	0.000000
C	0.226765	0.781574	-3.117363
C	-3.279840	-0.744136	0.000000
H	-0.443959	1.230408	3.878674
H	1.245072	1.166361	3.297448
H	0.254457	-0.306918	3.297592
H	-4.148683	-0.057606	0.000000
H	-3.363620	-1.386515	0.894104
H	-3.363620	-1.386515	-0.894104
H	-0.443959	1.230408	-3.878674
H	1.245072	1.166361	-3.297448
H	0.254457	-0.306918	-3.297592
H	0.943999	4.875594	0.894316
H	0.943999	4.875594	-0.894316
H	-0.566267	5.152501	0.000000
H	-1.841641	-0.260446	2.093580
H	-1.841641	-0.260446	-2.093580
H	0.533893	3.028424	-2.048482
H	0.533893	3.028424	2.048482

el energy= -873.486221470
zpe= -873.224261
th energy= -873.202654
th enthalpy= -873.201710
free energy= -873.274699

TS-F

C	-0.329249	1.315456	-1.642078
C	-0.023074	0.002164	-2.115392
C	-0.327316	-1.312421	-1.644243
C	-1.510433	-1.762222	-0.866164
C	-1.941442	-1.261349	0.318144
C	-1.351563	-0.001664	0.900333
C	-1.943086	1.258315	0.320397
C	-1.512823	1.762076	-0.862928
Mo	0.758383	0.000368	0.113088
C	1.803483	1.634128	0.498585
C	0.924462	-0.001780	2.080860
C	1.805702	-1.632630	0.495688
O	2.514220	2.557556	0.630844
O	0.970283	-0.003207	3.252861
O	2.517646	-2.555291	0.626571
C	0.349816	2.468193	-2.376873
C	0.353372	-2.462833	-2.381249
C	-3.023116	-1.940575	1.122046
C	-3.025913	1.934325	1.125472
H	1.288248	2.152694	-2.865126
H	0.586388	3.302117	-1.693259
H	-0.320365	2.870836	-3.163982
H	-3.405302	2.838439	0.617842
H	-2.642590	2.234400	2.121106
H	-3.882958	1.257372	1.311125
H	-3.880883	-1.265134	1.309820
H	-3.401632	-2.843886	0.612332
H	-2.638923	-2.242692	2.116732
H	-0.315573	-2.863900	-3.170218
H	1.292183	-2.145449	-2.867554
H	0.589690	-3.298327	-1.699483
H	-1.982891	2.687173	-1.233505
H	-1.496063	-0.002678	1.994712
H	-1.979137	-2.687291	-1.238556
H	0.827602	0.003318	-2.819483

el energy= -873.452042126
zpe= -873.189973
th energy= -873.168890
th enthalpy= -873.167945
free energy= -873.238889

B3LYP/BS1

```
II
C     2.660489  0.216908 -0.326334
C     1.514236  1.154621 -0.508769
C     0.785395  1.745318  0.537367
C     0.313917  1.074526  1.734773
C     0.092982 -0.278065  2.074297
C     0.352128 -1.486880  1.301891
C     1.209560 -1.752982  0.241630
C     2.501051 -1.061428  0.017470
Mo   -0.647976  0.038678 -0.216029
C    -0.461397 -0.525909 -2.124798
C    -2.312498 -1.061398 -0.004207
C    -1.929300  1.472327 -0.733435
O    -0.264599 -0.881715 -3.206648
O    -3.292461 -1.664297  0.117391
O    -2.714700  2.271785 -1.025737
C     4.022826  0.835567 -0.538179
C     0.412643  3.216096  0.433054
C    -0.639369 -0.532174  3.384082
C     1.117588 -3.114759 -0.424553
H     4.193591  1.665758  0.162643
H     4.108158  1.257571 -1.550395
H     4.826318  0.102090 -0.405315
H     1.950997 -3.756168 -0.098038
H     1.194234 -3.024623 -1.515015
H     0.177386 -3.625692 -0.189419
H     0.064152 -0.908855  4.140560
H    -1.105755  0.376149  3.778754
H    -1.421086 -1.291103  3.261988
H     1.177024  3.823870  0.940380
H     0.380261  3.536192 -0.613661
H    -0.557108  3.441513  0.890051
H     3.380076 -1.698940  0.146414
H    -0.307811 -2.305881  1.589525
H    -0.139442  1.762011  2.448093
H     1.626584  1.804821 -1.379099
    el energy= -874.457243362
          zpe= -874.186011
    th energy= -874.164869
  th enthalpy= -874.163925
  free energy= -874.235147

TS-A
C    -2.676022  0.074974 -0.513618
C    -1.525966 -0.861921 -0.733515
C    -1.030774 -1.656484  0.355945
C    -0.587526 -1.080083  1.575184
C    -0.268271  0.266288  2.009469
C    -0.361738  1.506608  1.312167
C    -1.039942  1.823091  0.103585
C    -2.421774  1.322125 -0.134461
Mo    0.630169 -0.029458 -0.209115
C     1.068506 -1.027920 -1.881173
C     1.735424  1.422443 -1.029892
C     2.323412 -0.746537  0.619137
O     1.283695 -1.640371 -2.839387
O     2.373540  2.264644 -1.502288
O     3.258348 -1.177487  1.142036
C    -4.059879 -0.500342 -0.691042
C    -0.877767 -3.157509  0.189505
C     0.399855  0.326349  3.377330
C    -0.787757  3.202798 -0.481343
H    -4.234836 -1.334510  0.004465
H    -4.191856 -0.904794 -1.705292
H    -4.836907  0.253914 -0.521178
H    -1.546593  3.913390 -0.114059
H    -0.867571  3.185279 -1.574293
H     0.199462  3.596446 -0.218376
H    -0.364987  0.291307  4.166531
H     1.082889 -0.514676  3.540810
H     0.965098  1.254748  3.508034
H    -0.600007 -3.403942 -0.841228
H    -0.120354 -3.581653  0.858348
H    -1.836281 -3.657100  0.395362
H    -3.227347  2.035071  0.067336
H     0.272184  2.287445  1.735175
H    -0.219158 -1.820770  2.284867
H    -1.604168 -1.420491 -1.670230
    el energy= -874.434871656
          zpe= -874.164517
    th energy= -874.144120
  th enthalpy= -874.143176
  free energy= -874.212041

TS-B
C     0.060556  2.389042  1.251635
C     0.013399  3.012758  0.000000
C     0.060556  2.389042 -1.251635
C    -0.269789  1.053230 -1.627741
C    -1.399609  0.221532 -1.281100
C    -1.969212 -0.008103  0.000000
C    -1.399609  0.221532  1.281100
C    -0.269789  1.053230  1.627741
Mo    0.270540 -0.767288  0.000000
C     2.296797 -0.649232  0.000000
C     0.291673 -2.254448  1.312826
C     0.291673 -2.254448 -1.312826
O     3.444019 -0.533458  0.000000
O     0.287670 -3.142963  2.058011
O     0.287670 -3.142963 -2.058011
C     0.609353  3.227285  2.390801
C     0.609353  3.227285 -2.390801
C    -2.111889 -0.428334 -2.456312
C    -2.111889 -0.428334  2.456312
H     0.999325  4.192403  2.053352
H     1.417095  2.687336  2.903134
H    -0.171088  3.410305  3.143668
H    -2.798643  0.294601  2.921095
H    -1.398320 -0.742733  3.225682
H    -2.692749 -1.305324  2.149972
H    -2.692749 -1.305324 -2.149972
H    -2.798643  0.294601 -2.921095
H    -1.398320 -0.742733 -3.225682
H    -0.171088  3.410305 -3.143668
H     0.999325  4.192403 -2.053352
H     1.417095  2.687336 -2.903134
H     0.074449  0.825071  2.637213
H    -2.850768 -0.645449  0.000000
H     0.074449  0.825071 -2.637213
H     0.263651  4.071090  0.000000
```

```
    el energy= -874.440306836
          zpe= -874.170197
    th energy= -874.149419
  th enthalpy= -874.148475
  free energy= -874.219432

TS-C
C    -1.766297 -1.708290  0.212161
C    -0.506545 -1.638341  0.981476
C     0.111208 -0.724122  1.853154
C     0.078012  0.724346  1.838790
C    -0.563669  1.676522  1.035942
C    -1.847068  1.655028  0.313686
C    -2.705291  0.713708 -0.162074
C    -2.637272 -0.754835 -0.208953
Mo    0.688780 -0.003276 -0.304248
C     1.108138 -1.402096 -1.697461
C     1.128170  1.516165 -1.558457
C     2.564156 -0.031098  0.143935
O     1.360318 -2.223372 -2.468262
O     1.377172  2.412790 -2.241598
O     3.691729 -0.049630  0.430914
C    -2.064478 -3.142220 -0.196408
C     1.085067 -1.308487  2.867259
C    -0.068499  3.110598  1.222146
C    -3.967101  1.223449 -0.839308
H    -2.163981 -3.790298  0.686387
H    -1.239715 -3.551097 -0.798139
H    -2.984017 -3.220265 -0.784567
H    -4.860512  0.823142 -0.337604
H    -4.018111  0.885033 -1.884542
H    -4.032570  2.315825 -0.830446
H    -0.756793  3.667173  1.875707
H     0.931178  3.145393  1.665879
H    -0.030014  3.642409  0.264091
H     0.586202 -1.399827  3.843406
H     1.423193 -2.307252  2.572616
H     1.968820 -0.675334  3.004267
H    -3.479771 -1.162095 -0.766900
H    -2.159668  2.673633  0.085566
H     0.853866  1.148253  2.473895
H    -0.091805 -2.637379  1.105018
    el energy= -874.435363396
          zpe= -874.163648
    th energy= -874.143171
  th enthalpy= -874.142226
  free energy= -874.212335

TS-D
C    -1.794308 -0.614418 -1.651709
C    -0.764862 -1.412668 -0.927769
C    -0.744623 -1.732353  0.426940
C    -1.631576 -1.129618  1.444061
C    -1.929072  0.167969  1.679544
C    -1.425954  1.336762  0.912603
C    -1.569615  1.598690 -0.416867
C    -2.153093  0.662193 -1.400438
Mo    0.849947  0.147340 -0.009496
C     2.074508 -0.416053 -1.396396
C     1.955438  1.788646  0.219143
C     2.057492 -0.753315  1.197577
O     2.786228 -0.757833 -2.253148
O     2.601858  2.742279  0.341609
O     2.757655 -1.306500  1.946833
C    -2.414644 -1.358994 -2.814274
C    -0.079226 -3.024571  0.871243
C    -2.779969  0.556930  2.869275
C    -1.245815  2.974141 -0.966452
H    -2.899150 -2.284214 -2.471920
H    -1.642048 -1.657750 -3.537401
H    -3.158724 -0.750461 -3.339514
H    -2.171670  3.460373 -1.309450
H    -0.579085  2.915729 -1.835565
H    -0.780841  3.616702 -0.211838
H    -3.679752  1.101654  2.549245
H    -3.094232 -0.319770  3.445811
H    -2.228138  1.229731  3.542178
H    -0.844236 -3.808463  0.991665
H     0.648014 -3.372977  0.130955
H     0.436478 -2.918852  1.831873
H    -2.884781  1.118808 -2.070374
H    -1.060022  2.156760  1.534894
H    -2.023479 -1.852351  2.163839
H    -0.230103 -2.106835 -1.576821
    el energy= -874.432515279
          zpe= -874.161998
    th energy= -874.141262
  th enthalpy= -874.140318
  free energy= -874.211174

TS-E
C    -0.282003  1.104591  1.705818
C     0.102266  2.388332  1.238344
C     0.117840  3.040424  0.000000
C     0.102266  2.388332 -1.238344
C    -0.282003  1.104591 -1.705818
C    -1.379013  0.286515 -1.263872
C    -1.986409  0.036453  0.000000
C    -1.379013  0.286515  1.263872
Mo    0.244294 -0.745124  0.000000
C     0.177970 -2.240002  1.301282
C     0.177970 -2.240002 -1.301282
C     2.295411 -0.712607  0.000000
O     0.120721 -3.133923  2.036178
O     0.120721 -3.133923 -2.036178
O     3.444938 -0.668265  0.000000
C     0.147352  0.806021  3.147851
C     0.438432  4.526516  0.000000
C     0.147352  0.806021 -3.147851
C    -3.286860 -0.748603  0.000000
H    -0.542788  1.263077  3.874930
H     1.151244  1.193493  3.353654
H     0.170254 -0.270655  3.348060
H    -4.147902 -0.064690  0.000000
H    -3.372520 -1.386330  0.887513
H    -3.372520 -1.386330 -0.887513
H    -0.542788  1.263077 -3.874930
H     1.151244  1.193493 -3.353654
H     0.170254 -0.270655 -3.348060
H     1.015995  4.817256  0.887320
H     1.015995  4.817256 -0.887320
H    -0.475344  5.138719  0.000000
```

H	-1.860648	-0.259249	2.077671
H	-1.860648	-0.259249	-2.077671
H	0.539727	2.998137	-2.035847
H	0.539727	2.998137	2.035847

el energy= -874.423497037
zpe= -874.154881
th energy= -874.133497
th enthalpy= -874.132553
free energy= -874.205149

TS-F

C	0.366678	1.305038	1.628255
C	0.075424	-0.000933	2.103917
C	0.366207	-1.306649	1.627338
C	1.540609	-1.752238	0.832182
C	1.940361	-1.252220	-0.349978
C	1.316211	0.000144	-0.918226
C	1.940807	1.251871	-0.349067
C	1.541287	1.750964	0.833551
Mo	-0.794633	0.000160	-0.112442
C	-1.827924	1.676363	-0.456878
C	-0.970179	0.001097	-2.091859
C	-1.828740	-1.675362	-0.458201
O	-2.514464	2.601844	-0.557658
O	-1.017377	0.001667	-3.248960
O	-2.515753	-2.600425	-0.559590
C	-0.282670	2.456055	2.390891
C	-0.283385	-2.458064	2.389144
C	3.031686	-1.906248	-1.161610
C	3.032187	1.906299	-1.160291
H	-1.208648	2.149820	2.891307
H	-0.518645	3.296755	1.728431
H	0.403227	2.837004	3.164277
H	3.425107	2.799337	-0.660120
H	2.652925	2.207408	-2.148236
H	3.871944	1.220487	-1.343208
H	3.871408	-1.220313	-1.344223
H	3.424665	-2.799527	-0.661914
H	2.652377	-2.206863	-2.149689
H	0.402844	-2.840321	3.161590
H	-1.208771	-2.151741	2.890597
H	-0.520411	-3.297884	1.725936
H	2.031484	2.651317	1.212200
H	1.442315	0.000481	-2.005500
H	2.030566	-2.653049	1.210059
H	-0.748414	-0.001018	2.825723

el energy= -874.386357438
zpe= -874.117063
th energy= -874.096420
th enthalpy= -874.095475
free energy= -874.165576

Molecular Coordinates for $(TMCOT)W(CO)_3$ (Å)

PBE0/BS1

```
II
C     0.903707  1.734886  0.435617
C     0.380669  1.171798  1.657244
C     0.096704 -0.152948  2.089802
C     0.325141 -1.410981  1.413434
C     1.152577 -1.716497  0.320360
C     2.510225 -1.140462  0.180581
C     2.745108  0.101171 -0.231361
C     1.608861  1.007142 -0.554720
W    -0.499193  0.027035 -0.171608
C    -1.721209  1.452238 -0.823359
C    -0.270867 -0.696502 -2.009014
C    -2.217779 -0.951327  0.080384
O    -2.475456  2.243008 -1.199543
O    -0.042156 -1.147591 -3.044847
O    -3.233998 -1.484039  0.217315
C     0.601792  3.198463  0.210318
C    -0.715417 -0.270191  3.362400
C     1.000708 -3.093189 -0.289188
C     4.117622  0.695193 -0.359331
H     1.406685  3.809236  0.644341
H    -0.346754  3.509641  0.662106
H     0.559027  3.422903 -0.860809
H     4.235823  1.560525  0.308364
H     4.291441  1.061329 -1.381364
H     4.899753 -0.033927 -0.119924
H     1.725849 -3.792658  0.154246
H    -0.007342 -3.495570 -0.138025
H     1.196578 -3.067518 -1.367679
H    -0.062760 -0.570873  4.193641
H    -1.196420  0.674879  3.632879
H    -1.496323 -1.032729  3.265500
H     1.753414  1.579791 -1.474904
H     3.333953 -1.806719  0.452181
H    -0.345228 -2.200752  1.757691
H    -0.101546  1.918103  2.288365
    el energy= -873.819967054
          zpe= -873.546548
    th energy= -873.525835
  th enthalpy= -873.524891
  free energy= -873.595265
```

```
TS-A
C    -2.751085  0.130965 -0.460722
C    -1.593652 -0.768998 -0.745046
C    -1.109115 -1.632576  0.302839
C    -0.663756 -1.100136  1.540911
C    -0.328968  0.227700  2.024956
C    -0.396112  1.482941  1.360081
C    -1.055907  1.791187  0.126056
C    -2.467434  1.357982 -0.045380
W     0.494411 -0.013695 -0.158502
C     0.913658 -0.902504 -1.883053
C     1.683694  1.422623 -0.865832
C     2.125478 -0.882295  0.610415
O     1.122977 -1.451385 -2.877855
O     2.382462  2.241827 -1.282138
O     3.036325 -1.393994  1.097722
C    -4.127688 -0.444329 -0.611761
C    -0.979439 -3.118894  0.082151
C     0.365388  0.222273  3.371657
C    -0.785878  3.169205 -0.441719
H    -4.275606 -1.300141  0.062609
H    -4.288443 -0.817568 -1.633341
H    -4.902924  0.298919 -0.394445
H    -1.507775  3.895742 -0.034773
H    -0.903825  3.170476 -1.531090
H     0.221626  3.530731 -0.209564
H    -0.382013  0.138821  4.172654
H     1.059979 -0.618892  3.474191
H     0.925807  1.147829  3.536214
H    -1.942654 -3.612983  0.276257
H    -0.710646 -3.327978 -0.959239
H    -0.221670 -3.575713  0.728740
H    -3.243731  2.080326  0.224558
H     0.229176  2.254238  1.814385
H    -0.275290 -1.866463  2.213355
H    -1.677600 -1.286370 -1.706527
    el energy= -873.802256715
          zpe= -873.529467
    th energy= -873.509501
  th enthalpy= -873.508557
  free energy= -873.576771
```

```
TS-B
C     0.039764  2.496184  1.236992
C    -0.047133  3.133842  0.000000
C     0.039764  2.496184 -1.236992
C    -0.229136  1.136880 -1.571489
C    -1.395409  0.323638 -1.272639
C    -1.986525  0.115114  0.000000
C    -1.395409  0.323638  1.272639
C    -0.229136  1.136880  1.571489
W     0.208513 -0.631433  0.000000
C     2.213073 -0.540618  0.000000
C     0.212427 -2.119327  1.288584
C     0.212427 -2.119327 -1.288584
O     3.360247 -0.437454  0.000000
O     0.190409 -3.012788  2.027149
O     0.190409 -3.012788 -2.027149
C     0.575138  3.319889  2.378364
C     0.575138  3.319889 -2.378364
C    -2.091736 -0.297777 -2.459879
C    -2.091736 -0.297777  2.459879
H     0.906884  4.314227  2.064620
H     1.422048  2.802132  2.848454
H    -0.191383  3.434286  3.157881
H    -2.756513  0.438783  2.933685
H    -1.365791 -0.621644  3.213757
H    -2.690572 -1.168350  2.169568
H    -2.690572 -1.168350 -2.169568
H    -2.756513  0.438783 -2.933685
H    -1.365791 -0.621644 -3.213757
H    -0.191383  3.434286 -3.157881
```

```
H     0.906884  4.314227 -2.064620
H     1.422048  2.802132 -2.848454
H     0.144903  0.893319  2.568443
H    -2.870645 -0.520348  0.000000
H     0.144903  0.893319 -2.568443
H     0.163948  4.201248  0.000000
    el energy= -873.797612222
          zpe= -873.525220
    th energy= -873.504828
  th enthalpy= -873.503884
  free energy= -873.574237
```

TS-C

```
C    -1.871134 -1.676311  0.227647
C    -0.595221 -1.620821  0.965064
C     0.023627 -0.736590  1.880538
C     0.012484  0.700994  1.878657
C    -0.583717  1.648932  1.020671
C    -1.899246  1.664398  0.359667
C    -2.792822  0.748816 -0.088001
C    -2.742870 -0.714457 -0.160107
W     0.562168 -0.007421 -0.225332
C     0.918404 -1.360020 -1.659461
C     1.007680  1.494735 -1.465503
C     2.433108 -0.065912  0.173516
O     1.132316 -2.161042 -2.459698
O     1.265553  2.391591 -2.142778
O     3.563487 -0.105119  0.446085
C    -2.162816 -3.090499 -0.217977
C     0.997649 -1.356570  2.859155
C    -0.048760  3.064040  1.184349
C    -4.062710  1.282530 -0.708640
H    -2.237170 -3.766530  0.645470
H    -1.345371 -3.468395 -0.849019
H    -3.092272 -3.159035 -0.791268
H    -4.942340  0.886496 -0.180882
H    -4.154267  0.960012 -1.755769
H    -4.112485  2.375358 -0.681716
H    -0.694325  3.637076  1.865719
H     0.969955  3.071346  1.584191
H    -0.033725  3.592290  0.223884
H     0.490457 -1.536034  3.817657
H     1.373080 -2.318277  2.494440
H     1.858847 -0.706748  3.049632
H    -3.595586 -1.106811 -0.714568
H    -2.193325  2.692633  0.146136
H     0.794770  1.116431  2.513342
H    -0.179432 -2.623674  1.060963
    el energy= -873.784791038
          zpe= -873.511046
    th energy= -873.490847
  th enthalpy= -873.489903
  free energy= -873.559773
```

TS-D

```
C    -1.772949 -0.311127 -1.777747
C    -1.334114 -1.418840 -0.908376
C    -1.589429 -1.601074  0.423071
C    -2.370087 -0.666738  1.253780
C    -2.077822  0.622390  1.489369
C    -0.932205  1.356258  0.883785
C    -0.820200  1.682393 -0.484837
C    -1.567561  1.013052 -1.554248
W     0.653277 -0.102266  0.091134
C     1.832187 -1.698463  0.011983
C     1.782314  0.729229 -1.222263
C     1.906973  0.649244  1.341752
O     2.529431 -2.620923 -0.015401
O     2.427370  1.246097 -2.041149
O     2.635110  1.107121  2.127076
C    -2.397016 -0.774764 -3.066483
C    -1.233630 -2.907025  1.087921
C    -2.887617  1.437322  2.457578
C    -0.209840  3.007684 -0.877554
H    -3.319462 -1.340962 -2.876439
H    -1.718028 -1.449219 -3.607602
H    -2.637213  0.066916 -3.724243
H    -1.004761  3.758901 -1.006827
H     0.349391  2.947155 -1.817870
H     0.473279  3.367301 -0.101108
H    -3.297152  2.329305  1.963885
H    -3.715626  0.862669  2.886470
H    -2.253706  1.797658  3.280094
H    -2.156327 -3.447727  1.346727
H    -0.628178 -3.546875  0.437787
H    -0.689638 -2.745973  2.026543
H    -1.864004  1.677736 -2.369823
H    -0.499713  2.098093  1.558833
H    -3.206131 -1.121978  1.789558
H    -0.884583 -2.260901 -1.440468
    el energy= -873.775013738
          zpe= -873.502858
    th energy= -873.482294
  th enthalpy= -873.481350
  free energy= -873.552459
```

TS-E

```
C    -0.262420  1.176704  1.656849
C     0.076754  2.482802  1.224542
C     0.063520  3.149365  0.000000
C     0.076754  2.482802 -1.224542
C    -0.262420  1.176704 -1.656849
C    -1.397949  0.379720 -1.256070
C    -2.019537  0.144252  0.000000
C    -1.397949  0.379720  1.256070
W     0.183503 -0.605599  0.000000
C     0.132073 -2.100014  1.275055
C     0.132073 -2.100014 -1.275055
C     2.202406 -0.564394  0.000000
O     0.077095 -3.001206  2.001205
O     0.077095 -3.001206 -2.001205
O     3.350428 -0.506262  0.000000
C     0.207747  0.854759  3.072938
C     0.347993  4.632417  0.000000
C     0.207747  0.854759 -3.072938
C    -3.306674 -0.644702  0.000000
H    -0.435575  1.335651  3.826233
H     1.234306  1.198749  3.239874
H     0.195123 -0.223563  3.265256
H    -4.168776  0.036534  0.000000
H    -3.385986 -1.282562  0.887397
H    -3.385986 -1.282562 -0.887397
```

```
H    -0.435575  1.335651 -3.826233
H     1.234306  1.198749 -3.239874
H     0.195123 -0.223563 -3.265256
H     0.918149  4.935783  0.887482
H     0.918149  4.935783 -0.887482
H    -0.580331  5.220662  0.000000
H    -1.876058 -0.150243  2.082981
H    -1.876058 -0.150243 -2.082981
H     0.516166  3.084819 -2.028243
H     0.516166  3.084819  2.028243
    el energy= -873.778974693
          zpe= -873.507541
    th energy= -873.486716
  th enthalpy= -873.485772
  free energy= -873.557290

TS-F
C    -0.470570 -1.305851  1.610850
C    -0.198439 -0.000761  2.101198
C    -0.471834  1.304366  1.611734
C    -1.656343  1.749006  0.837455
C    -2.054913  1.243644 -0.339399
C    -1.408162 -0.000373 -0.894509
C    -2.054398 -1.244793 -0.339554
C    -1.655257 -1.750642  0.836872
W     0.649728  0.000005 -0.063298
C     1.708995 -1.610804 -0.454969
C     0.838726  0.001434 -2.029198
C     1.707768  1.612158 -0.452754
O     2.378665 -2.543756 -0.595401
O     0.903186  0.002231 -3.185059
O     2.376536  2.545927 -0.591888
C     0.199121 -2.450685  2.347956
C     0.196873  2.449286  2.349619
C    -3.150998  1.873294 -1.147120
C    -3.151119 -1.873874 -1.146875
H     1.128886 -2.138866  2.838120
H     0.437664 -3.276906  1.668553
H    -0.470122 -2.849832  3.125475
H    -3.558877 -2.761174 -0.649198
H    -2.775682 -2.178126 -2.134624
H    -3.977849 -1.172834 -1.327519
H    -3.977739  1.172517 -1.328717
H    -3.558919  2.760432 -0.649294
H    -2.774807  2.177964 -2.134453
H    -0.473238  2.848074  3.126570
H     1.126245  2.137648  2.840639
H     0.435805  3.275740  1.670645
H    -2.150520 -2.644549  1.224604
H    -1.529304 -0.000380 -1.983808
H    -2.151758  2.642817  1.225189
H     0.646550 -0.000548  2.802849
    el energy= -873.746999809
          zpe= -873.475227
    th energy= -873.454966
  th enthalpy= -873.454021
  free energy= -873.523376

TS-G
C     1.332133 -1.272614 -0.923936
C     1.413934 -0.000004 -1.545468
C     1.332141  1.272607 -0.923935
C     1.825664  1.759848  0.390409
C     1.422983  1.230776  1.555167
C     0.575034  0.000001  1.703745
C     1.422968 -1.230783  1.555168
C     1.825644 -1.759861  0.390410
W    -0.556934  0.000002 -0.250707
C    -1.649738 -1.580389 -0.650533
C    -2.004203  0.000010  1.094250
C    -1.649726  1.580399 -0.650537
O    -2.281205 -2.486346 -0.996356
O    -2.870754  0.000015  1.862907
O    -2.281188  2.486359 -0.996363
H     1.210065 -2.085710 -1.642394
H     1.210072  2.085705 -1.642391
H     1.749765  1.699936  2.487592
H     1.749739 -1.699950  2.487593
C     2.709280 -2.972685  0.328686
C    -0.080935  0.000005  3.080702
C     2.709322  2.972655  0.328680
C     1.200305 -0.000007 -3.051444
H     2.985412 -3.319458  1.330590
H     2.209134 -3.801596 -0.192964
H     3.631371 -2.758194 -0.230250
H     0.662686  0.889658 -3.397190
H     2.185612 -0.000051 -3.538353
H     0.662614 -0.889633 -3.397177
H     2.985457  3.319430  1.330583
H     3.631411  2.758143 -0.230250
H     2.209194  3.801573 -0.192977
H    -0.704475 -0.887371  3.234285
H     0.695859 -0.000004  3.861638
H    -0.704456  0.887394  3.234288
    el energy= -873.742780949
          zpe= -873.470843
    th energy= -873.450690
  th enthalpy= -873.449746
  free energy= -873.518944
```

PBEPBE/auto/BS1

```
II
C     0.916790  1.751205  0.432822
C     0.409202  1.176757  1.669682
C     0.139818 -0.160247  2.105291
C     0.359976 -1.423537  1.410301
C     1.194819 -1.739046  0.311944
C     2.548083 -1.145533  0.145843
C     2.770511  0.111642 -0.265299
C     1.621300  1.020707 -0.571609
W    -0.513545  0.029637 -0.170787
C    -1.752410  1.453794 -0.796623
C    -0.328587 -0.688460 -2.016041
C    -2.222881 -0.964545  0.103813
O    -2.528142  2.255739 -1.159382
O    -0.118988 -1.148977 -3.071661
O    -3.247838 -1.513727  0.257206
C     0.609570  3.224339  0.215827
C    -0.651543 -0.290357  3.400594
C     1.035064 -3.120394 -0.304850
```

```
C     4.145971  0.715326 -0.415757
H     1.420813  3.839686  0.653118
H    -0.344706  3.534732  0.675733
H     0.562382  3.457274 -0.861539
H     4.275794  1.586008  0.257002
H     4.302848  1.091525 -1.446091
H     4.942035 -0.015906 -0.191908
H     1.795794 -3.817443  0.101977
H     0.033704 -3.542604 -0.111721
H     1.184280 -3.082983 -1.398791
H     0.020424 -0.611803  4.219834
H    -1.122298  0.660997  3.698774
H    -1.445658 -1.051866  3.309107
H     1.750455  1.595156 -1.503140
H     3.389557 -1.814038  0.391263
H    -0.311010 -2.221235  1.761567
H    -0.066418  1.925184  2.318139
    el energy= -873.824313113
          zpe= -873.560388
    th energy= -873.538969
  th enthalpy= -873.538025
  free energy= -873.610001
```

TS-A

```
C    -2.781697  0.123523 -0.488088
C    -1.610425 -0.778678 -0.757360
C    -1.127864 -1.641946  0.307251
C    -0.691336 -1.093512  1.554805
C    -0.366796  0.249608  2.031402
C    -0.425146  1.505374  1.340726
C    -1.092745  1.814992  0.099189
C    -2.505205  1.368868 -0.084810
W     0.507766 -0.018525 -0.159121
C     0.964888 -0.930164 -1.865940
C     1.710418  1.409922 -0.863525
C     2.134049 -0.856247  0.646928
O     1.204736 -1.498282 -2.863251
O     2.429414  2.238289 -1.277016
O     3.053132 -1.360147  1.169443
C    -4.161000 -0.466571 -0.640515
C    -0.998100 -3.139107  0.099230
C     0.311318  0.267215  3.396257
C    -0.806476  3.187891 -0.493173
H    -4.309192 -1.323615  0.046102
H    -4.316672 -0.857048 -1.665656
H    -4.949690  0.277534 -0.433759
H    -1.544349  3.927854 -0.117391
H    -0.900242  3.166554 -1.592777
H     0.203203  3.555708 -0.243638
H    -0.454647  0.210658  4.193698
H     0.998600 -0.585957  3.530167
H     0.884487  1.196498  3.550228
H    -1.971374 -3.634571  0.288241
H    -0.717797 -3.359715 -0.945470
H    -0.242626 -3.596514  0.761766
H    -3.293146  2.098839  0.165205
H     0.203185  2.287891  1.792372
H    -0.308126 -1.858476  2.245324
H    -1.677845 -1.300702 -1.727117
    el energy= -873.807982496
          zpe= -873.544639
    th energy= -873.523983
  th enthalpy= -873.523039
  free energy= -873.592805
```

TS-B

```
C     0.055726  2.524995  1.248397
C    -0.020363  3.163010  0.000000
C     0.055726  2.524995 -1.248397
C    -0.223863  1.156544 -1.583810
C    -1.403884  0.347929 -1.283822
C    -1.997822  0.137455  0.000000
C    -1.403884  0.347929  1.283822
C    -0.223863  1.156544  1.583810
W     0.210507 -0.648118  0.000000
C     2.214003 -0.573796  0.000000
C     0.200495 -2.137832  1.292758
C     0.200495 -2.137832 -1.292758
O     3.379460 -0.470683  0.000000
O     0.170943 -3.041667  2.045472
O     0.170943 -3.041667 -2.045472
C     0.587976  3.353886  2.399315
C     0.587976  3.353886 -2.399315
C    -2.110856 -0.270940 -2.478126
C    -2.110856 -0.270940  2.478126
H     0.933317  4.352134  2.083859
H     1.431985  2.829781  2.887330
H    -0.188718  3.480556  3.179143
H    -2.784673  0.472908  2.947401
H    -1.384008 -0.590236  3.244759
H    -2.711802 -1.150603  2.188784
H    -2.711802 -1.150603 -2.188784
H    -2.784673  0.472908 -2.947401
H    -1.384008 -0.590236 -3.244759
H    -0.188718  3.480556 -3.179143
H     0.933317  4.352134 -2.083859
H     1.431985  2.829781 -2.887330
H     0.159571  0.902307  2.583949
H    -2.894486 -0.494994  0.000000
H     0.159571  0.902307 -2.583949
H     0.192848  4.239160  0.000000
    el energy= -873.807253555
          zpe= -873.544100
    th energy= -873.523072
  th enthalpy= -873.522128
  free energy= -873.593888
```

TS-C

```
C    -1.858226 -1.719941  0.233959
C    -0.586915 -1.628936  0.988735
C     0.019288 -0.715936  1.900305
C    -0.018953  0.734287  1.875670
C    -0.657599  1.670117  1.018870
C    -1.964980  1.643783  0.336326
C    -2.846348  0.692484 -0.107593
C    -2.762657 -0.773592 -0.165804
W     0.578974 -0.002242 -0.233598
C     0.995385 -1.381975 -1.620729
C     0.991535  1.502783 -1.483608
C     2.451880 -0.006463  0.189682
O     1.245943 -2.216745 -2.402689
O     1.219580  2.418487 -2.177235
```

```
O     3.594765 -0.013717  0.484314
C    -2.109995 -3.149254 -0.218692
C     0.997923 -1.305458  2.906534
C    -0.149138  3.107114  1.166694
C    -4.130984  1.199556 -0.741986
H    -2.169228 -3.837562  0.647056
H    -1.272378 -3.505529 -0.850828
H    -3.040445 -3.243766 -0.802196
H    -5.012590  0.793317 -0.207478
H    -4.218952  0.856540 -1.791819
H    -4.200910  2.299614 -0.732358
H    -0.826879  3.684408  1.826128
H     0.867027  3.144531  1.592281
H    -0.123986  3.620665  0.189191
H     0.488658 -1.443716  3.880547
H     1.369229 -2.291394  2.579818
H     1.869303 -0.648167  3.071707
H    -3.610328 -1.196197 -0.722771
H    -2.279959  2.668765  0.094970
H     0.759341  1.177907  2.510728
H    -0.145563 -2.629143  1.095960
    el energy= -873.793480660
          zpe= -873.529099
    th energy= -873.508251
  th enthalpy= -873.507307
  free energy= -873.578629

TS-D
C    -2.413558  0.072179 -1.293600
C    -1.451064 -1.016369 -0.913777
C    -1.384739 -1.557721  0.398936
C    -1.587115 -0.784759  1.604535
C    -1.291746  0.554644  1.915445
C    -0.712001  1.576074  1.061973
C    -0.883862  1.812646 -0.331282
C    -2.128501  1.357115 -1.022309
W     0.603452 -0.063599 -0.189145
C     1.598863 -1.516432 -1.074954
C     2.085137  1.052020 -0.799469
C     1.651020 -0.326805  1.436115
O     2.181325 -2.371241 -1.635518
O     2.961076  1.720242 -1.220401
O     2.215576 -0.489372  2.455849
C    -3.681795 -0.381271 -1.973477
C    -1.157323 -3.046910  0.584721
C    -1.309467  0.928625  3.387163
C    -0.294347  3.101339 -0.893773
H    -4.260778 -1.066182 -1.323220
H    -3.455276 -0.947138 -2.898942
H    -4.328354  0.471844 -2.243043
H    -1.046758  3.915995 -0.847291
H    -0.006610  2.983444 -1.952666
H     0.598857  3.423876 -0.333059
H    -1.875673  1.864008  3.551061
H    -1.755458  0.136066  4.010522
H    -0.278397  1.106279  3.750018
H    -2.134441 -3.567054  0.655609
H    -0.619068 -3.471104 -0.279574
H    -0.582761 -3.275253  1.498396
H    -2.836899  2.155183 -1.299446
H    -0.109729  2.314820  1.611825
H    -1.795634 -1.404278  2.489408
H    -1.269295 -1.757440 -1.709837
    el energy= -873.784889171
          zpe= -873.522580
    th energy= -873.501285
  th enthalpy= -873.500341
  free energy= -873.572972

TS-E
C    -0.238419  1.207195  1.668463
C     0.102644  2.525327  1.235505
C     0.093684  3.192891  0.000000
C     0.102644  2.525327 -1.235505
C    -0.238419  1.207195 -1.668463
C    -1.391265  0.420214 -1.266035
C    -2.023291  0.192519  0.000000
C    -1.391265  0.420214  1.266035
W     0.181347 -0.627915  0.000000
C     0.094116 -2.121862  1.278927
C     0.094116 -2.121862 -1.278927
C     2.197217 -0.637246  0.000000
O     0.019750 -3.032546  2.019523
O     0.019750 -3.032546 -2.019523
O     3.364717 -0.601548  0.000000
C     0.244494  0.875269  3.087752
C     0.369029  4.686507  0.000000
C     0.244494  0.875269 -3.087752
C    -3.334719 -0.573259  0.000000
H    -0.386604  1.371293  3.853545
H     1.285723  1.205654  3.243775
H     0.215248 -0.210549  3.281481
H    -4.189862  0.130219  0.000000
H    -3.428944 -1.213897  0.894217
H    -3.428944 -1.213897 -0.894217
H    -0.386604  1.371293 -3.853545
H     1.285723  1.205654 -3.243775
H     0.215248 -0.210549 -3.281481
H     0.940021  4.997238  0.894357
H     0.940021  4.997238 -0.894357
H    -0.570791  5.272258  0.000000
H    -1.879535 -0.108331  2.099577
H    -1.879535 -0.108331 -2.099577
H     0.532522  3.135691 -2.049940
H     0.532522  3.135691  2.049940
    el energy= -873.790516382
          zpe= -873.528248
    th energy= -873.506825
  th enthalpy= -873.505881
  free energy= -873.578681

TS-F
C    -0.469538 -1.318781  1.634770
C    -0.175870 -0.000351  2.112025
C    -0.469729  1.318288  1.635790
C    -1.654063  1.768915  0.858092
C    -2.072126  1.260019 -0.326809
C    -1.455141 -0.000009 -0.892217
C    -2.072946 -1.259703 -0.326731
C    -1.654767 -1.768880  0.857940
W     0.638168 -0.000262 -0.082505
C     1.717417 -1.609670 -0.447960
```

```
C     0.842856  0.000881 -2.059296
C     1.718095  1.609242 -0.446292
O     2.423956 -2.542495 -0.561435
O     0.911601  0.001592 -3.231415
O     2.424877  2.541937 -0.558811
C     0.210692 -2.466202  2.377157
C     0.210351  2.465341  2.378837
C    -3.152603  1.922851 -1.144512
C    -3.155083 -1.920870 -1.143591
H     0.457310 -3.298796  1.695510
H    -0.462063 -2.870729  3.160735
H     1.144578 -2.144909  2.870689
H    -3.548369 -2.823075 -0.643259
H    -2.765736 -2.221175 -2.136721
H    -4.003151 -1.234308 -1.334549
H    -4.000067  1.236587 -1.339026
H    -3.547131  2.823868 -0.643032
H    -2.761001  2.225519 -2.136053
H    -0.462995  2.870104  3.161776
H     1.143579  2.143570  2.873266
H     0.457981  3.297896  1.697521
H    -2.131141 -2.689373  1.231531
H    -1.582587  0.000050 -1.988916
H    -2.129793  2.689962  1.231107
H     0.677428 -0.000522  2.814869
    el energy= -873.759083233
          zpe= -873.496980
    th energy= -873.475949
  th enthalpy= -873.475005
  free energy= -873.546141

TS-G
C     1.354734 -1.284905 -0.943913
C     1.423059 -0.000056 -1.567534
C     1.354736  1.284823 -0.943950
C     1.810189  1.797300  0.377060
C     1.426193  1.258780  1.562419
C     0.633291  0.000030  1.756296
C     1.426130 -1.258752  1.562478
C     1.810130 -1.797332  0.377141
W    -0.544503  0.000003 -0.229008
C    -1.645146 -1.562705 -0.685857
C    -2.007278  0.000016  1.115093
C    -1.645081  1.562744 -0.685848
O    -2.293005 -2.457283 -1.089849
O    -2.884439  0.000027  1.897561
O    -2.292906  2.457358 -1.089829
H     1.230640 -2.098303 -1.673424
H     1.230637  2.098186 -1.673499
H     1.685089  1.798731  2.487381
H     1.684988 -1.798698  2.487449
C     2.602714 -3.082250  0.322330
C    -0.023712  0.000083  3.141956
C     2.602812  3.082169  0.322176
C     1.247735 -0.000077 -3.087076
H     2.841734 -3.457227  1.332356
H     2.042658 -3.873555 -0.214211
H     3.551483 -2.938106 -0.230847
H     0.712517  0.895213 -3.446917
H     2.250747 -0.000076 -3.556920
H     0.712525 -0.895379 -3.446894
H     2.841865  3.457196  1.332177
H     3.551567  2.937979 -0.231010
H     2.042778  3.873473 -0.214391
H    -0.652714 -0.893052  3.293839
H     0.759156  0.000102  3.928064
H    -0.652690  0.893247  3.293777
    el energy= -873.755722199
          zpe= -873.493412
    th energy= -873.472448
  th enthalpy= -873.471504
  free energy= -873.542635
```

B3LYP/BS1

```
II
C     0.905245  1.741675  0.494467
C     0.412839  1.125812  1.708557
C     0.162346 -0.217434  2.098423
C     0.406587 -1.453837  1.374612
C     1.242419 -1.743185  0.289739
C     2.570069 -1.107988  0.094766
C     2.762085  0.153667 -0.286765
C     1.609355  1.071804 -0.535230
W    -0.529349  0.030275 -0.178119
C    -1.781820  1.470070 -0.748881
C    -0.349813 -0.628155 -2.055162
C    -2.220641 -1.008456  0.090055
O    -2.547946  2.279688 -1.070713
O    -0.151187 -1.042016 -3.117387
O    -3.216568 -1.580596  0.248174
C     0.584763  3.219044  0.334882
C    -0.602894 -0.400960  3.401800
C     1.118567 -3.120513 -0.341503
C     4.131515  0.763556 -0.465423
H     1.384562  3.818297  0.795182
H    -0.364646  3.500838  0.803422
H     0.536939  3.493346 -0.724216
H     4.279267  1.612374  0.218234
H     4.255315  1.157446 -1.484960
H     4.927948  0.032895 -0.282940
H     1.913371 -3.785151  0.031592
H     0.150519 -3.585639 -0.124461
H     1.232999 -3.065273 -1.430824
H     0.084170 -0.728026  4.195362
H    -1.083539  0.525647  3.731348
H    -1.378067 -1.169504  3.303680
H     1.732628  1.683478 -1.432030
H     3.424901 -1.761231  0.290440
H    -0.250458 -2.260466  1.701463
H    -0.052168  1.843408  2.382928
    el energy= -874.751367540
          zpe= -874.480216
    th energy= -874.459127
  th enthalpy= -874.458183
  free energy= -874.529633

TS-A
C    -2.762565  0.121687 -0.501754
C    -1.603407 -0.801060 -0.741425
C    -1.136820 -1.643856  0.335122
C    -0.696301 -1.095393  1.568583
C    -0.367807  0.241967  2.037827
```

```
C    -0.438534  1.493852  1.361399
C    -1.089296  1.813597  0.126524
C    -2.491618  1.357066 -0.096046
W     0.518233 -0.020595 -0.167939
C     0.961213 -0.952856 -1.874799
C     1.690075  1.430195 -0.895397
C     2.164018 -0.843018  0.647252
O     1.180618 -1.525399 -2.858641
O     2.370818  2.267522 -1.317609
O     3.076924 -1.328939  1.165372
C    -4.147223 -0.445304 -0.689300
C    -1.025545 -3.144527  0.144536
C     0.297851  0.259799  3.408269
C    -0.818812  3.203376 -0.432032
H    -4.326971 -1.291405 -0.009551
H    -4.281016 -0.830091 -1.711035
H    -4.921356  0.308736 -0.505646
H    -1.548782  3.923738 -0.027064
H    -0.926852  3.213320 -1.522600
H     0.184259  3.568099 -0.187648
H    -0.468586  0.190918  4.193399
H     0.987808 -0.580628  3.543171
H     0.855618  1.187453  3.571220
H    -1.999341 -3.619468  0.336875
H    -0.748471 -3.381576 -0.888499
H    -0.284734 -3.600886  0.811029
H    -3.281075  2.079802  0.131356
H     0.179840  2.269053  1.816730
H    -0.328691 -1.852560  2.261099
H    -1.679145 -1.339088 -1.690897
    el energy= -874.732922814
          zpe= -874.462400
    th energy= -874.442146
  th enthalpy= -874.441202
  free energy= -874.510043
```

TS-B

```
C     0.060956  2.512547  1.245525
C     0.002167  3.144930  0.000000
C     0.060956  2.512547 -1.245525
C    -0.246846  1.160652 -1.592827
C    -1.415513  0.355224 -1.277315
C    -2.002468  0.147571  0.000000
C    -1.415513  0.355224  1.277315
C    -0.246846  1.160652  1.592827
W     0.212777 -0.652460  0.000000
C     2.227856 -0.571357  0.000000
C     0.205344 -2.138209  1.309467
C     0.205344 -2.138209 -1.309467
O     3.378877 -0.466184  0.000000
O     0.178795 -3.022474  2.063987
O     0.178795 -3.022474 -2.063987
C     0.606701  3.339003  2.392489
C     0.606701  3.339003 -2.392489
C    -2.136328 -0.256567 -2.466605
C    -2.136328 -0.256567  2.466605
H     0.971176  4.319194  2.070061
H     1.432715  2.805712  2.882516
H    -0.166666  3.488616  3.159877
H    -2.789855  0.494184  2.935123
H    -1.424382 -0.593772  3.227790
H    -2.752520 -1.114070  2.173740
H    -2.752520 -1.114070 -2.173740
H    -2.789855  0.494184 -2.935123
H    -1.424382 -0.593772 -3.227790
H    -0.166666  3.488616 -3.159877
H     0.971176  4.319194 -2.070061
H     1.432715  2.805712 -2.882516
H     0.111876  0.918159  2.594357
H    -2.898141 -0.470089  0.000000
H     0.111876  0.918159 -2.594357
H     0.239135  4.206453  0.000000
    el energy= -874.734763768
          zpe= -874.464468
    th energy= -874.443807
  th enthalpy= -874.442863
  free energy= -874.513851
```

TS-C

```
C    -1.888327 -1.701927  0.189557
C    -0.624036 -1.623967  0.957358
C    -0.044841 -0.726169  1.887899
C    -0.072903  0.716047  1.874472
C    -0.672123  1.656119  1.009506
C    -1.975601  1.646616  0.315114
C    -2.850810  0.713051 -0.141267
C    -2.774365 -0.753740 -0.209157
W     0.603218 -0.004419 -0.227325
C     1.040191 -1.375883 -1.633644
C     1.039153  1.503495 -1.484521
C     2.469812 -0.015512  0.253753
O     1.289236 -2.185558 -2.420787
O     1.270104  2.399894 -2.178145
O     3.590395 -0.027896  0.578301
C    -2.158693 -3.131375 -0.251310
C     0.886744 -1.330629  2.928641
C    -0.167384  3.089816  1.188899
C    -4.131279  1.228133 -0.777407
H    -2.245229 -3.801237  0.616457
H    -1.326649 -3.510010 -0.862654
H    -3.076537 -3.213097 -0.841679
H    -5.010195  0.822794 -0.254662
H    -4.211863  0.900658 -1.824313
H    -4.196561  2.320390 -0.755391
H    -0.845723  3.650843  1.848827
H     0.837227  3.118470  1.621909
H    -0.133460  3.618882  0.229365
H     0.339916 -1.468414  3.872964
H     1.258211 -2.311497  2.614286
H     1.750565 -0.688155  3.132470
H    -3.616492 -1.160993 -0.767878
H    -2.284528  2.668956  0.098017
H     0.677340  1.145463  2.536522
H    -0.205227 -2.622024  1.075472
    el energy= -874.724031045
          zpe= -874.452307
    th energy= -874.431922
  th enthalpy= -874.430978
  free energy= -874.501214
```

TS-D

```
C    -1.959928 -0.174092 -1.718057
```

C	-1.478126	-1.327766	-0.918823
C	-1.672170	-1.580617	0.411995
C	-2.313429	-0.646979	1.359093
C	-1.960661	0.630285	1.612659
C	-0.875083	1.391877	0.927201
C	-0.828364	1.734510	-0.438431
C	-1.681627	1.130313	-1.477755
W	0.693294	-0.113965	0.039563
C	1.780446	-1.775566	-0.143241
C	1.867146	0.750018	-1.230380
C	1.963223	0.507532	1.361853
O	2.405576	-2.749282	-0.234741
O	2.533701	1.286299	-2.024871
O	2.693508	0.883603	2.192253
C	-2.741549	-0.580455	-2.948145
C	-1.364310	-2.953250	0.977977
C	-2.632044	1.399426	2.727677
C	-0.191825	3.054224	-0.842905
H	-3.656894	-1.124397	-2.674014
H	-2.150607	-1.257840	-3.582106
H	-3.024847	0.289361	-3.550477
H	-0.972202	3.825338	-0.946560
H	0.335710	2.985465	-1.800713
H	0.522716	3.396160	-0.087325
H	-3.089289	2.321897	2.342958
H	-3.407295	0.806959	3.225769
H	-1.893859	1.706282	3.482508
H	-2.305201	-3.447694	1.263773
H	-0.848941	-3.590075	0.252015
H	-0.752396	-2.889489	1.885838
H	-2.036287	1.841996	-2.227115
H	-0.382765	2.105215	1.589464
H	-3.080152	-1.100867	1.990187
H	-1.093912	-2.156606	-1.517278

el energy= -874.716840926
zpe= -874.446552
th energy= -874.425843
th enthalpy= -874.424899
free energy= -874.496189

TS-E

C	-0.268465	1.209032	1.678362
C	0.099769	2.507335	1.232973
C	0.108745	3.166859	0.000000
C	0.099769	2.507335	-1.232973
C	-0.268465	1.209032	-1.678362
C	-1.402000	0.415982	-1.260573
C	-2.024678	0.186874	0.000000
C	-1.402000	0.415982	1.260573
W	0.188973	-0.632015	0.000000
C	0.109837	-2.121987	1.297987
C	0.109837	-2.121987	-1.297987
C	2.219062	-0.621961	0.000000
O	0.040121	-3.010795	2.043054
O	0.040121	-3.010795	-2.043054
O	3.371645	-0.574553	0.000000
C	0.178484	0.896514	3.113371
C	0.419718	4.654111	0.000000
C	0.178484	0.896514	-3.113371
C	-3.339916	-0.571875	0.000000
H	-0.480842	1.381107	3.851018
H	1.199823	1.247498	3.297887
H	0.165453	-0.179645	3.315630
H	-4.184921	0.131517	0.000000
H	-3.437878	-1.207354	0.887647
H	-3.437878	-1.207354	-0.887647
H	-0.480842	1.381107	-3.851018
H	1.199823	1.247498	-3.297887
H	0.165453	-0.179645	-3.315630
H	0.995126	4.948076	0.887527
H	0.995126	4.948076	-0.887527
H	-0.498556	5.259192	0.000000
H	-1.892045	-0.108889	2.082697
H	-1.892045	-0.108889	-2.082697
H	0.538001	3.109688	-2.035801
H	0.538001	3.109688	2.035801

el energy= -874.717102870
zpe= -874.447974
th energy= -874.426834
th enthalpy= -874.425889
free energy= -874.498107

TS-F

C	0.530102	1.309985	1.622744
C	0.240007	0.003098	2.101642
C	0.500410	-1.309777	1.614689
C	1.679976	-1.765205	0.828608
C	2.072333	-1.264805	-0.354645
C	1.426360	-0.013268	-0.913045
C	2.077270	1.240608	-0.370012
C	1.698655	1.750647	0.814971
W	-0.665436	-0.001586	-0.076512
C	-1.737185	1.637365	-0.398325
C	-0.883953	0.016967	-2.057176
C	-1.744999	-1.627218	-0.423525
O	-2.417414	2.573297	-0.483990
O	-0.960015	0.024560	-3.214821
O	-2.432476	-2.556965	-0.522905
C	-0.107678	2.463661	2.391059
C	-0.158305	-2.451917	2.383293
C	3.168143	-1.903335	-1.170947
C	3.154181	1.883183	-1.208012
H	-1.022169	2.157797	2.912543
H	-0.361533	3.297334	1.726352
H	0.592262	2.852704	3.147151
H	3.565533	2.774854	-0.720433
H	2.753770	2.184101	-2.187540
H	3.983692	1.190152	-1.408480
H	4.000321	-1.208192	-1.352787
H	3.571383	-2.793958	-0.674669
H	2.789995	-2.205168	-2.158943
H	0.528409	-2.845369	3.149307
H	-1.075004	-2.132769	2.893170
H	-0.415368	-3.285832	1.720156
H	2.194891	2.651960	1.182503
H	1.531464	-0.019977	-2.002537
H	2.175343	-2.658849	1.216109
H	-0.583404	0.011491	2.825805

el energy= -874.684131689
zpe= -874.414575
th energy= -874.394062
th enthalpy= -874.393118

free energy= -874.463380

TS-G

```
C     1.339820 -1.276830 -0.942752
C     1.409193  0.000064 -1.563482
C     1.339860  1.276922 -0.942712
C     1.845535  1.773706  0.368865
C     1.470503  1.242325  1.544886
C     0.637879 -0.000041  1.720998
C     1.470599 -1.242340  1.544824
C     1.845625 -1.773647  0.368771
W    -0.583402 -0.000023 -0.235001
C    -1.668217 -1.598034 -0.663602
C    -2.015538  0.000020  1.150332
C    -1.668247  1.597969 -0.663679
O    -2.289090 -2.503025 -1.041086
O    -2.862772  0.000035  1.943977
O    -2.289125  2.502934 -1.041194
H     1.218158 -2.087464 -1.662560
H     1.218201  2.087586 -1.662484
H     1.799782  1.726375  2.468290
H     1.799980 -1.726364  2.468206
C     2.711646 -3.008441  0.293473
C     0.003759 -0.000103  3.117098
C     2.711416  3.008604  0.293665
C     1.215695  0.000084 -3.080843
H     2.992682 -3.363610  1.291384
H     2.191911 -3.827633 -0.225374
H     3.632329 -2.809804 -0.274655
H     0.682816  0.889574 -3.434201
H     2.207642 -0.000051 -3.555175
H     0.682591 -0.889277 -3.434192
H     2.992338  3.363779  1.291606
H     3.632164  2.810092 -0.274400
H     2.191625  3.827749 -0.225197
H    -0.616946 -0.887050  3.282670
H     0.794183 -0.000119  3.884812
H    -0.616978  0.886806  3.282741
    el energy= -874.680149928
          zpe= -874.410540
    th energy= -874.390095
  th enthalpy= -874.389151
  free energy= -874.459206
```

References

(1) Cotton, F. A.; Faller, J. W.; Musco, A. Stereochemically Nonrigid Organometallic Compounds. II. 1,3,5,7-Tetramethylcyclo-Octatetraenemolybdenum Tricarbonyl. *J. Am. Chem. Soc.* **1966**, *88* (19), 4506–4507. https://doi.org/10.1021/ja00971a037.

(2) Bennett, M. J.; Cotton, F. A.; Takats, J. Stereochemically Nonrigid Organometallic Molecules. XI. Molecular Structure of (1,3,5,7-Tetramethylcyclooctatetraene)Chromium Tricarbonyl. *J. Am. Chem. Soc.* **1968**, *90* (4), 903–909. https://doi.org/10.1021/ja01006a011.
